# Supplementary material for: Sirtuin-1 sensitive lysine-136 acetylation drives phase separation and pathological aggregation of TDP-43
Source: Nat Commun. 2022 Mar 9;13:1223. doi: 10.1038/s41467-022-28822-7 (PMC8907366; doi:10.1038/s41467-022-28822-7)
Supplement: Supplementary file 1 — Supplementary Information [file 41467_2022_28822_MOESM1_ESM.pdf]

Supplementary Table 1. TDP-43 peptides found by MS to be acetylated. Longer chain lysine modifications like propionylation and butyrylation were not detected.

Peptide sequence	Peptide lysine	TDP-43 residue	Mascot identity score
RLVEGILHAPDAGWGNLVYVVNYPKDNKR	K25	K79	27.2
RKMDETDASSAVKV	K2	K84	28.3
RAVQKTSDLIVLGLPWKT	K5	K121	25.0
KEYFSTFGEVLMVQVKKD	K17	K136	30.0

Supplementary Table 2. List of mutagenesis and cloning primers.

**Primer**	**Sequence**
TDP∆GRD NotI reverse	ccccgcggccgcctaacttctttctaactgtctattgctattg
TDP-43 6His BamHI reverse	cagcggccgcggatccttaatggtgatggtgatgatgcattccccagccagaag
TDP-43 6His NheI forward	gctagccatcatcaccatcaccatatgtctgaatatattc
TDP-43 BamHI forward	ggggggatccgatgtctgaatatattcgggtaacc
TDP-43 Bsp120 forward	gggggggcccaccatgtctgaatatattcgggtaaccg
TDP-43 HindIII reverse	ccccaagcttctacattccccagccagaag
TDP43 K121Q forward	ccgaacaggacctgcaagagtattttagtac
TDP43 K121Q reverse	gtactaaaatactcttgcaggtcctgttcgg
TDP43 K121R forward	ccgaacaggacctgagagagtattttagtac
TDP43 K121R reverse	gtactaaaatactctctcaggtcctgttcgg
TDP43 K136Q forward	ggtgcaggtccagaaagatcttaagactgg
TDP43 K136Q reverse	ccagtcttaagatctttctggacctgcacc
TDP43 K136R forward	ggtgcaggtcaggaaagatcttaagactgg
TDP43 K136R reverse	ccagtcttaagatctttcctgacctgcacc
TDP-43 K136TAG forward	cttatggtgcaggtctagaaagatcttaagact
TDP-43 K136TAG reverse	agtcttaagatctttctagacctgcaccataag
TDP43 K145Q forward	gactggtcattcacaggggtttggctttg
TDP43 K145Q reverse	caaagccaaacccctgtgaatgaccagtc
TDP43 K145R forward	gactggtcattcaagggggtttggctttg
TDP43 K145R reverse	caaagccaaacccccttgaatgaccagtc
TDP-43 K79Q forward	gtcaactatccacaagataacaaaagaaaaatg
TDP-43 K79Q reverse	catttttcttttgttatcttgtggatagttgac
TDP-43 K79R forward	gtcaactatccaagagataacaaaagaaaaatg
TDP-43 K79R reverse	catttttcttttgttatctcttggatagttgac
TDP43 K84Q forward	gataacaaaagacaaatggatgagacag
TDP43 K84Q reverse	ctgtctcatccatttgtcttttgttatc
TDP-43 K84R forward	ccaaaagataacaaaagaagaatggatgagacag
TDP-43 K84R reverse	ctgtctcatccattcttcttttgttatcttttgg
TDP-43 K84TAG forward	ccaaaagataacaaaagatagatggatgagacagat
TDP-43 K84TAG reverse	atctgtctcatccatctatcttttgttatcttttgg
TDP-43 NheI forward	ctactctagagctagcatgtctgaatatatt
TDP-43 NotI reverse	ccccgcggccgcctacattccccagccagaag
TDP-43 SalI forward	ggggtcgacgatgtctgaatatattcgggtaaccg

Supplementary figure 1

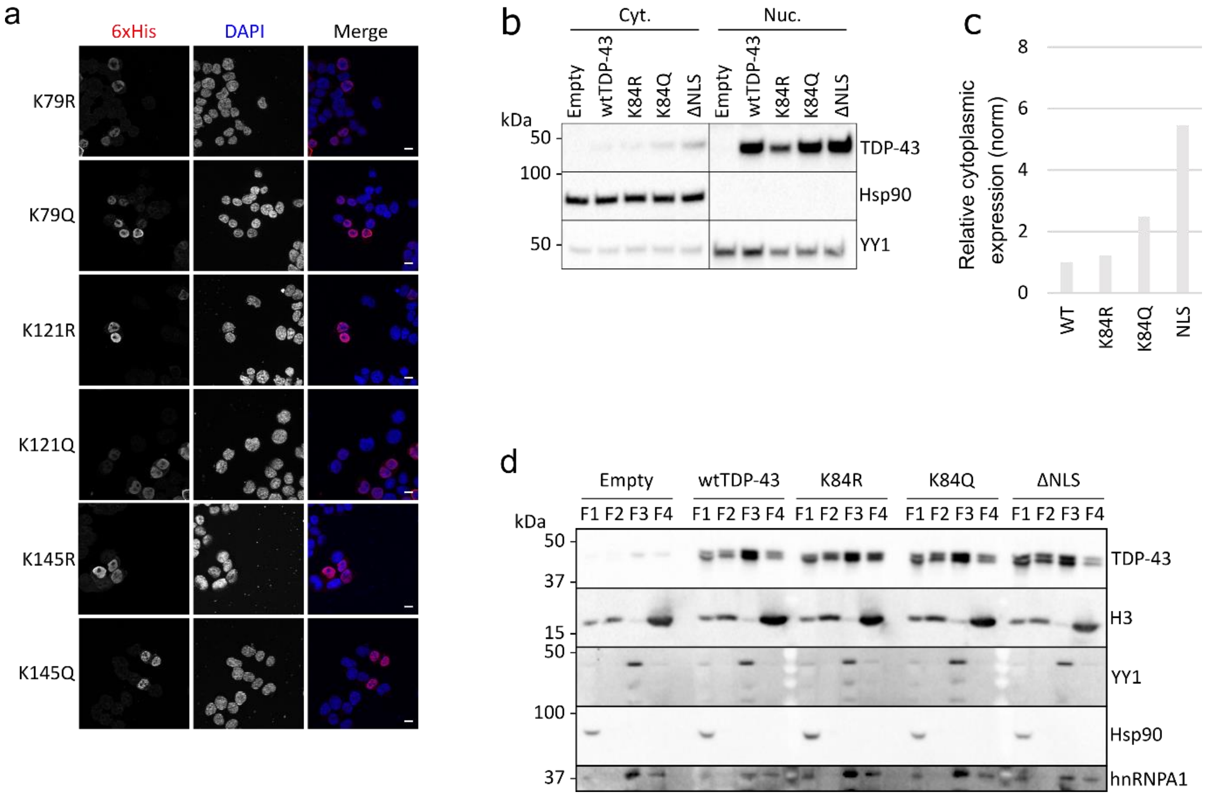

Supplementary Figure 1. Nuclear cytoplasmic distribution of TDP-43 is disturbed in K84Q mutants. **a** Immunostaining of HEK293E cells previously transfected with 6xHis tagged non acetyl-mimics (K79R, K121R and K145R) or acetyl-mimics (K79Q, K121Q and K145Q). Scale bar represents 10 µm. N = 3 biologically independent experiments. **b** Cytoplasmic and nuclear fractions from HEK293E cells transfected with 6xHis tagged wt, K84R, K84Q or Flag tagged ΔNLS TDP-43. These fractions were analyzed by Western blot. 6 and 3,5µg of protein from the cytoplasmic and nuclear fraction was loaded, respectively. N = 2 biologically independent experiments. Hsp90 and YY1 were used as cytoplasmic and nuclear markers respectively. Source data are provided as a Source Data file. **c** Quantification of the proportion of TDP-43 in the cytoplasm normalized to wtTDP-43. N = 2 biologically independent experiments. * = $p < 0.05$, ** = $p < 0.01$. Source data are provided as a Source Data file. **d** Subcellular protein fractionation of different TDP-43 constructs. Cells were transfected with 6xHis tagged TDP-43 and proteins were fractionated in four fractions using a subcellular protein fractionation kit for cultured cells (Thermo Fisher) according to manufacturer's instructions: F1 (cytoplasmic soluble), F2 (membrane bound), F3 (nuclear soluble) and F4

(histone bound). Hsp90, YY1 and H3 were used as markers for the cytoplasmic, nuclear soluble and histone bound fractions, respectively. N = 2 biologically independent experiments. Source data are provided as a Source Data file.

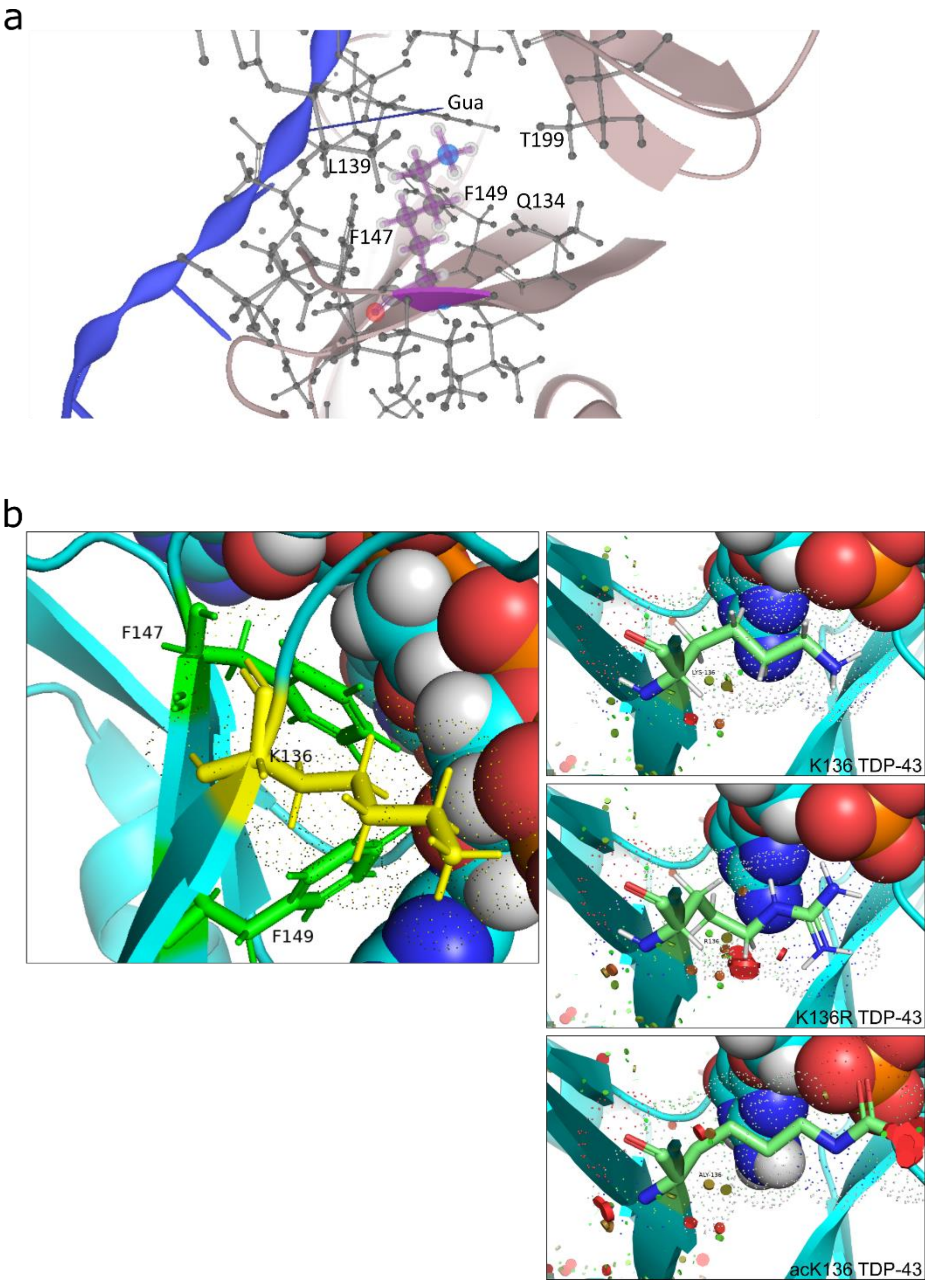

Supplementary Figure 2. Protein structures of TDP-43 RRM1 RNA binding pocket. **a** Structure of TDP-43 RRM1 in contact with RNA (PDB: 4BS2) with highlighted K136 atoms. **b** Close-up view of TDP-43 with

different 136 amino acid substitutions. Green circles represent points of contact between TDP-43 amino acids. Red circles represent clashes between residue 136 and other objects.

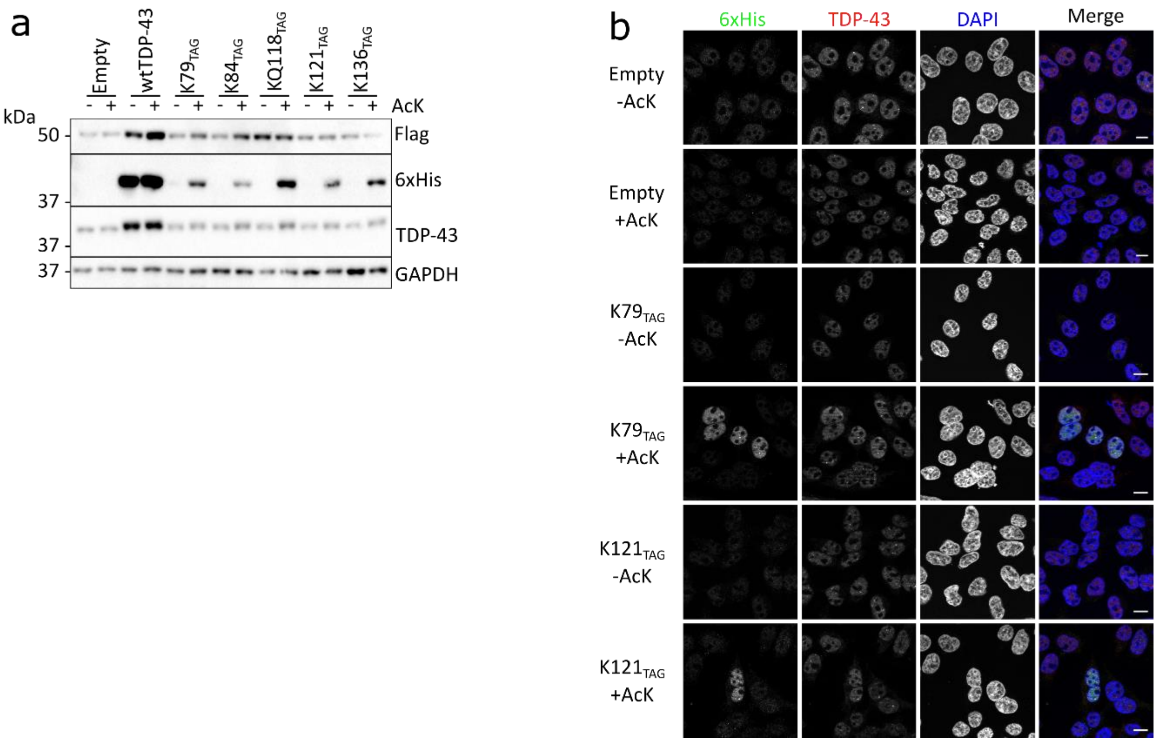

Supplementary Figure 3. Amber suppression of TDP-43 at K79 and K121 does not impact its subcellular distribution. **a** Western blot of HEK293E cells transfected with E451 containing a Flag-tagged acKRS, RNA_{TAG} and different C-terminal 6xHis-tagged TDP-43 constructs. Cells were lysed after 24h in the presence of 5mM acK in the media. N = 3 biological replicates. Source data are provided as a Source Data file. **b** Double immunostaining of HEK293E cells transfected with Flag-tagged acKRS, RNA_{TAG} and different C-terminal 6xHis-tagged TDP-43 constructs. Cells were fixed after 24h in the presence of 5mM acK in the media. Scale bar represents 10µm. N = 3 biological replicates. Source data are provided as a Source Data file.

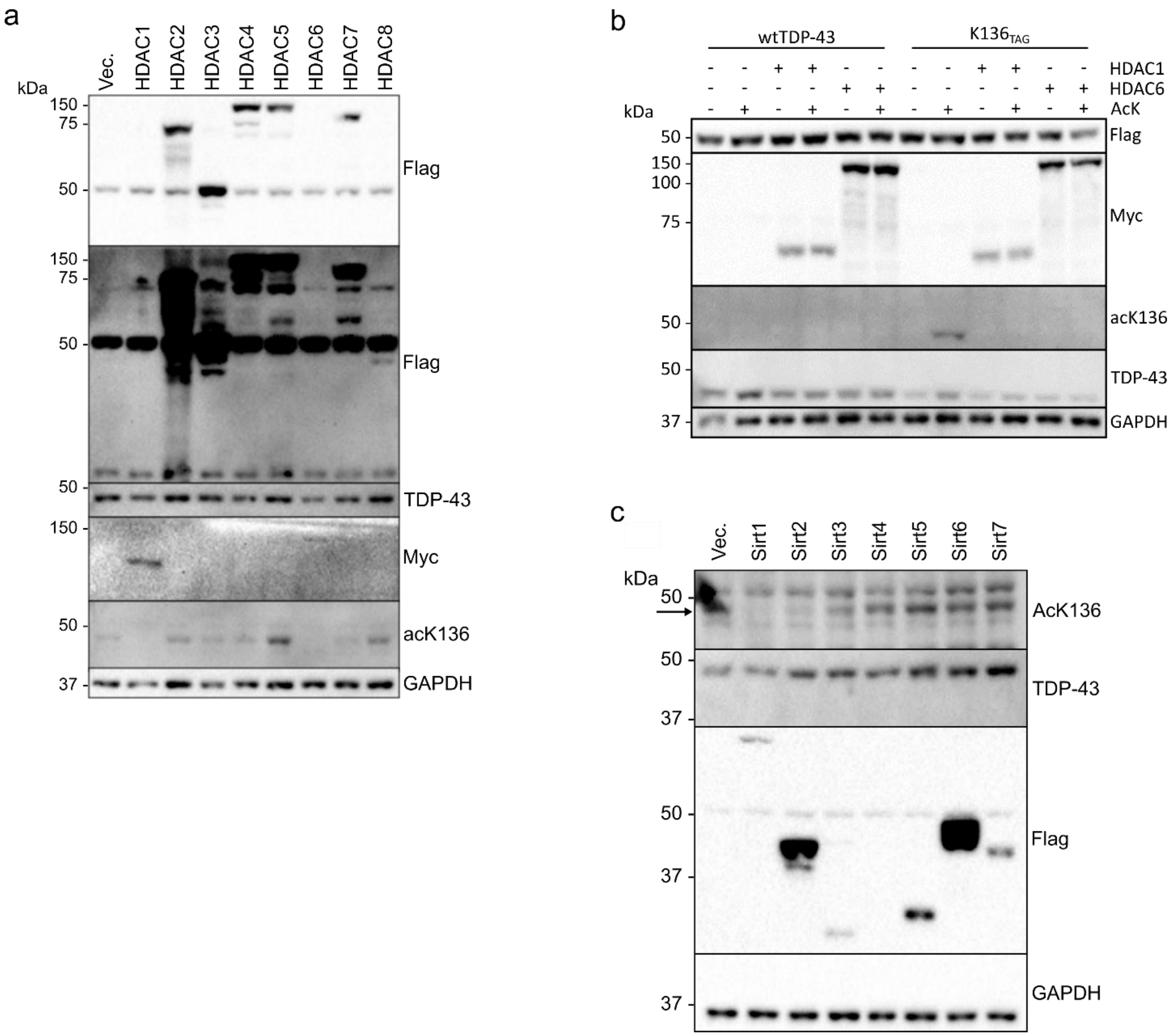

Supplementary Figure 4. HDAC and Sirtuin effect on K136 acetylation of TDP-43. **a** Stably amber suppressed $sh^{TDP\text{-}43}$-HEK293E cells were cotransfected with HDAC1-8 constructs together with $K136_{TAG}$ TDP-43. Cells were lysed after 24h in the presence of 5mM acK. Protein levels were assessed via Western blot. HDAC1 and 6 were myc-tagged, while HDAC2, 3, 4, 5, 7 and 8 were Flag tagged. N = 2. Source data are provided as a Source Data file. **b** Stably amber suppressed $sh^{TDP\text{-}43}$-HEK293E cells were cotransfected with HDAC1 or HDAC6 constructs together with $K136_{TAG}$ TDP-43 or wtTDP-43. Cells were lysed after 24h in the presence of 5mM acK. Protein levels were assessed via Western blot. N = 2. Source data are provided as a Source Data file. **c** Stably amber suppressed $sh^{TDP\text{-}43}$-HEK293E cells were cotransfected with sirtuin

constructs together with K136$_{TAG}$ TDP-43. Cells were lysed after 24h in the presence of 5mM acK. Protein levels were assessed via Western blot. N = 3. Source data are provided as a Source Data file.

Supplementary video 1 and 2. Live imaging of HEK293E cells 48 hours after transfection with [K136Q]TDP-43-EGFP. Cells were kept at 37°C and 5% CO_2 during the experiment. Imaging took place every 5min for 4 hours.

Supplementary video 3. Live imaging of HEK293E cells 48h after transfection with [K136Q]TDP-43-EGFP. Cells were kept at 37°C and 5% CO_2 during the experiment. Imaging took place every 2min for 1 hour.
