# Supplementary material for: Sirtuin-1 sensitive lysine-136 acetylation drives phase separation and pathological aggregation of TDP-43
Source: Nat Commun. 2022 Mar 9;13:1223. doi: 10.1038/s41467-022-28822-7 (PMC8907366; doi:10.1038/s41467-022-28822-7)
Supplement: Supplementary file 6 — Reporting Summary [file 41467_2022_28822_MOESM6_ESM.pdf]

nature portfolio

Corresponding author(s): Philipp J. Kahle

Last updated by author(s): Dec 1, 2021

Reporting Summary

Nature Portfolio wishes to improve the reproducibility of the work that we publish. This form provides structure for consistency and transparency in reporting. For further information on Nature Portfolio policies, see our Editorial Policies and the Editorial Policy Checklist.

Statistics

For all statistical analyses, confirm that the following items are present in the figure legend, table legend, main text, or Methods section.

n/a	Confirmed	
☐	☒	The exact sample size (*n*) for each experimental group/condition, given as a discrete number and unit of measurement
☐	☒	A statement on whether measurements were taken from distinct samples or whether the same sample was measured repeatedly
☐	☒	The statistical test(s) used AND whether they are one- or two-sided<br>*Only common tests should be described solely by name; describe more complex techniques in the Methods section.*
☐	☒	A description of all covariates tested
☐	☒	A description of any assumptions or corrections, such as tests of normality and adjustment for multiple comparisons
☐	☒	A full description of the statistical parameters including central tendency (e.g. means) or other basic estimates (e.g. regression coefficient) AND variation (e.g. standard deviation) or associated estimates of uncertainty (e.g. confidence intervals)
☐	☒	For null hypothesis testing, the test statistic (e.g. *F*, *t*, *r*) with confidence intervals, effect sizes, degrees of freedom and *P* value noted<br>*Give P values as exact values whenever suitable.*
☒	☐	For Bayesian analysis, information on the choice of priors and Markov chain Monte Carlo settings
☒	☐	For hierarchical and complex designs, identification of the appropriate level for tests and full reporting of outcomes
☒	☐	Estimates of effect sizes (e.g. Cohen's *d*, Pearson's *r*), indicating how they were calculated

Our web collection on statistics for biologists contains articles on many of the points above.

Software and code

Policy information about availability of computer code

Data collection	Image Lab 5.0 (bioRad), Zen Blue edition 2.3 (Zeiss), Pymol 2.4.0 with PyTMs.
Data analysis	Zen Blue edition 2.3 (Zeiss), ImageJ 1.52a, Cell Profiler 4.1.3, Prism 9.3.0 (Graphpad), Microsoft Excel 2018.

For manuscripts utilizing custom algorithms or software that are central to the research but not yet described in published literature, software must be made available to editors and reviewers. We strongly encourage code deposition in a community repository (e.g. GitHub). See the Nature Portfolio guidelines for submitting code & software for further information.

Data

Policy information about availability of data

All manuscripts must include a data availability statement. This statement should provide the following information, where applicable:

- Accession codes, unique identifiers, or web links for publicly available datasets
- A description of any restrictions on data availability
- For clinical datasets or third party data, please ensure that the statement adheres to our policy

The manuscript now contains a data availability statement. Representative blots and photomicrographs are provided with the paper. Unprocessed raw data used for statistical analysis has been provided. Uncropped Western blots and gels have been provided to the journal. Protein structure data bases Swiss-Prot and Uniprot were used (https://www.expasy.org/resources/uniprotkb-swiss-prot).

March 2021

Field-specific reporting

Please select the one below that is the best fit for your research. If you are not sure, read the appropriate sections before making your selection.

☒ Life sciences ☐ Behavioural & social sciences ☐ Ecological, evolutionary & environmental sciences

For a reference copy of the document with all sections, see nature.com/documents/nr-reporting-summary-flat.pdf

Life sciences study design

All studies must disclose on these points even when the disclosure is negative.

Sample size	Sample size of 3 was chosen for Western blot, FRAP and splicing assay was chosen, which is sufficient for statistical significance.<br>The filter binding assay was performed in duplicates and repeated 3 times.<br>Nuclear cytoplasmic fractionation were done by two different methods, each repeated twice.<br>For immunofluorescence quantification, the smallest number of transfected cells per cover slip (n = 200) was chosen and two cover slips (N = 2) were quantified.<br>For the quantification of droplet size 100 cells per condition were analysed. This number was sufficient to capture the variability in droplet size among cells.
Data exclusions	Apoptotic cells were excluded from the immunofluorescence analysis. Clogged wells were excluded from the filter binding assay analysis.
Replication	Experiments were performed 3 independent times, unless stated otherwise, under similar conditions and reagents. All attempts of replication were successful.
Randomization	HEK293-E cells in suspension were distributed randomly in the different well/plates used for experiments.
Blinding	Experimenters were blinded only for the analysis of droplet size of figure 5e. Blinding for the rest of experiments was not applicable due to the lack of manpower. Experimental data was analysed by the respective experimenter.

Reporting for specific materials, systems and methods

We require information from authors about some types of materials, experimental systems and methods used in many studies. Here, indicate whether each material, system or method listed is relevant to your study. If you are not sure if a list item applies to your research, read the appropriate section before selecting a response.

Materials & experimental systems

n/a	Involved in the study	
☐	☒	Antibodies
☐	☒	Eukaryotic cell lines
☒	☐	Palaeontology and archaeology
☐	☒	Animals and other organisms
☒	☐	Human research participants
☒	☐	Clinical data
☒	☐	Dual use research of concern

Methods

n/a	Involved in the study	
☒	☐	ChIP-seq
☒	☐	Flow cytometry
☒	☐	MRI-based neuroimaging

Antibodies

Antibodies used	In this study the following antibodies were used for Western blot (WB) and immunofluorescence staining (IF): rat anti-[acK84]TDP-43 (WB, 1:10 supernatant; IF,1:2; this work), rat anti-[acK136]TDP-43 (WB, 1:10 supernatant; IF,1:2; this work; mouse anti-6xHis (WB, 1:10000; IF, 1:1000; Amersham 27-4710-01), rabbit pan-acetylated lysine (WB, 1:1000; Cell Signalling #9441), rabbit mAb mix pan-acetylated lysine (WB, 1:1000; Ac-K-100, Cell Signalling #9814), mouse anti-Flag HRP-coupled (WB, 1:10000; Sigma #A8592), mouse anti-GAPDH (WB, 1:50000; clone 6C5, Biodesign International #H86504M), rabbit anti-Hsp90 (WB, 1:1000; Cell Signalling #4874), rat anti-[pS409/410]TDP-43 (WB, 1:10; IF, 1:50; clone 1D3), rabbit anti-TDP-43 (WB, 1:8000; IF, 1:1000; ProteinTech 10782-2-AP), mouse anti-TDP-43 (WB, 1:2000; IF, 1:1000; Abnova #H00023435), mouse anti-tubulin (WB, 1:10000; Sigma #T-5168), mouse anti-ubiquitin (WB, 1:4000; Millipore #MAB1510), mouse anti-YY1 (WB, 1:2000; clone H-10, Santa Cruz Biotechnology #7341), mouse anti-hnRNPA1 (WB, 1:2000; clone R196, Cell Signaling #5380), mouse anti-β-actin (WB, 1:50000; clone AC-15, Sigma #A5441); rabbit anti-mouse HRP-coupled (WB, 1:10000, Amersham #NIF825); goat anti-rabbit HRP-coupled (WB, 1:10000, Amersham #NIF824); donkey anti-rat HRP-coupled (WB, 1:10000, Jackson Immunoresearch, 712-035-150) and secondary Alexa Fluor 488- (anti-Mouse: Cat # A32723; anti-Rabbit: Cat # A32731), 555- (anti-Mouse: Cat # A32727; anti-Rabbit: Cat # A32732), 647- (anti-Mouse: Cat # A32728; anti-Rabbit: Cat # A32733) conjugated antibodies for IF produced in goat were from Invitrogen (1:1000).
Validation	All antibodies were validated by the manufacturer. Antibodies against acetylated K84 TDP-43 and acetylated K136 TDP-43 were validated by ourselves as shown in the paper (Figure 6).<br>Primary antibodies used:

- mouse anti-6xHis. Amersham 27-4710-01. https://www.sigmaaldrich.com/DE/en/product/sigma/ge27471001
- rabbit anti-pan-acetylated lysine. Validated in WB and IF. Cell signalling #9441. https://www.cellsignal.de/products/primary-antibodies/acetylated-lysine-antibody/9441
- rabbit mAb mix pan-acetylated lysine. Validated in WB. Cell Signalling #9814. https://www.cellsignal.de/products/primary-antibodies/acetylated-lysine-ac-k-2-100-multimab-rabbit-mab-mix/9814?site-search-type=Products&N=4294956287&Ntt=acetylated+lysine&fromPage=plp
- Mouse anti-Flag HRP-coupled. Validated in WB. Sigma #A8592. https://www.sigmaaldrich.com/DE/en/product/sigma/a8592?context=product
- mouse anti-GAPDH. Biodesign International #H86504M. Validated in WB and IF. https://www.meridianbioscience.com/lifescience/pairs-table/glyceraldehyde-3-phosphate-dehydrogenase-gapdh/?country=US
- Rabbit anti-Hsp90. Validated in WB and IHC. Cell Signalling #4874. https://www.cellsignal.de/products/primary-antibodies/hsp90-antibody/4874
- Rat anti-phospho TDP-43 (Ser409/Ser410) Antibody, clone 1D3. Validated in WB, ELISA and IHC. https://www.merckmillipore.com/DE/en/product/Anti-phospho-TDP-43-Ser409-Ser410-Antibody-clone-1D3,MM_NF-MABN14?ReferrerURL=https%3A%2F%2Fwww.google.com%2F&bd=1
- Rabbit anti-TDP-43. ProteinTech 10782-2-AP. Validated in WB, IHC and IF. https://www.ptglab.com/products/TARDBP-Antibody-10782-2-AP.htm
- Mouse anti-TDP-43. Validated in WB, IHC and IF. Abnova #H00023435. http://www.abnova.com/products/products_detail.asp?catalog_id=H00023435-M01
- Mouse anti-tubulin. Sigma #T-5168. Validated in WB, and IF. https://www.sigmaaldrich.com/RU/en/product/sigma/t5168
- Mouse anti-ubiquitin. Millipore #MAB1510. Validated in WB and IHC. https://www.merckmillipore.com/DE/en/product/Anti-Ubiquitin-Antibody-clone-Ubi-1,MM_NF-MAB1510-I-100UG?ReferrerURL=https%3A%2F%2Fwww.google.com%2F&bd=1
- Mouse anti-YY1. Santa Cruz Biotechnology #7341. Validated in WB, IHC and IF. https://www.scbt.com/p/yy1-antibody-h-10
- Mouse anti-hnRNPA1. Cell Signaling #5380. Validated in WB and IF. https://www.cellsignal.de/products/primary-antibodies/hnrnp-a1-r196-antibody/5380
- Mouse anti-β-actin. Sigma #A5441. Validated in WB, IHC and IF. https://www.sigmaaldrich.com/US/en/product/sigma/a5441

Eukaryotic cell lines

Policy information about cell lines

Cell line source(s)	HEK293-E (originally from ATCC).
Authentication	Cells were not authenticated.
Mycoplasma contamination	Incubators were routinely tested for mycoplasma contamination and tested negative.
Commonly misidentified lines (See ICLAC register)	None were used.

Animals and other organisms

Policy information about studies involving animals; ARRIVE guidelines recommended for reporting animal research

Laboratory animals	For the generation of antibodies against acetylated K84 and K136 TDP-43, two female Lou/c rats aged 12 weeks were immunised.
Wild animals	None were used.
Field-collected samples	Not applicable.
Ethics oversight	The animals used for the antibody generation belonged to the monoclonal antibody facility at the Helmholtz Munich. Approval by Regierung von Oberbayern AZ 55.2-1-54-2531.6-4-99.

Note that full information on the approval of the study protocol must also be provided in the manuscript.
