# Supplementary figures and images for: Sirtuin-1 sensitive lysine-136 acetylation drives phase separation and pathological aggregation of TDP-43
Source: Nat Commun. 2022 Mar 9;13:1223. doi: 10.1038/s41467-022-28822-7 (PMC8907366; doi:10.1038/s41467-022-28822-7)

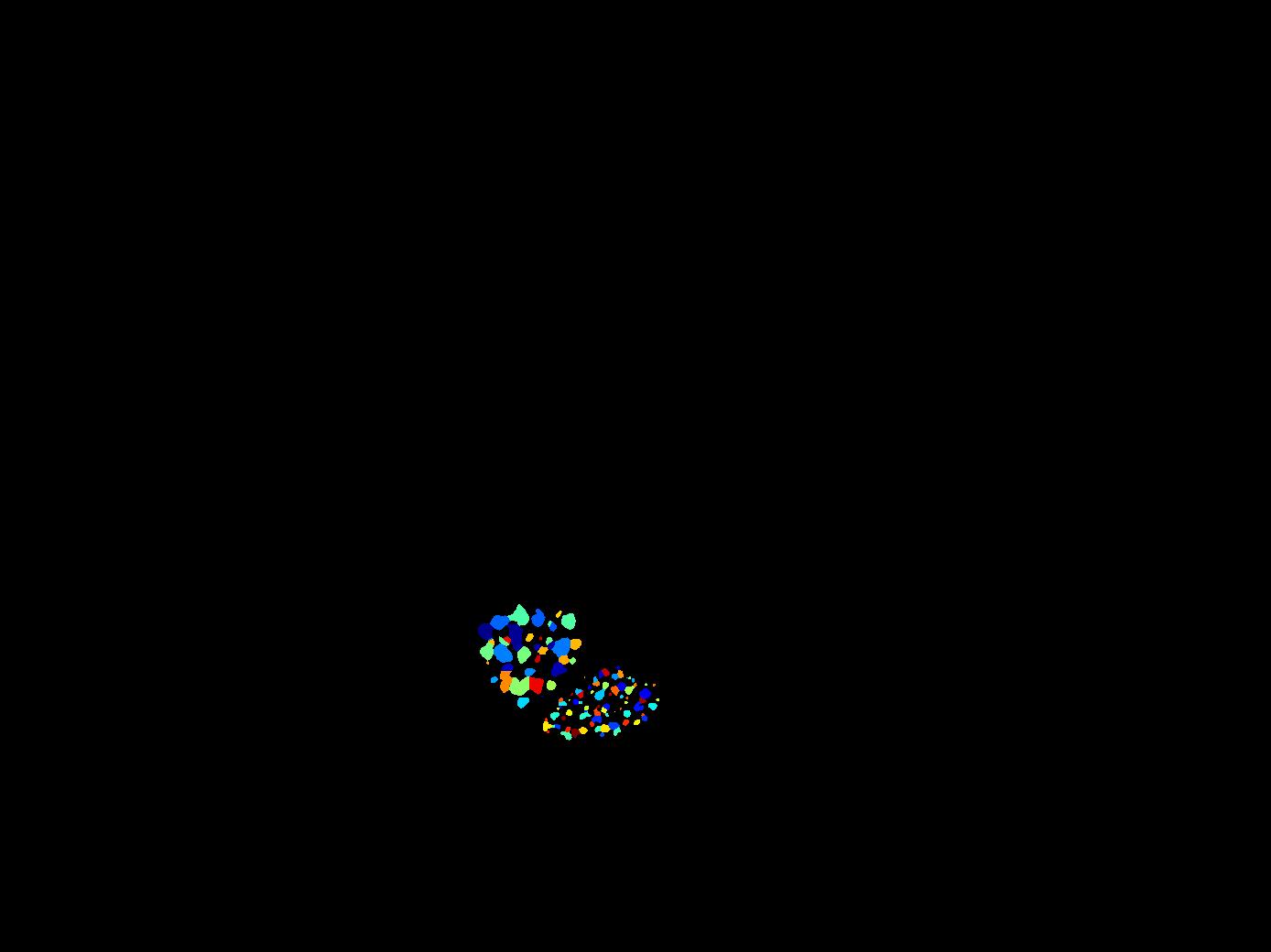

Supplement: Supplementary file 7 — Source Data [file 41467_2022_28822_MOESM7_ESM.zip › Figure 5E data/Masks/K136Q_I_01K136Q_24h 2_01_.jpeg]

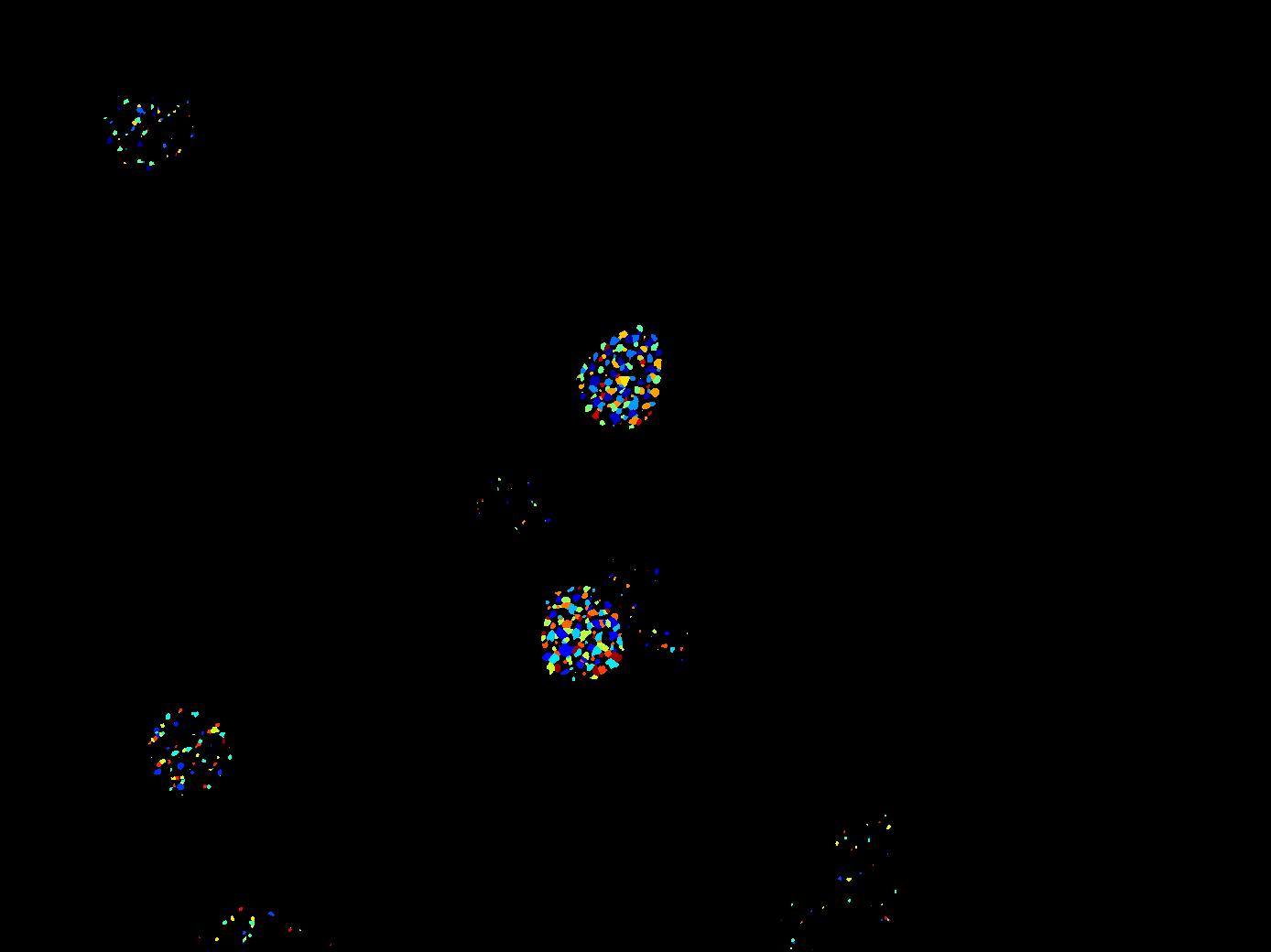

Supplement: Supplementary file 7 — Source Data [file 41467_2022_28822_MOESM7_ESM.zip › Figure 5E data/Masks/K136Q_I_01K136Q_24h_01_.jpeg]

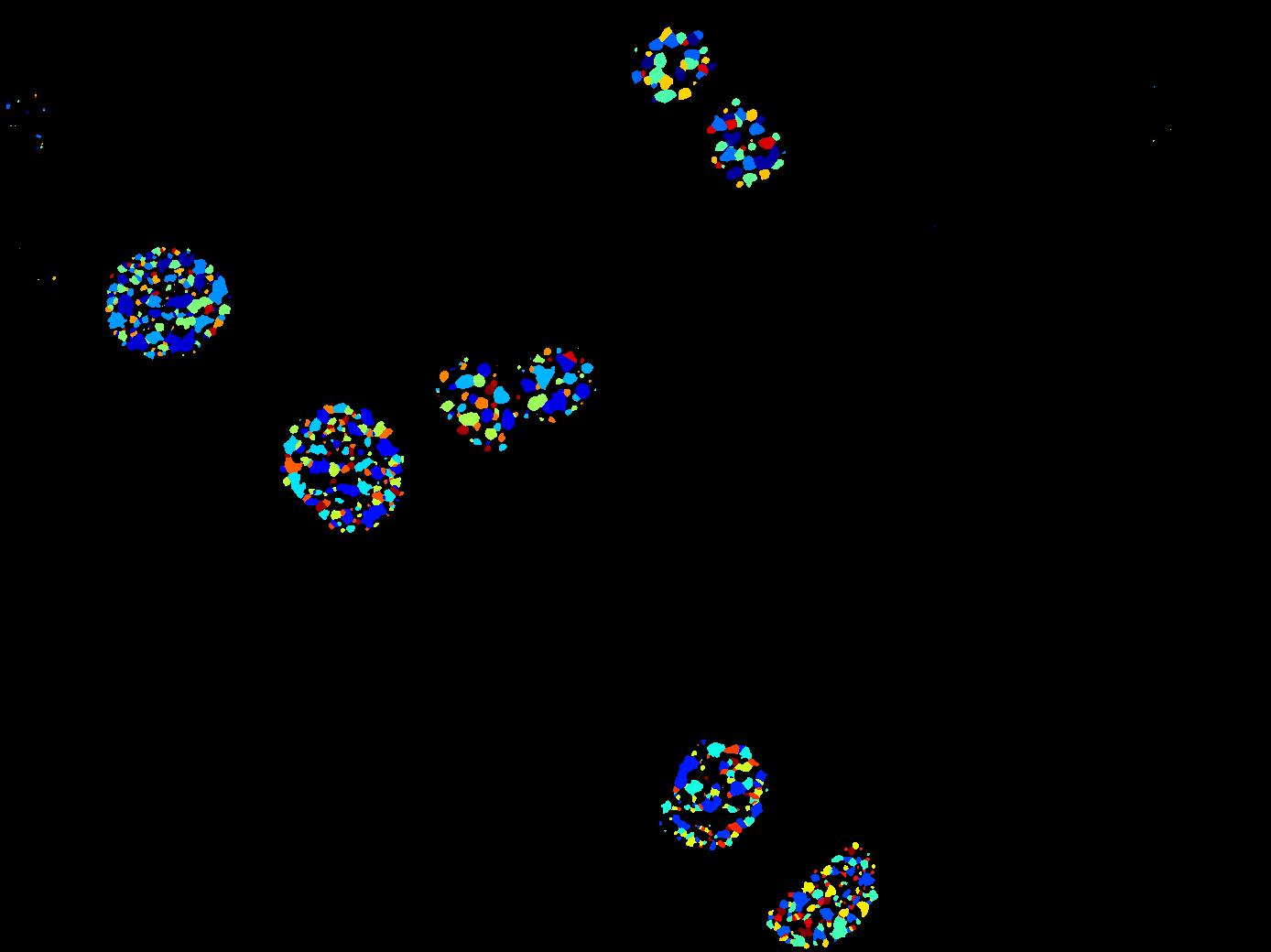

Supplement: Supplementary file 7 — Source Data [file 41467_2022_28822_MOESM7_ESM.zip › Figure 5E data/Masks/K136Q_I_01K136Q_48h 2_01_.jpeg]

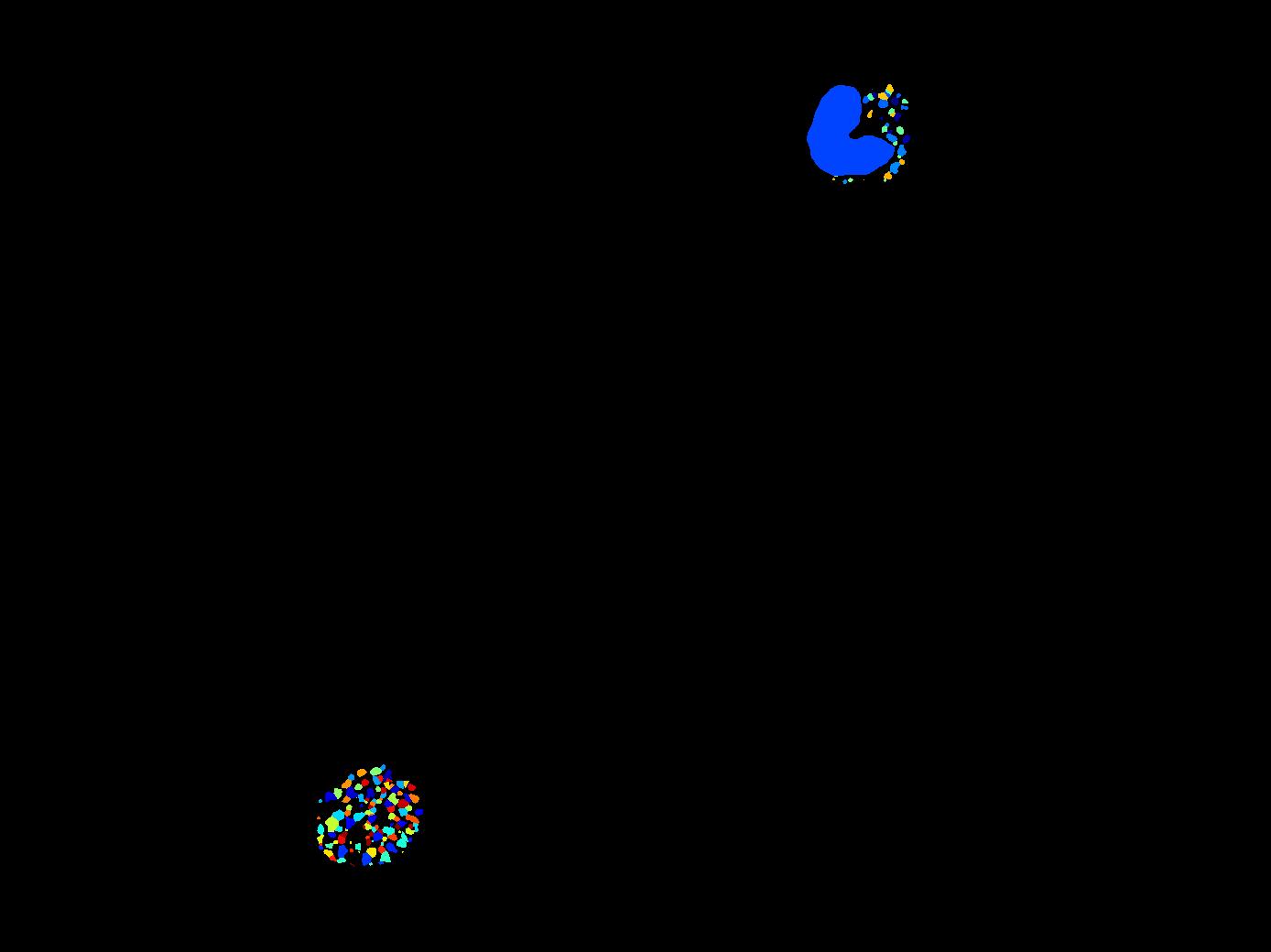

Supplement: Supplementary file 7 — Source Data [file 41467_2022_28822_MOESM7_ESM.zip › Figure 5E data/Masks/K136Q_I_01K136Q_48h_01_.jpeg]

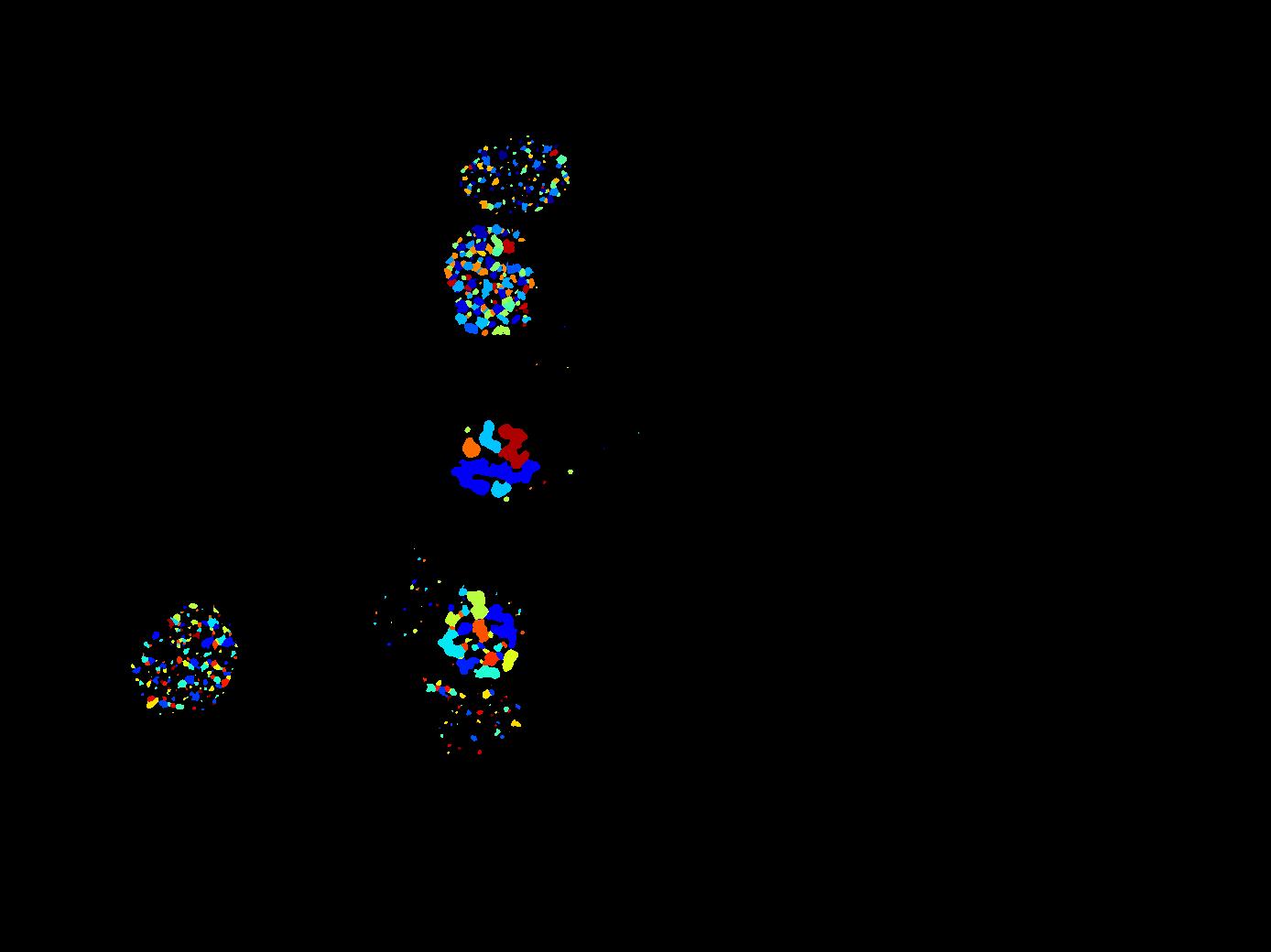

Supplement: Supplementary file 7 — Source Data [file 41467_2022_28822_MOESM7_ESM.zip › Figure 5E data/Masks/K136Q_I_01K136Q_72h 2_01_.jpeg]

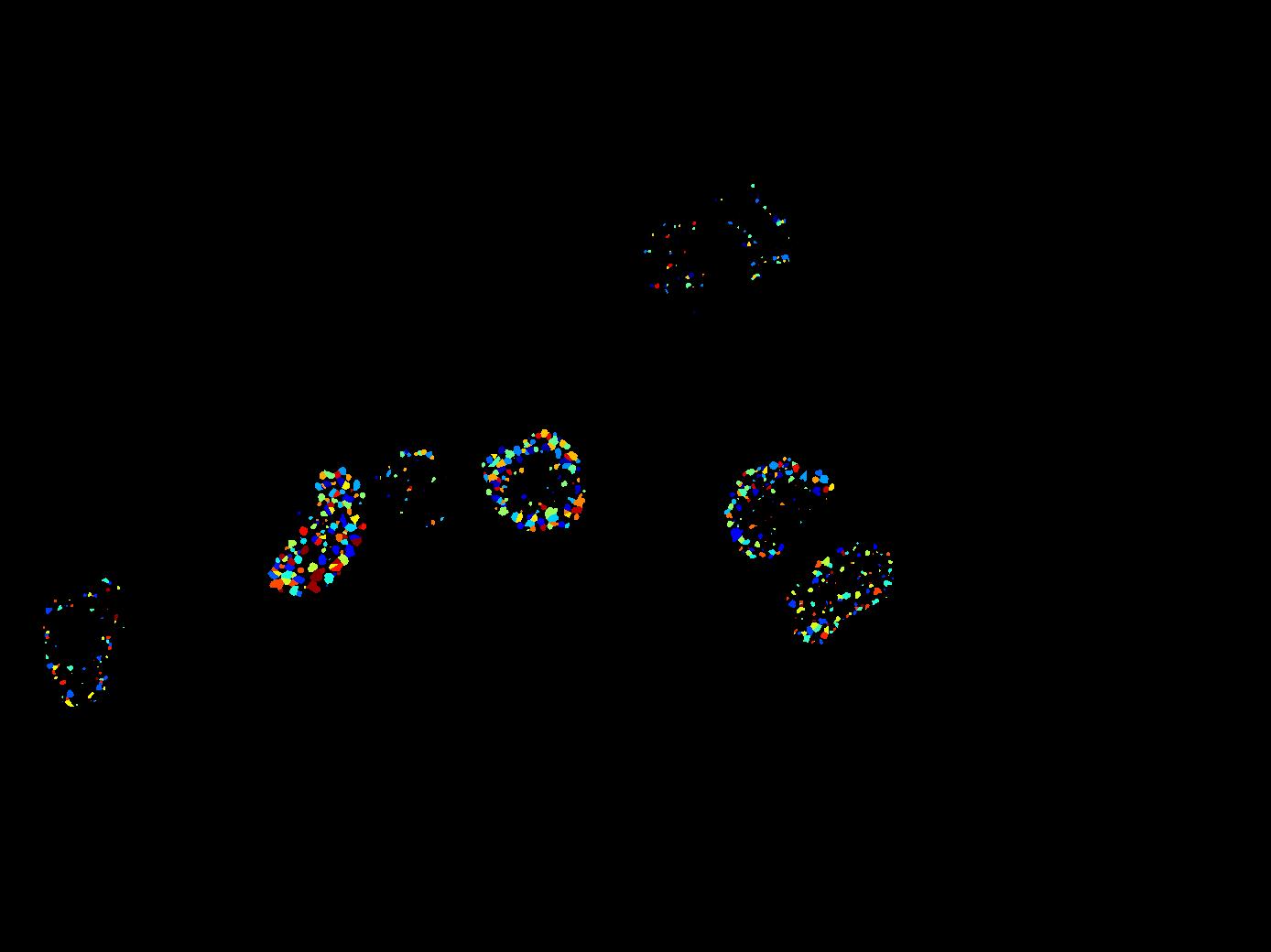

Supplement: Supplementary file 7 — Source Data [file 41467_2022_28822_MOESM7_ESM.zip › Figure 5E data/Masks/K136Q_I_01K136Q_72h_01_.jpeg]

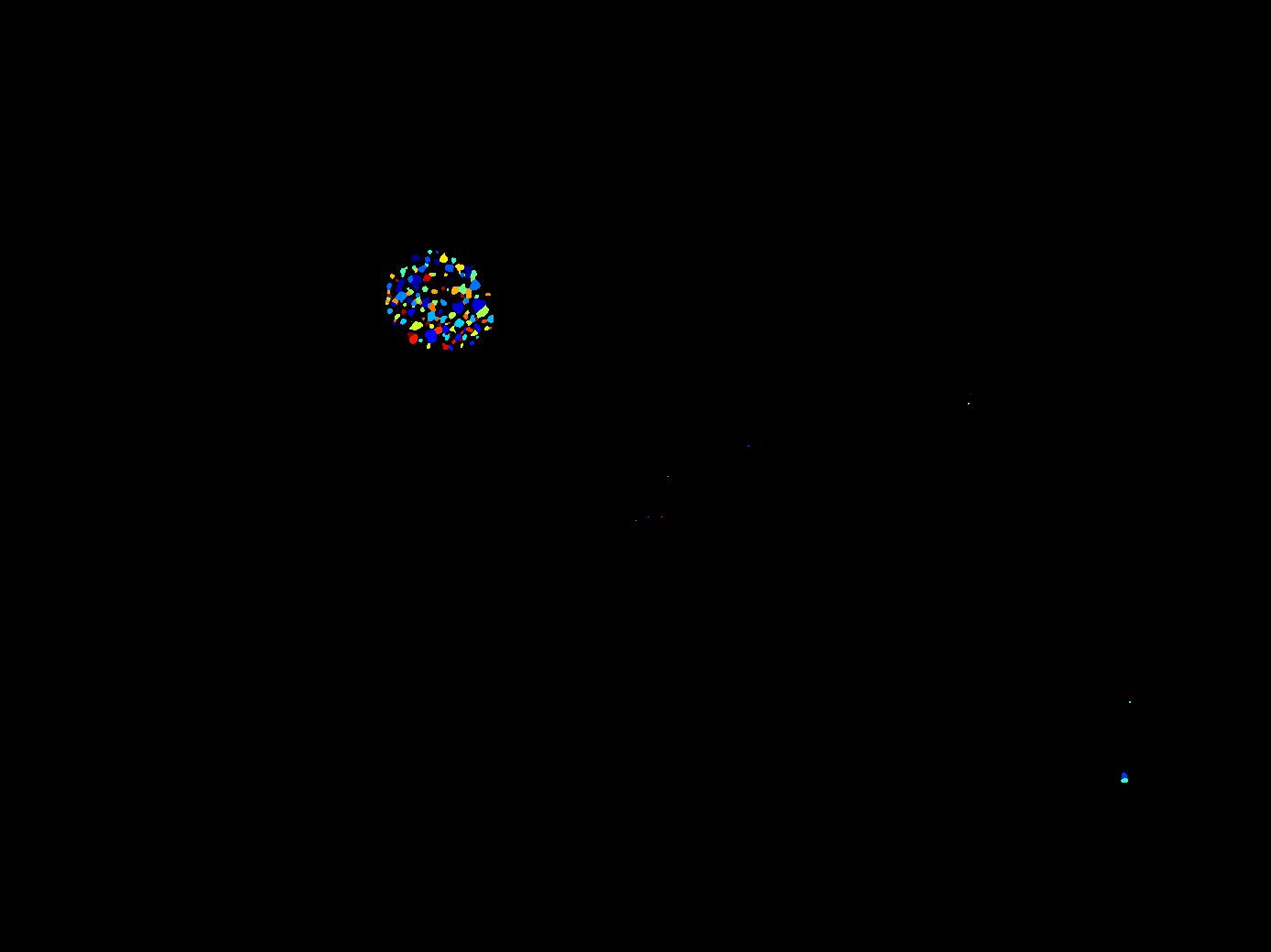

Supplement: Supplementary file 7 — Source Data [file 41467_2022_28822_MOESM7_ESM.zip › Figure 5E data/Masks/K136Q_I_02K136Q_24h 2_02_.jpeg]

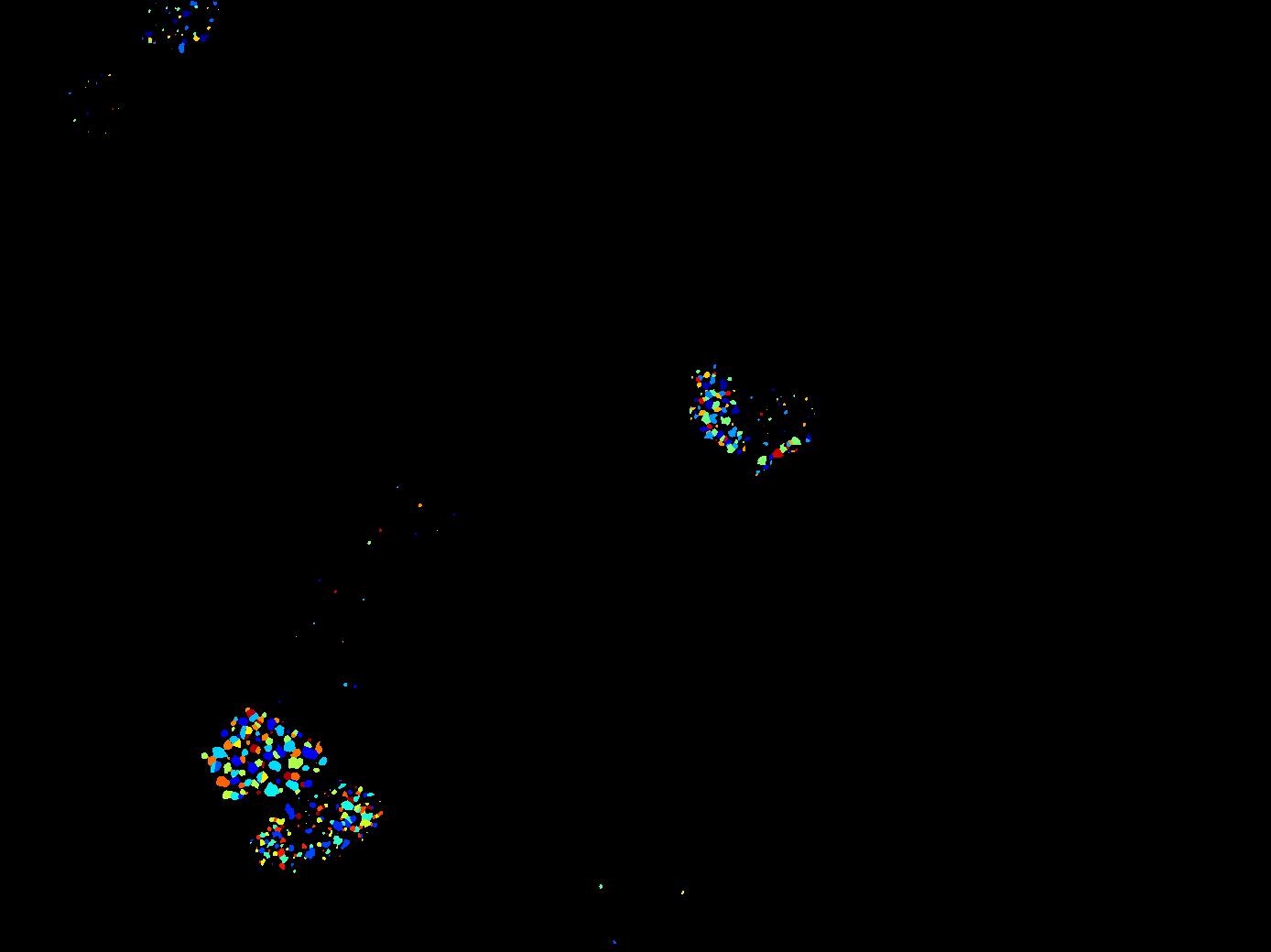

Supplement: Supplementary file 7 — Source Data [file 41467_2022_28822_MOESM7_ESM.zip › Figure 5E data/Masks/K136Q_I_02K136Q_24h_02_.jpeg]

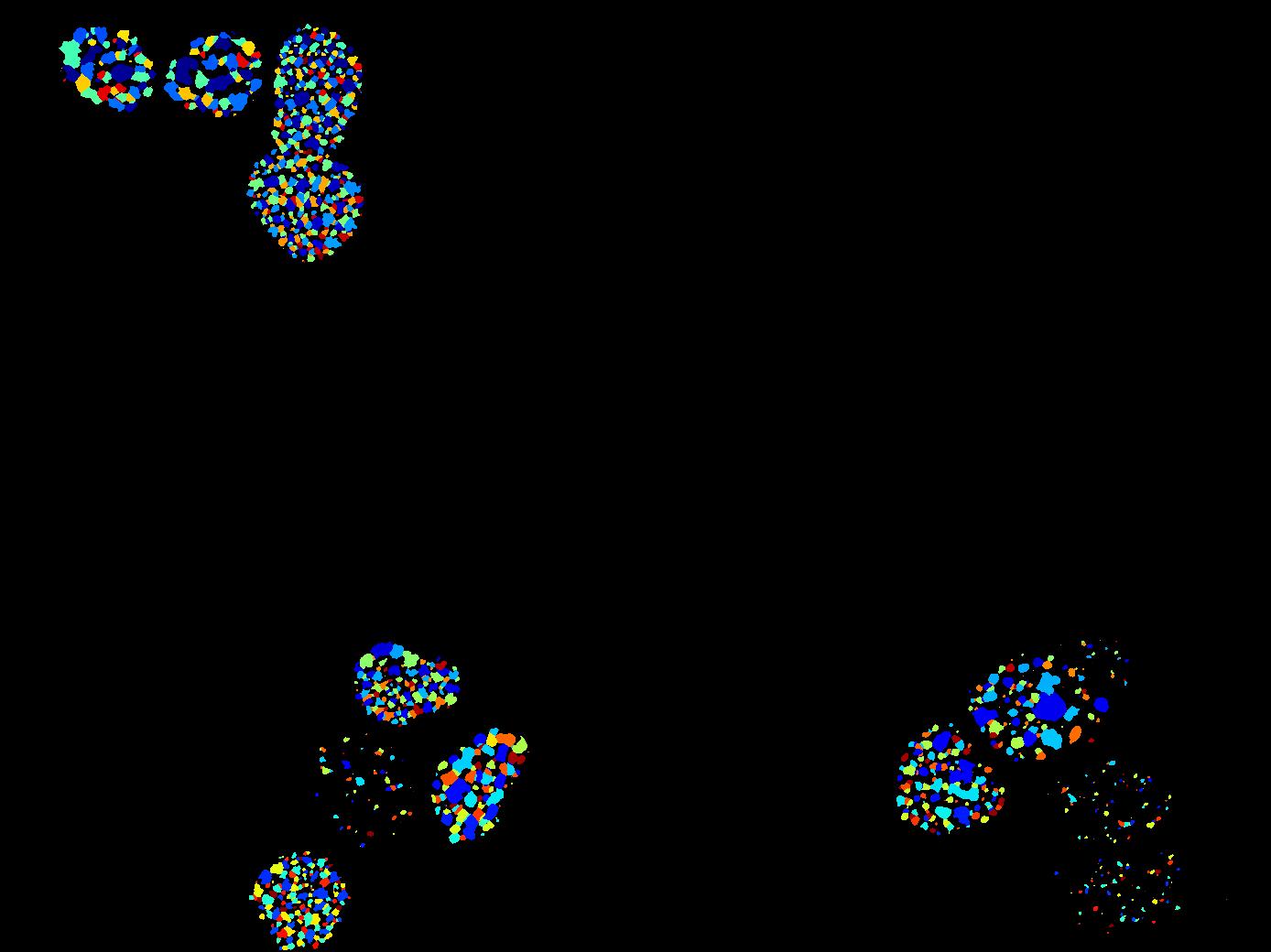

Supplement: Supplementary file 7 — Source Data [file 41467_2022_28822_MOESM7_ESM.zip › Figure 5E data/Masks/K136Q_I_02K136Q_48h 2_02_.jpeg]

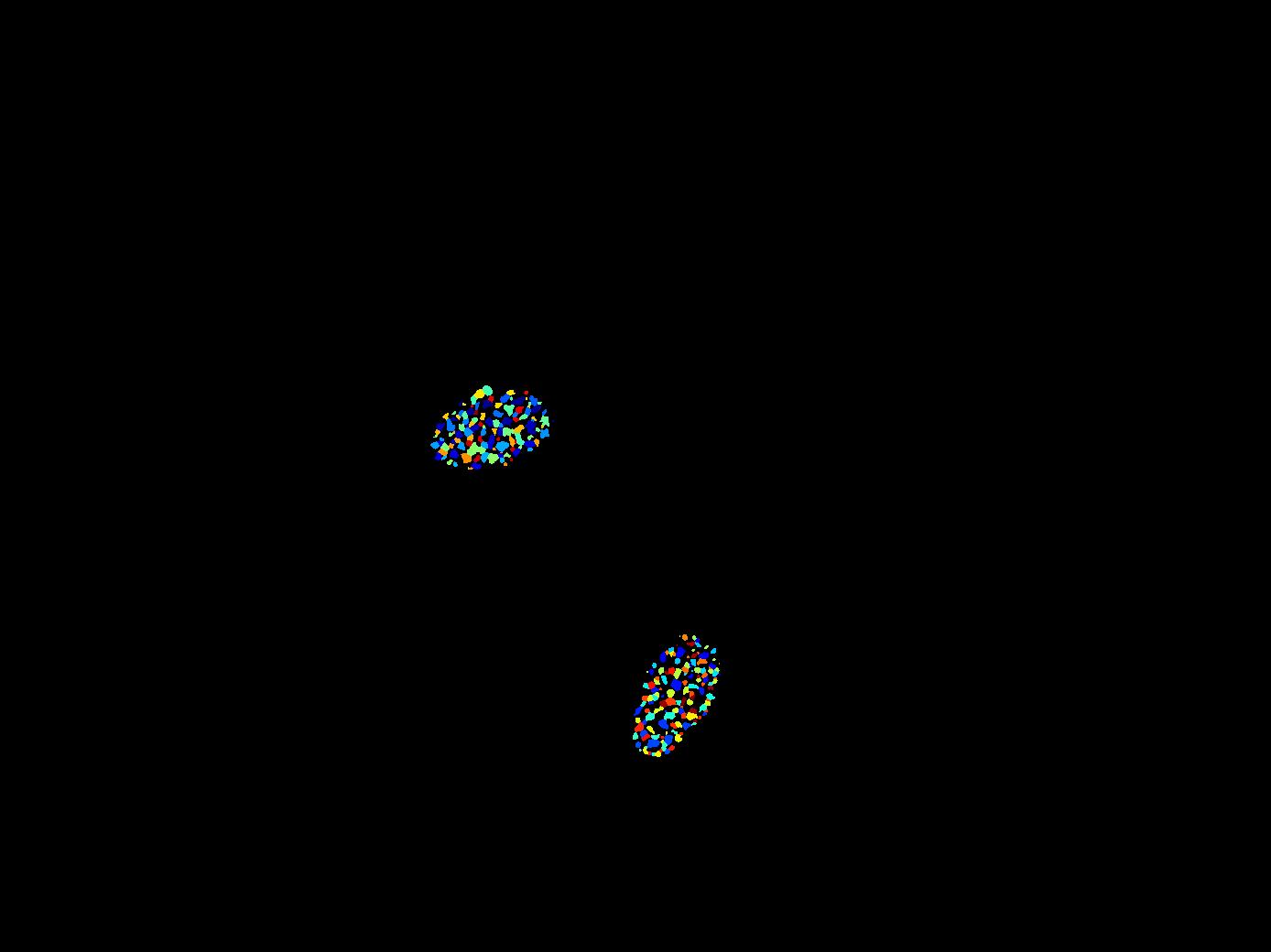

Supplement: Supplementary file 7 — Source Data [file 41467_2022_28822_MOESM7_ESM.zip › Figure 5E data/Masks/K136Q_I_02K136Q_48h_02_.jpeg]

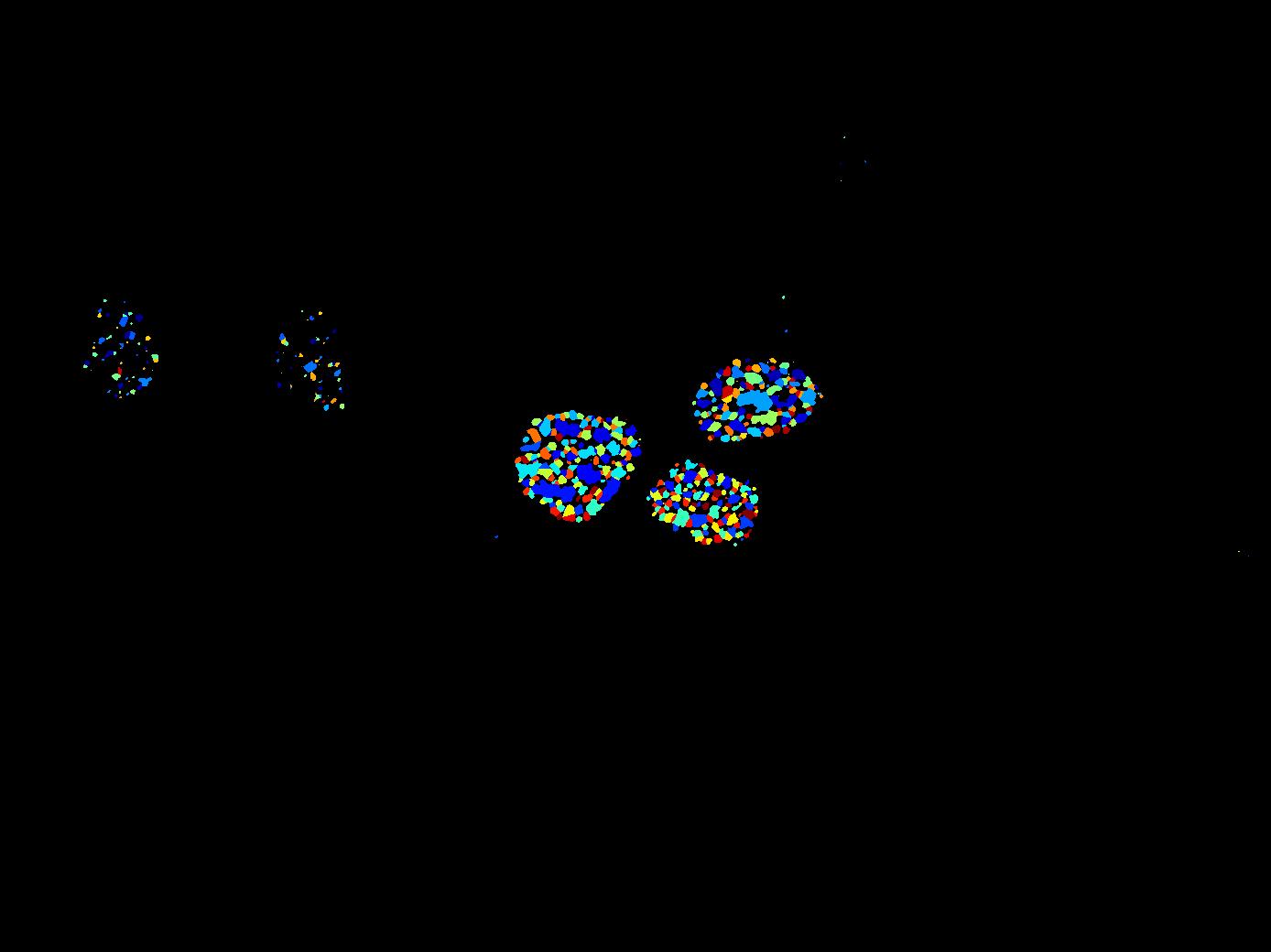

Supplement: Supplementary file 7 — Source Data [file 41467_2022_28822_MOESM7_ESM.zip › Figure 5E data/Masks/K136Q_I_02K136Q_72h 2_02_.jpeg]

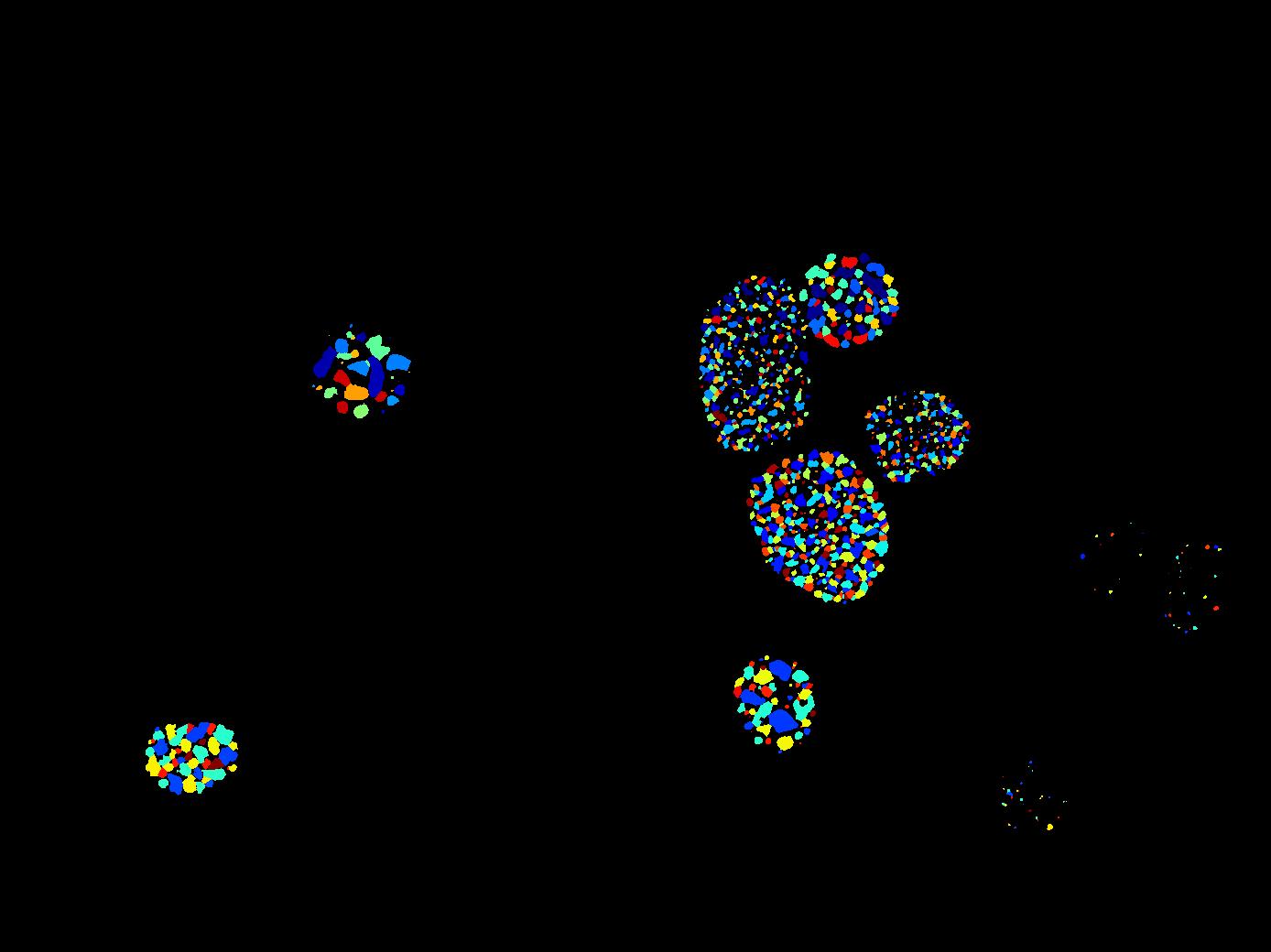

Supplement: Supplementary file 7 — Source Data [file 41467_2022_28822_MOESM7_ESM.zip › Figure 5E data/Masks/K136Q_I_02K136Q_72h_02_.jpeg]

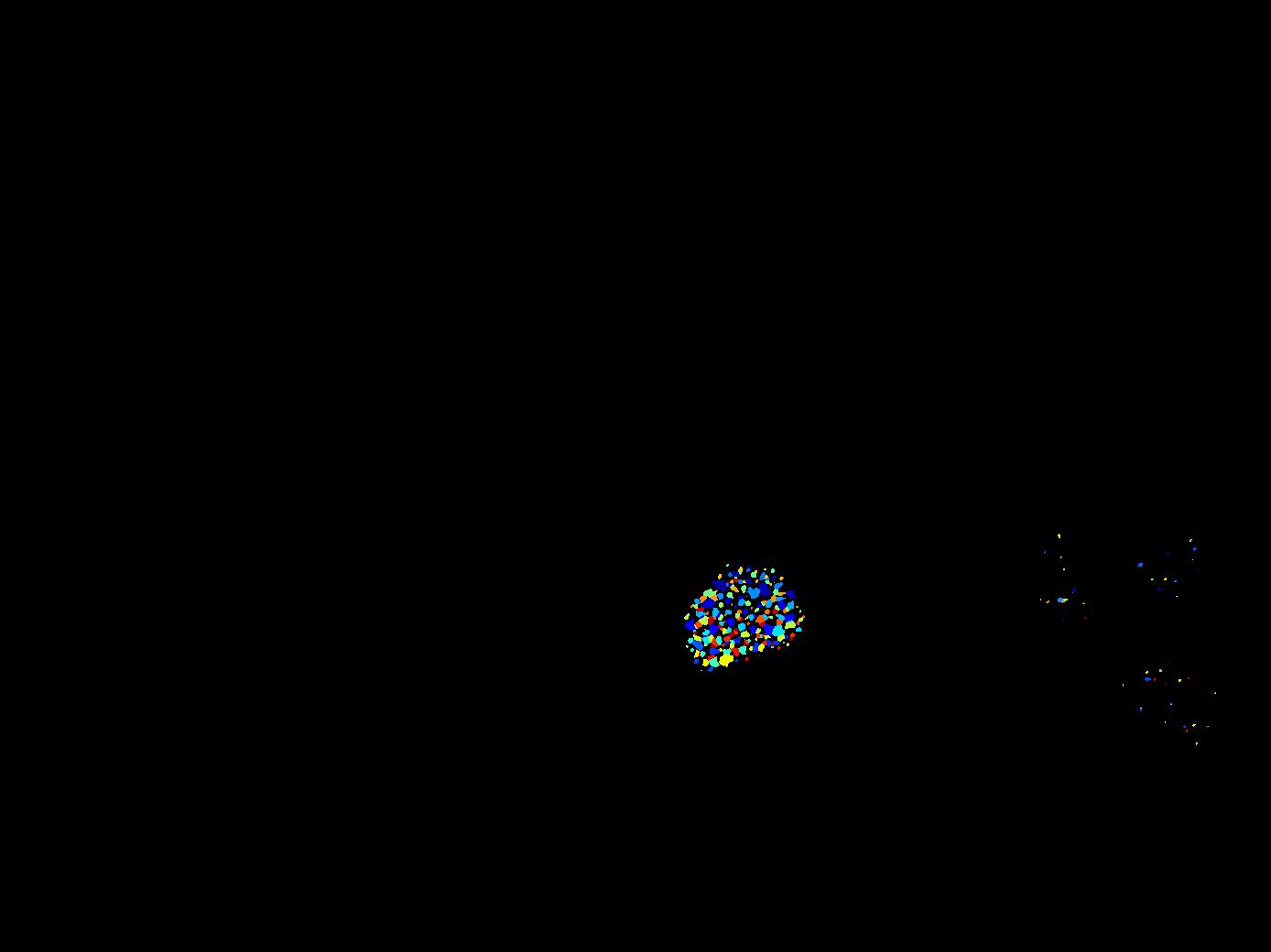

Supplement: Supplementary file 7 — Source Data [file 41467_2022_28822_MOESM7_ESM.zip › Figure 5E data/Masks/K136Q_I_03K136Q_24h 2_03_.jpeg]

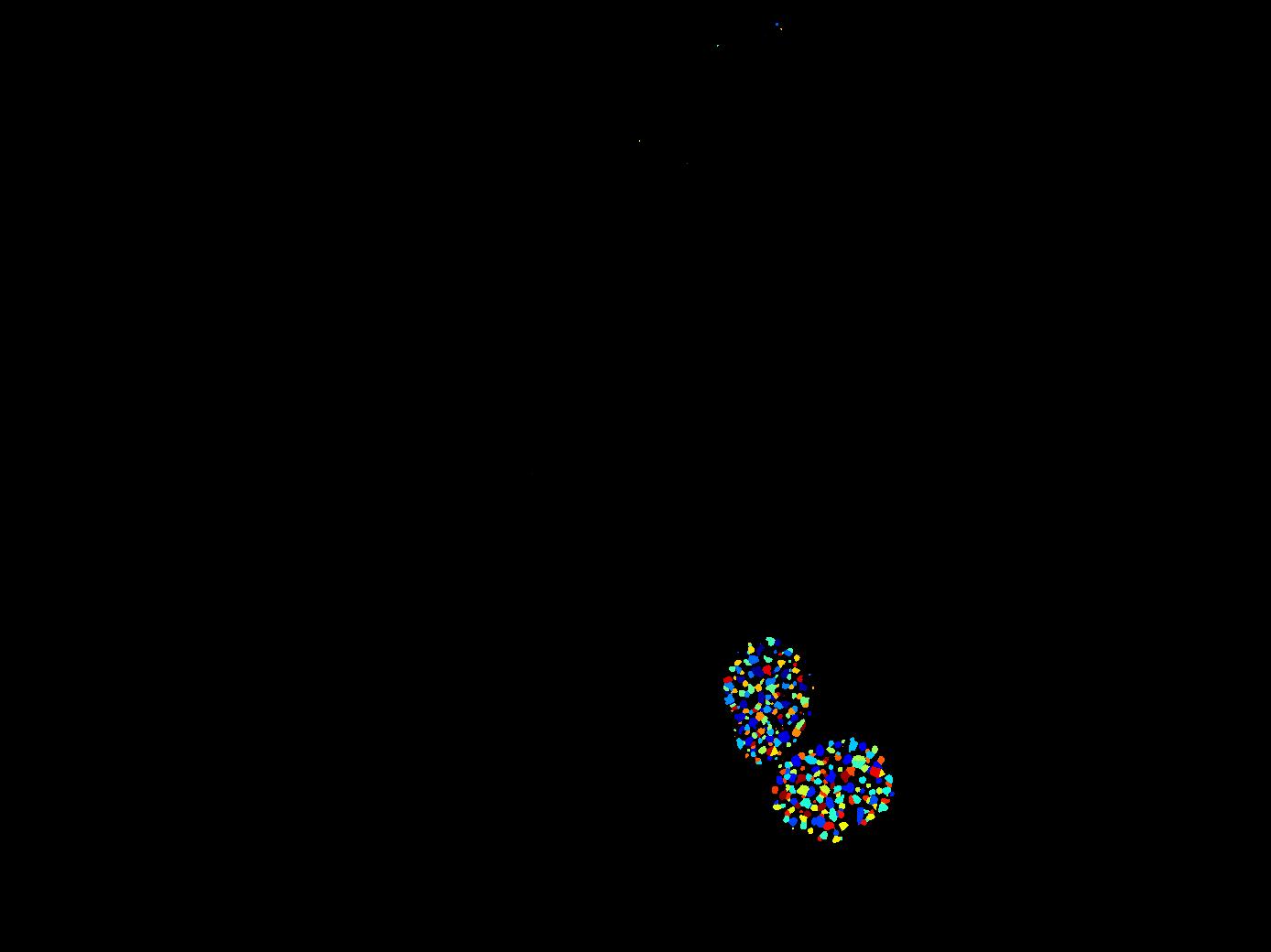

Supplement: Supplementary file 7 — Source Data [file 41467_2022_28822_MOESM7_ESM.zip › Figure 5E data/Masks/K136Q_I_03K136Q_24h_03_.jpeg]

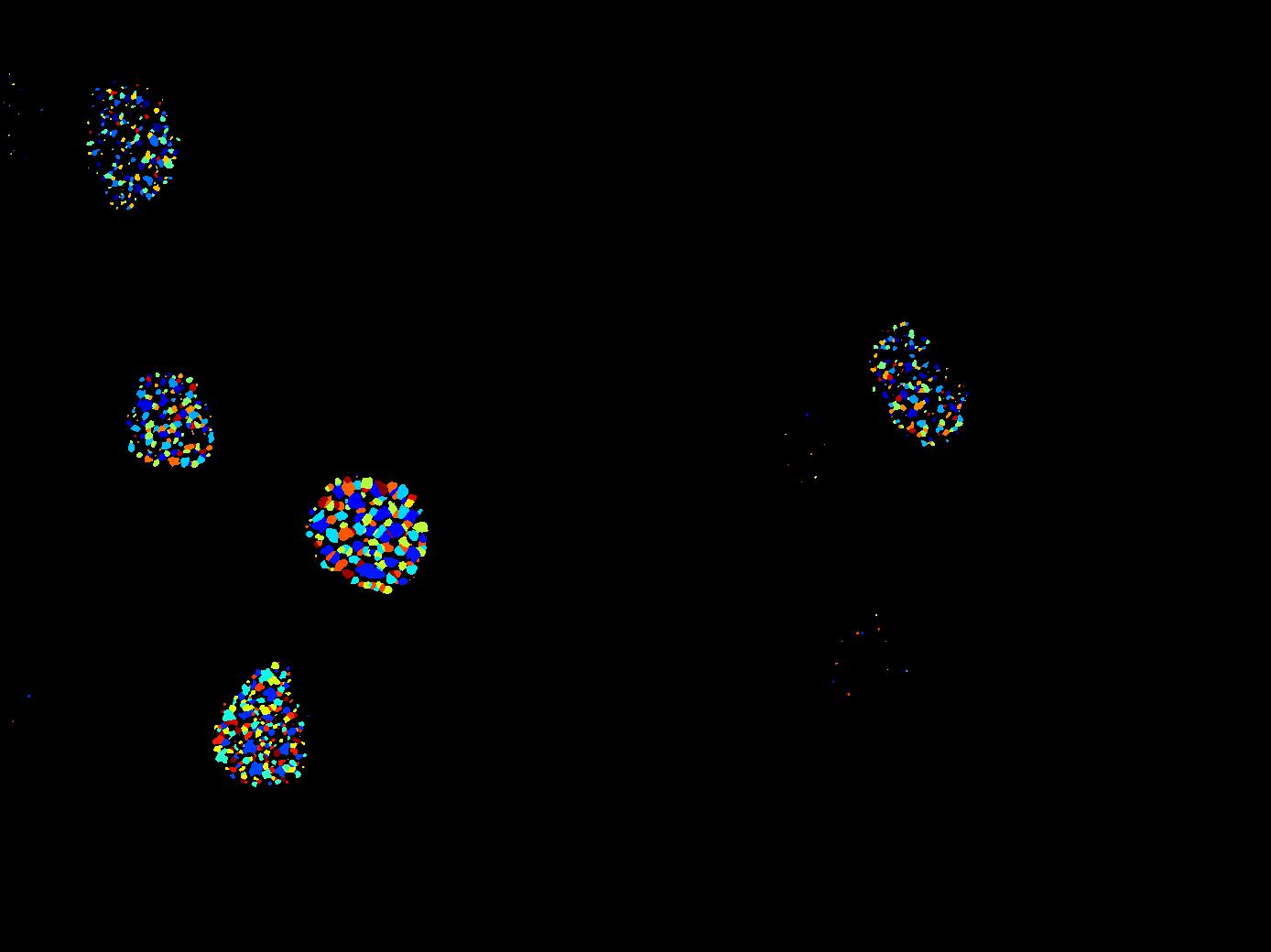

Supplement: Supplementary file 7 — Source Data [file 41467_2022_28822_MOESM7_ESM.zip › Figure 5E data/Masks/K136Q_I_03K136Q_48h 2_03_.jpeg]

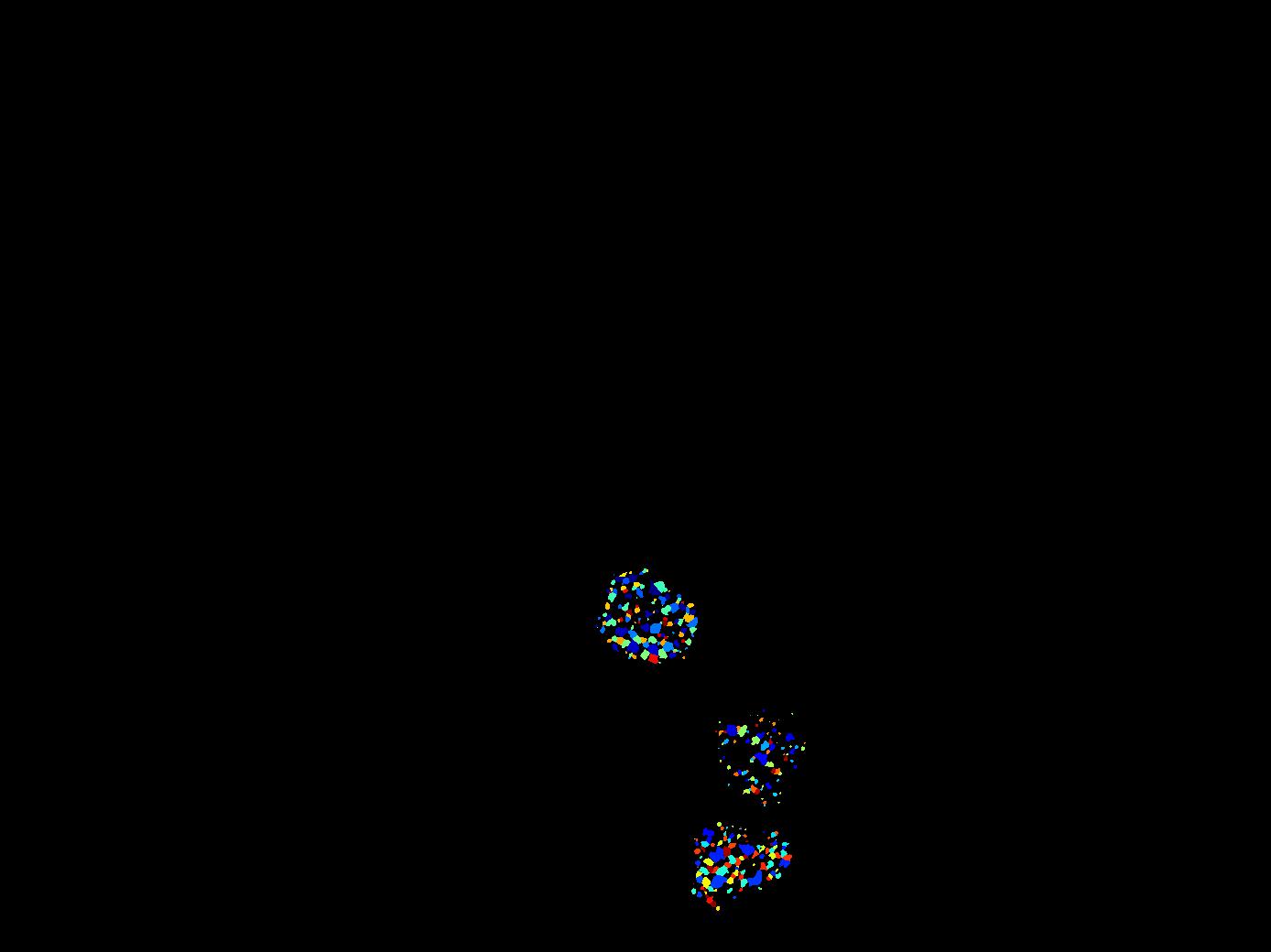

Supplement: Supplementary file 7 — Source Data [file 41467_2022_28822_MOESM7_ESM.zip › Figure 5E data/Masks/K136Q_I_03K136Q_48h_03_.jpeg]

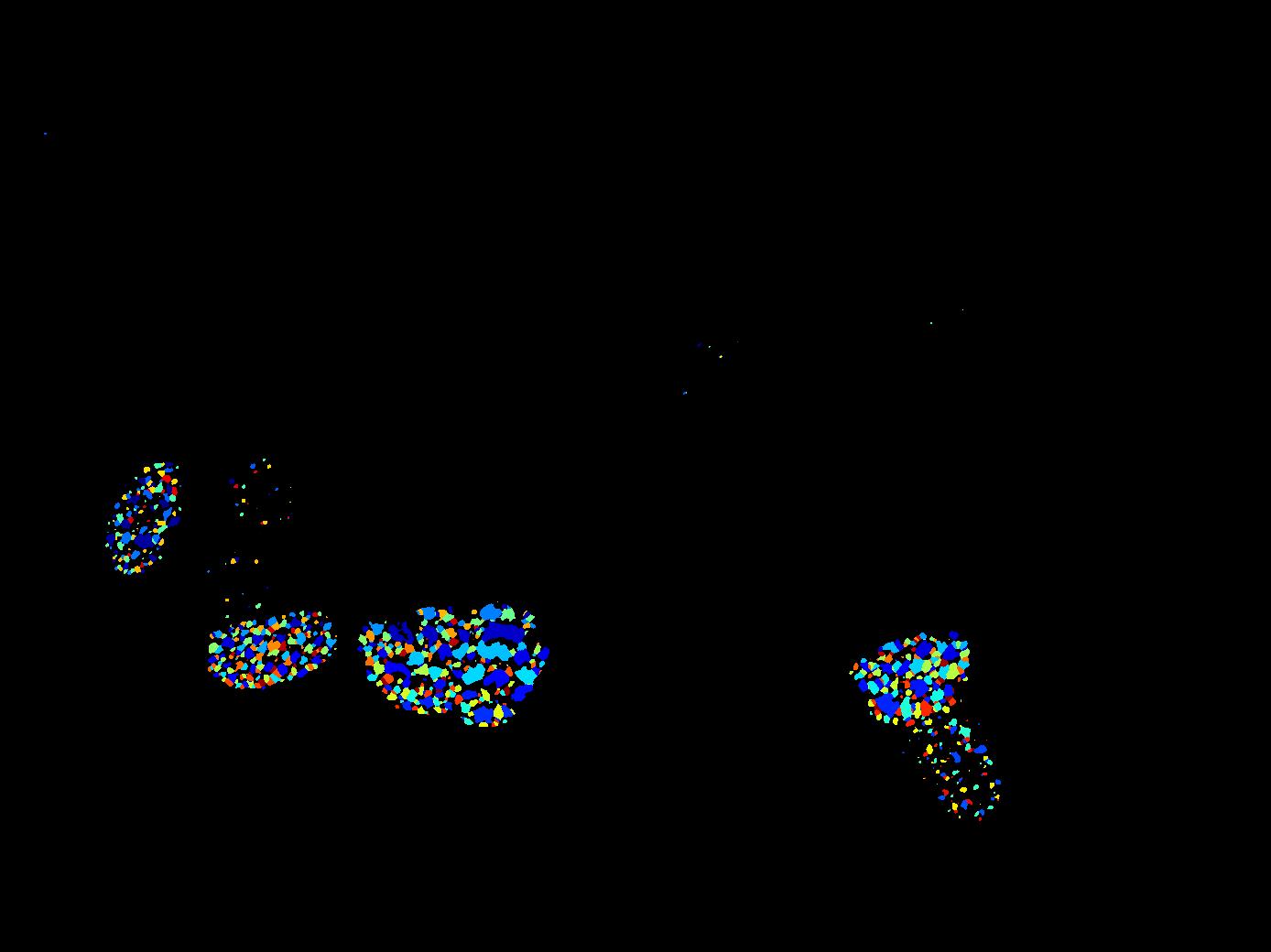

Supplement: Supplementary file 7 — Source Data [file 41467_2022_28822_MOESM7_ESM.zip › Figure 5E data/Masks/K136Q_I_03K136Q_72h 2_03_.jpeg]

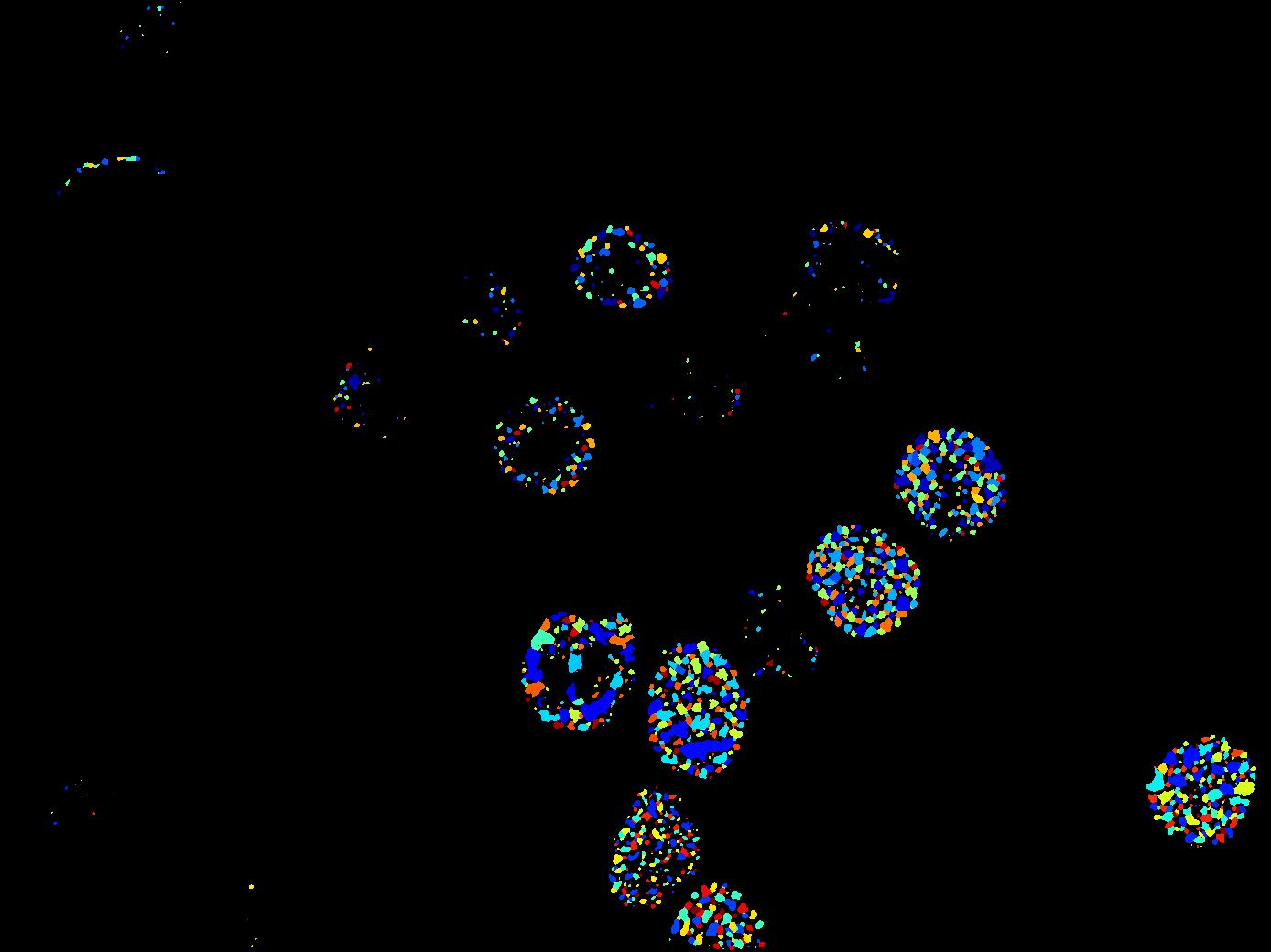

Supplement: Supplementary file 7 — Source Data [file 41467_2022_28822_MOESM7_ESM.zip › Figure 5E data/Masks/K136Q_I_03K136Q_72h_03_.jpeg]

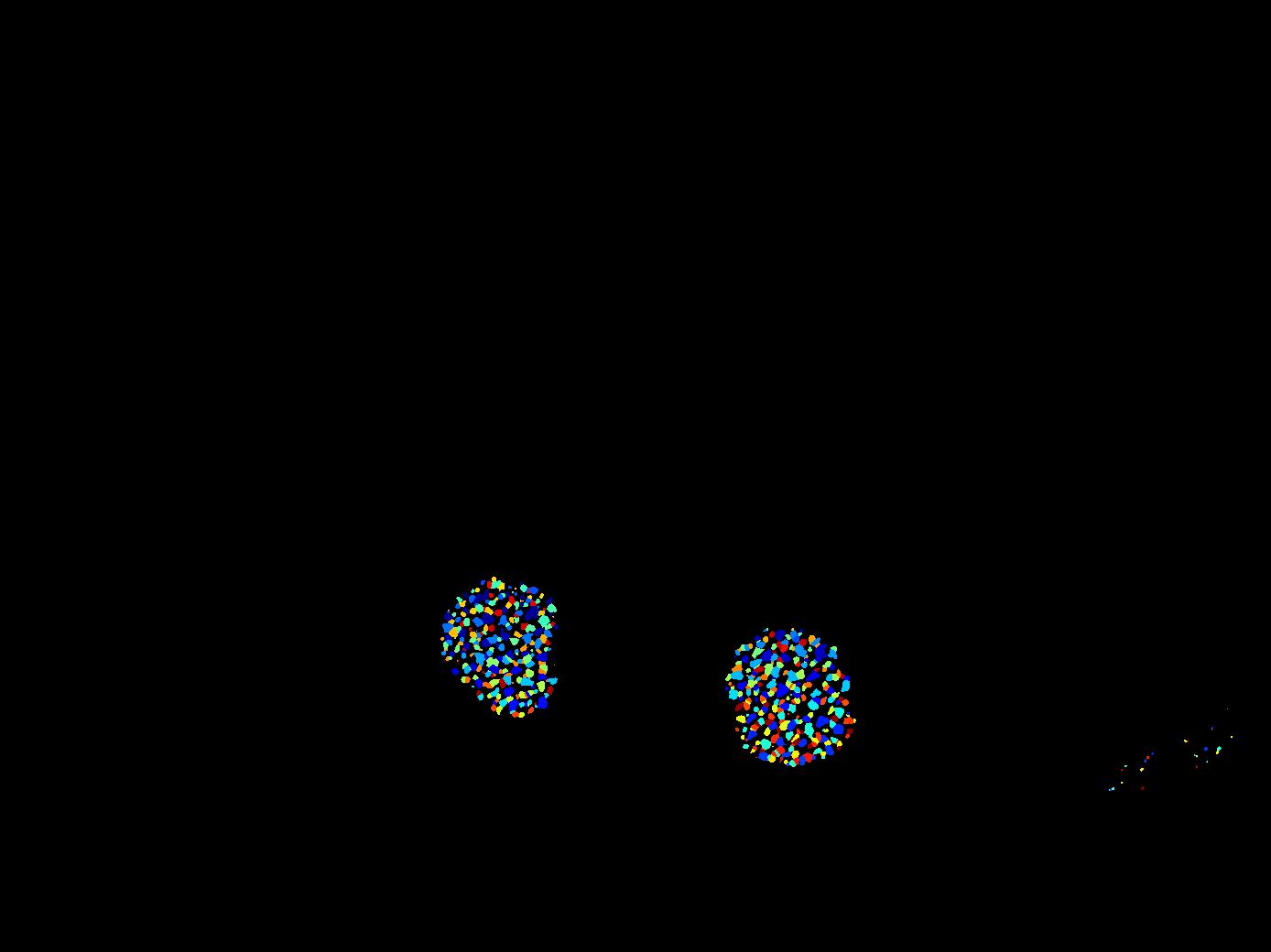

Supplement: Supplementary file 7 — Source Data [file 41467_2022_28822_MOESM7_ESM.zip › Figure 5E data/Masks/K136Q_I_04K136Q_24h 2_04_.jpeg]

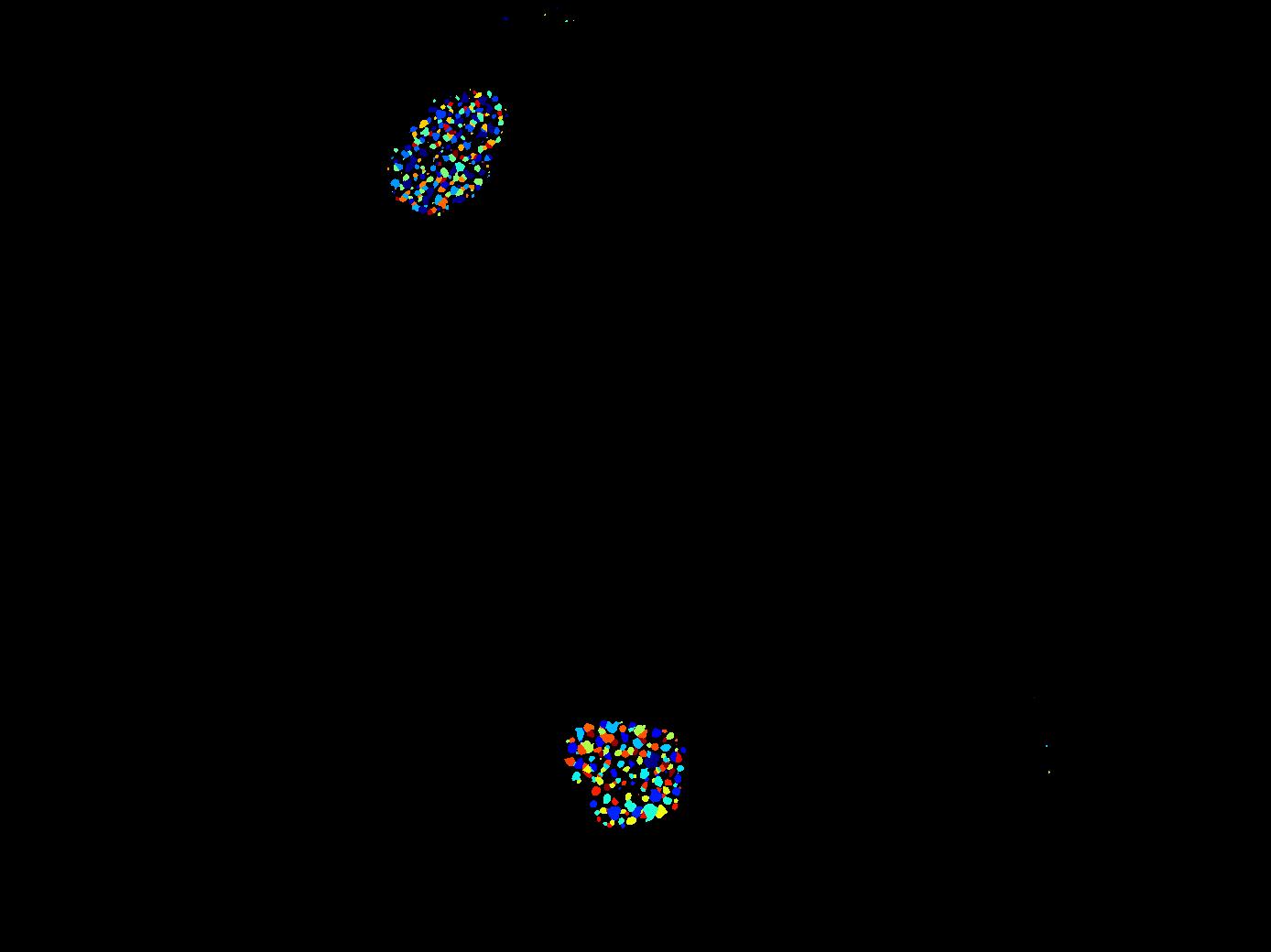

Supplement: Supplementary file 7 — Source Data [file 41467_2022_28822_MOESM7_ESM.zip › Figure 5E data/Masks/K136Q_I_04K136Q_24h_04_.jpeg]

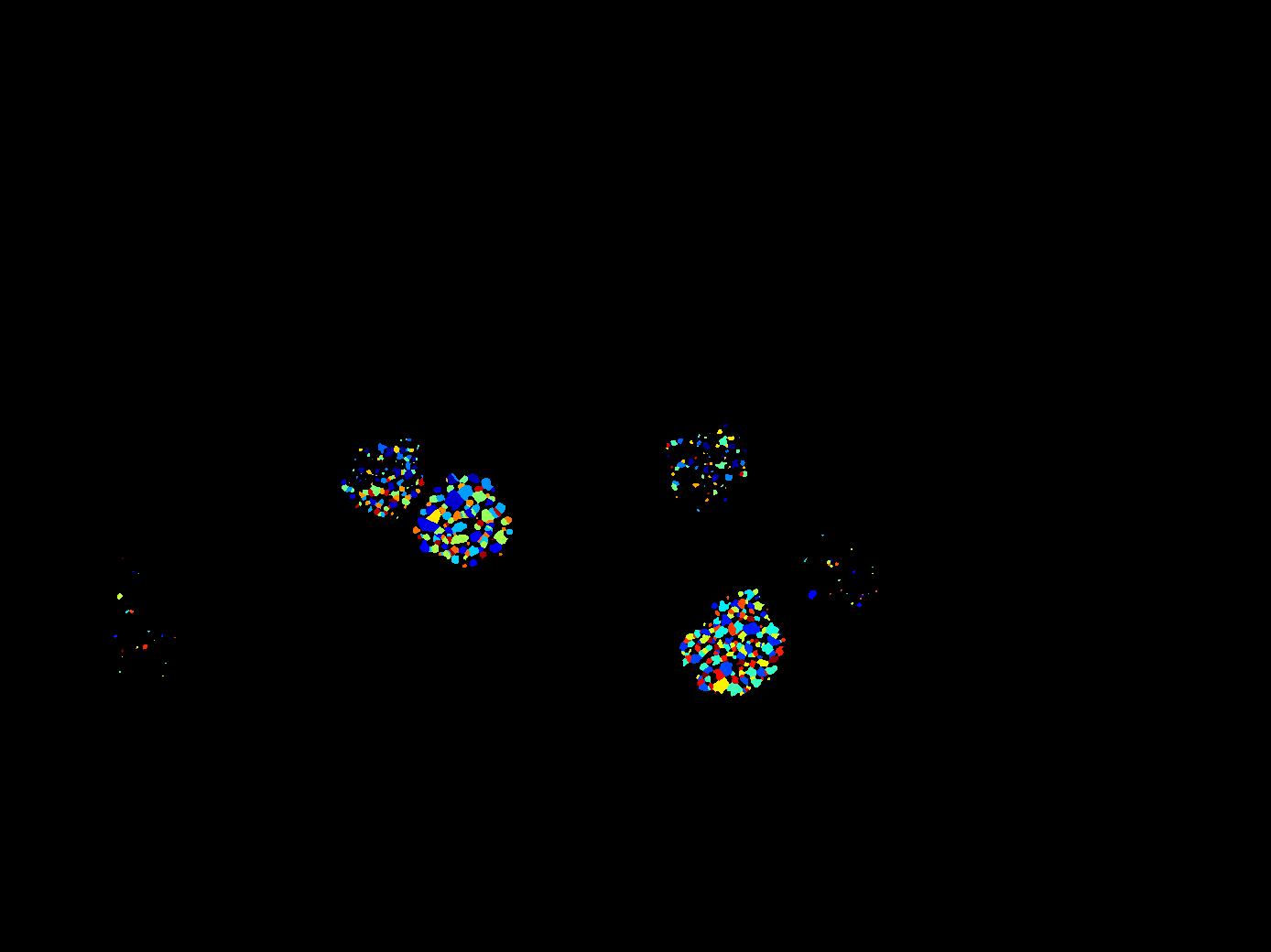

Supplement: Supplementary file 7 — Source Data [file 41467_2022_28822_MOESM7_ESM.zip › Figure 5E data/Masks/K136Q_I_04K136Q_48h 2_04_.jpeg]

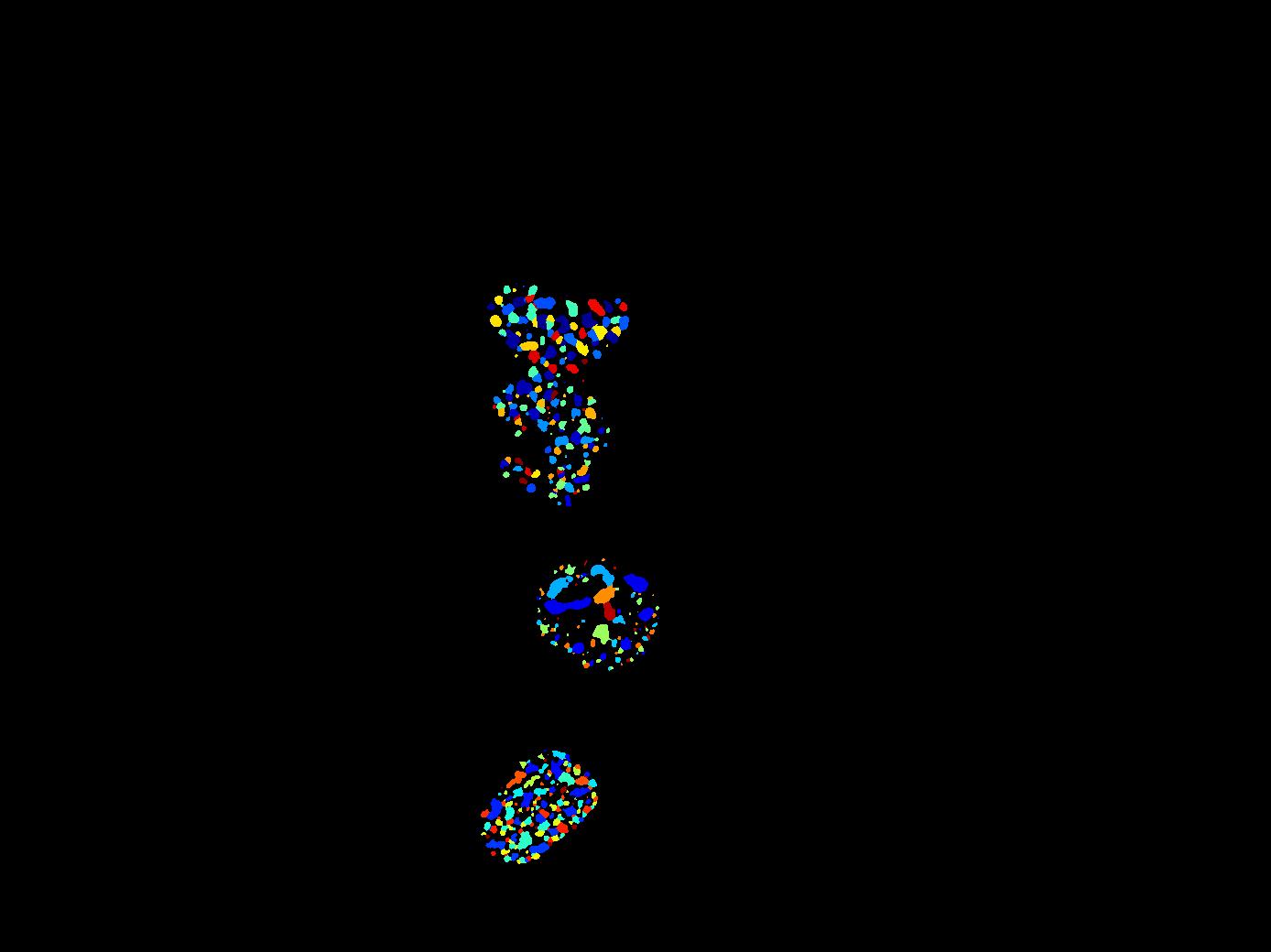

Supplement: Supplementary file 7 — Source Data [file 41467_2022_28822_MOESM7_ESM.zip › Figure 5E data/Masks/K136Q_I_04K136Q_48h_04_.jpeg]

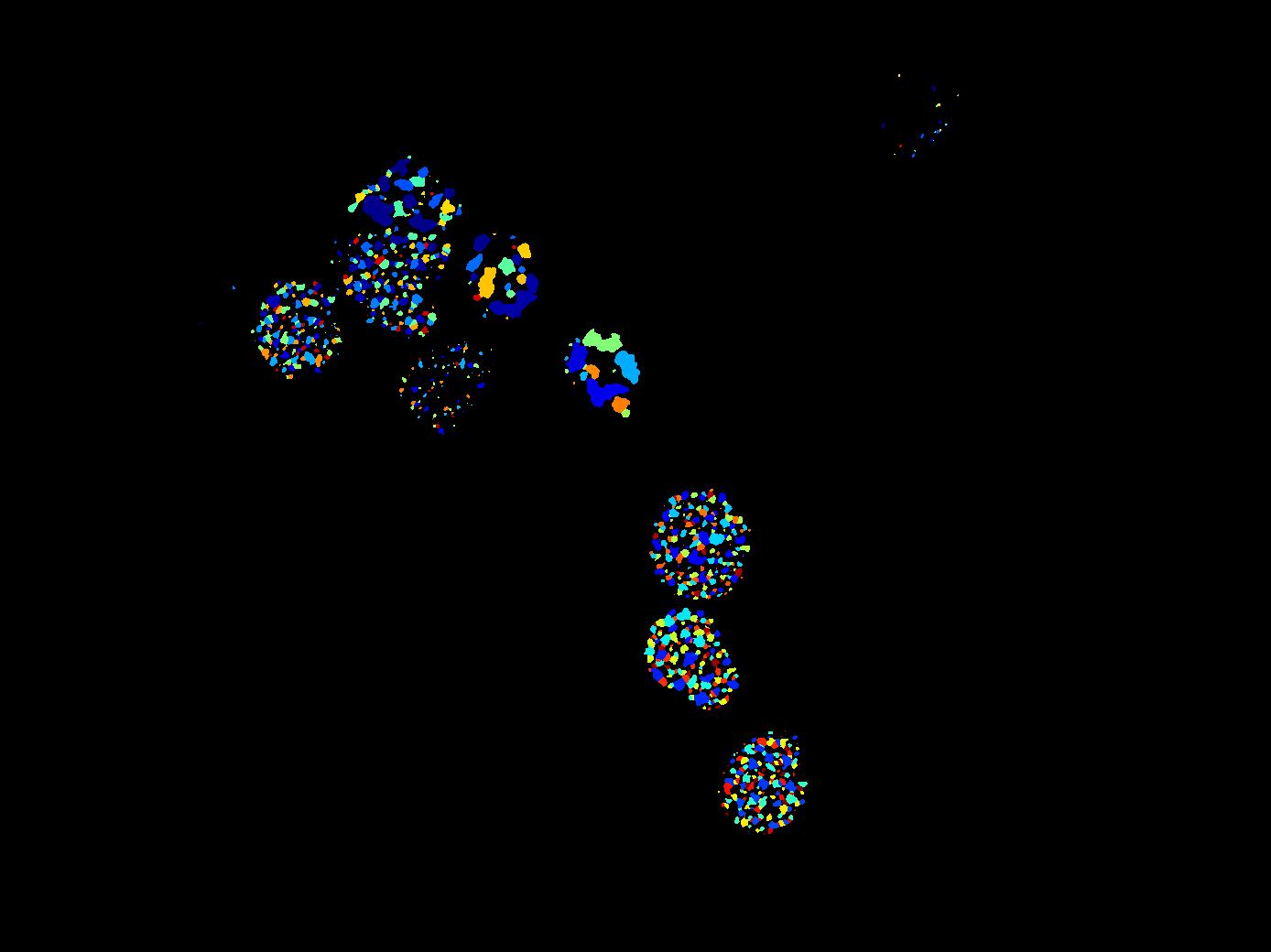

Supplement: Supplementary file 7 — Source Data [file 41467_2022_28822_MOESM7_ESM.zip › Figure 5E data/Masks/K136Q_I_04K136Q_72h 2_04_.jpeg]

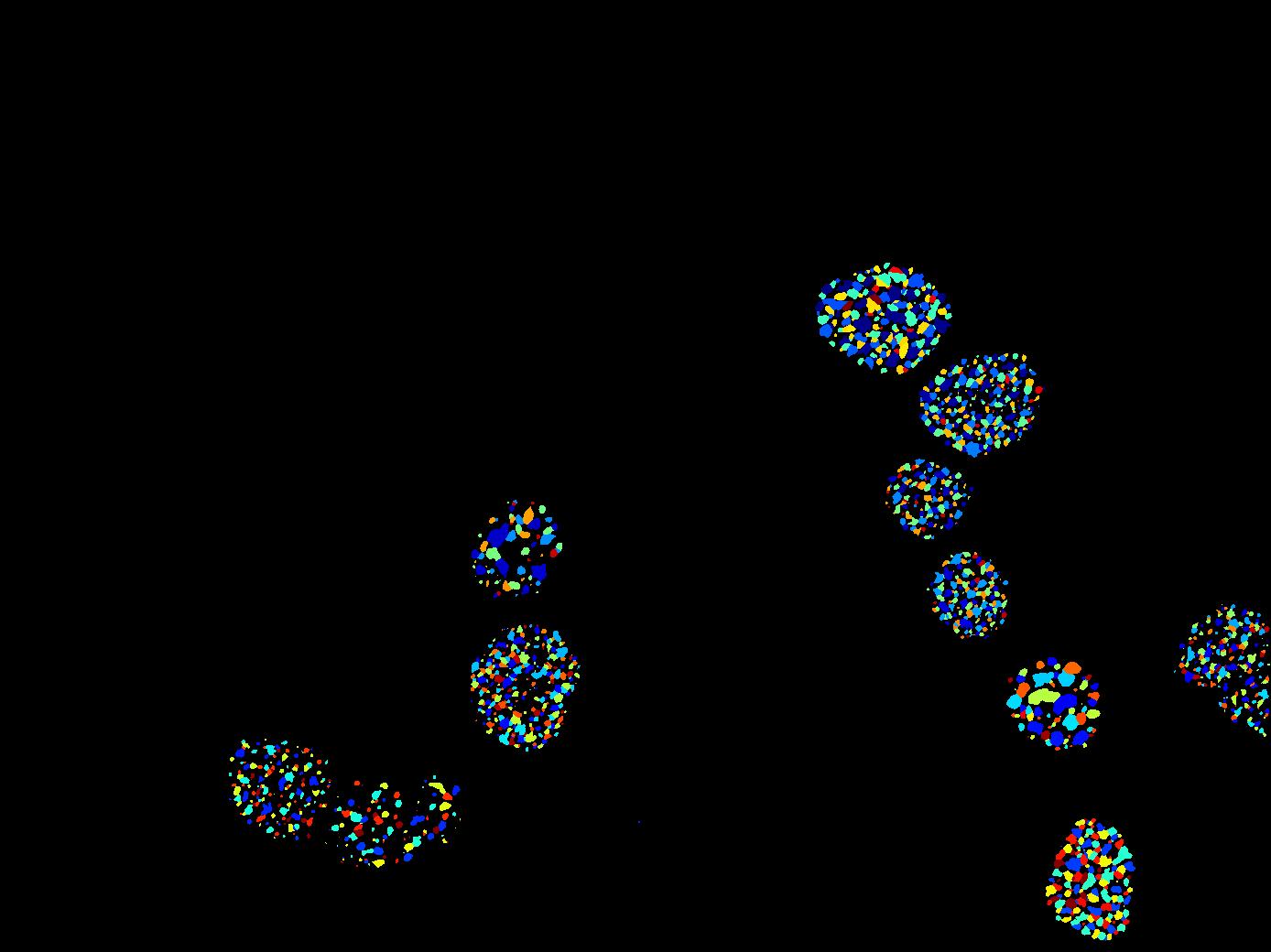

Supplement: Supplementary file 7 — Source Data [file 41467_2022_28822_MOESM7_ESM.zip › Figure 5E data/Masks/K136Q_I_04K136Q_72h_04_.jpeg]

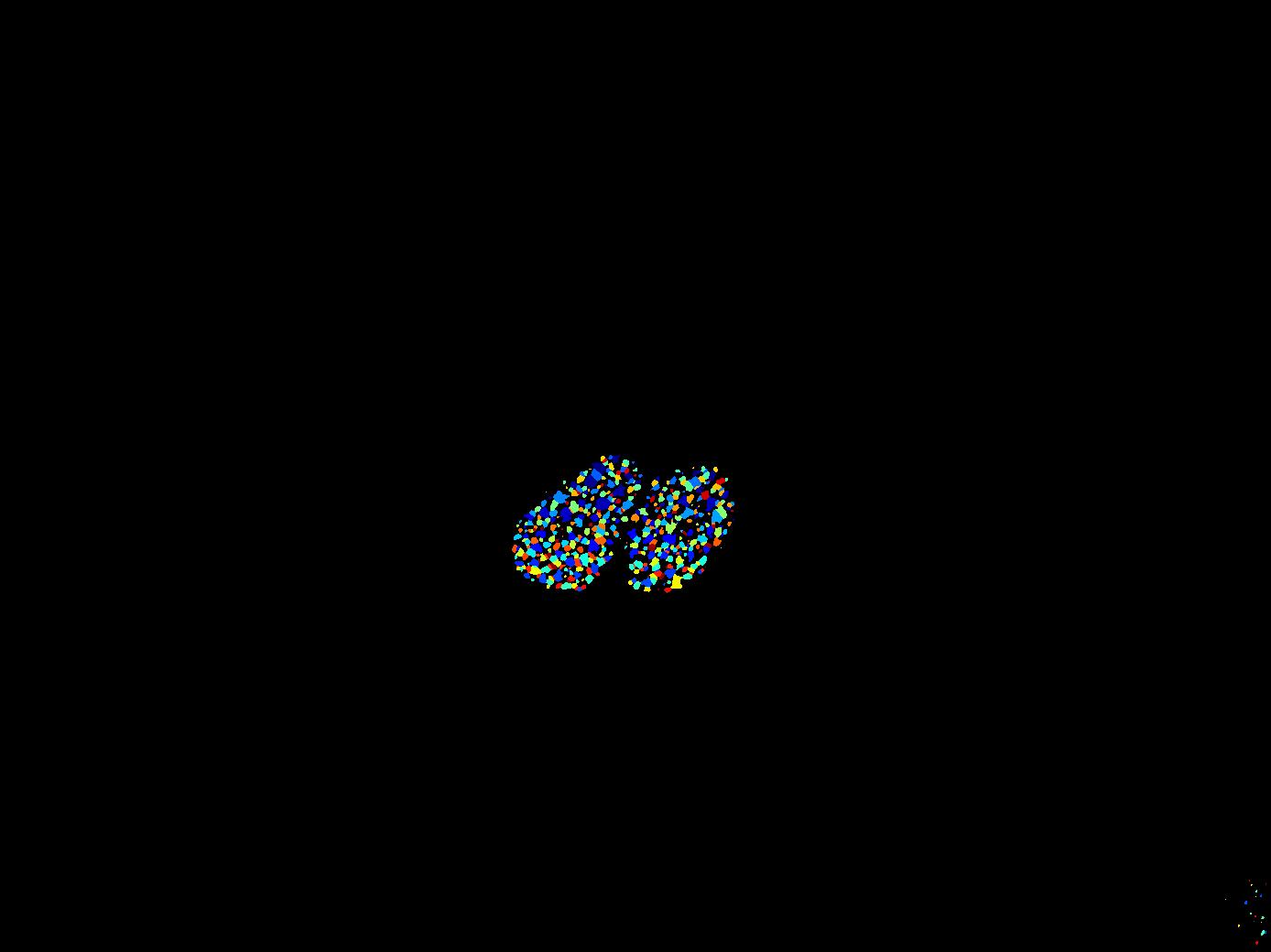

Supplement: Supplementary file 7 — Source Data [file 41467_2022_28822_MOESM7_ESM.zip › Figure 5E data/Masks/K136Q_I_05K136Q_24h 2_05_.jpeg]

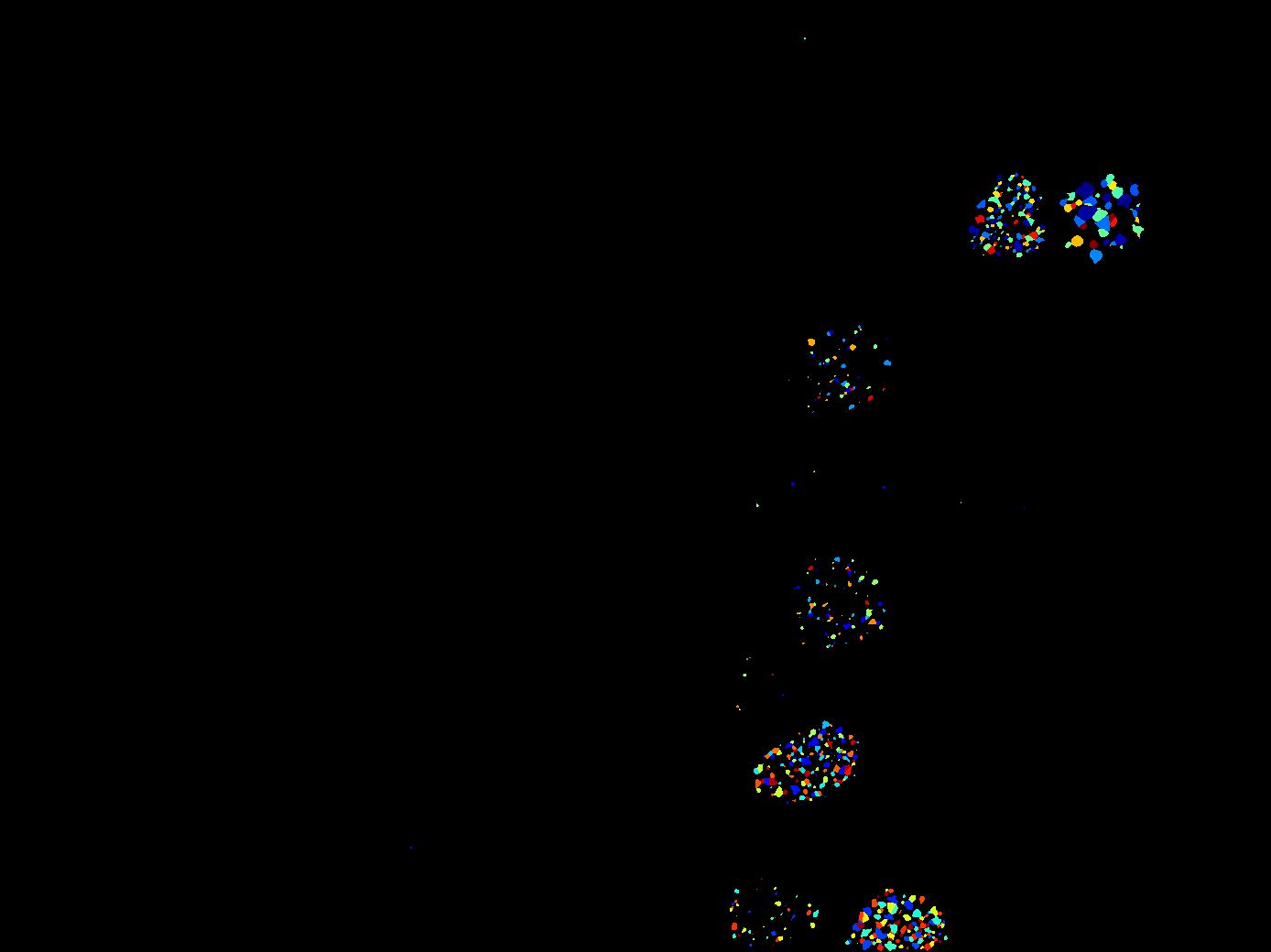

Supplement: Supplementary file 7 — Source Data [file 41467_2022_28822_MOESM7_ESM.zip › Figure 5E data/Masks/K136Q_I_05K136Q_24h_05_.jpeg]

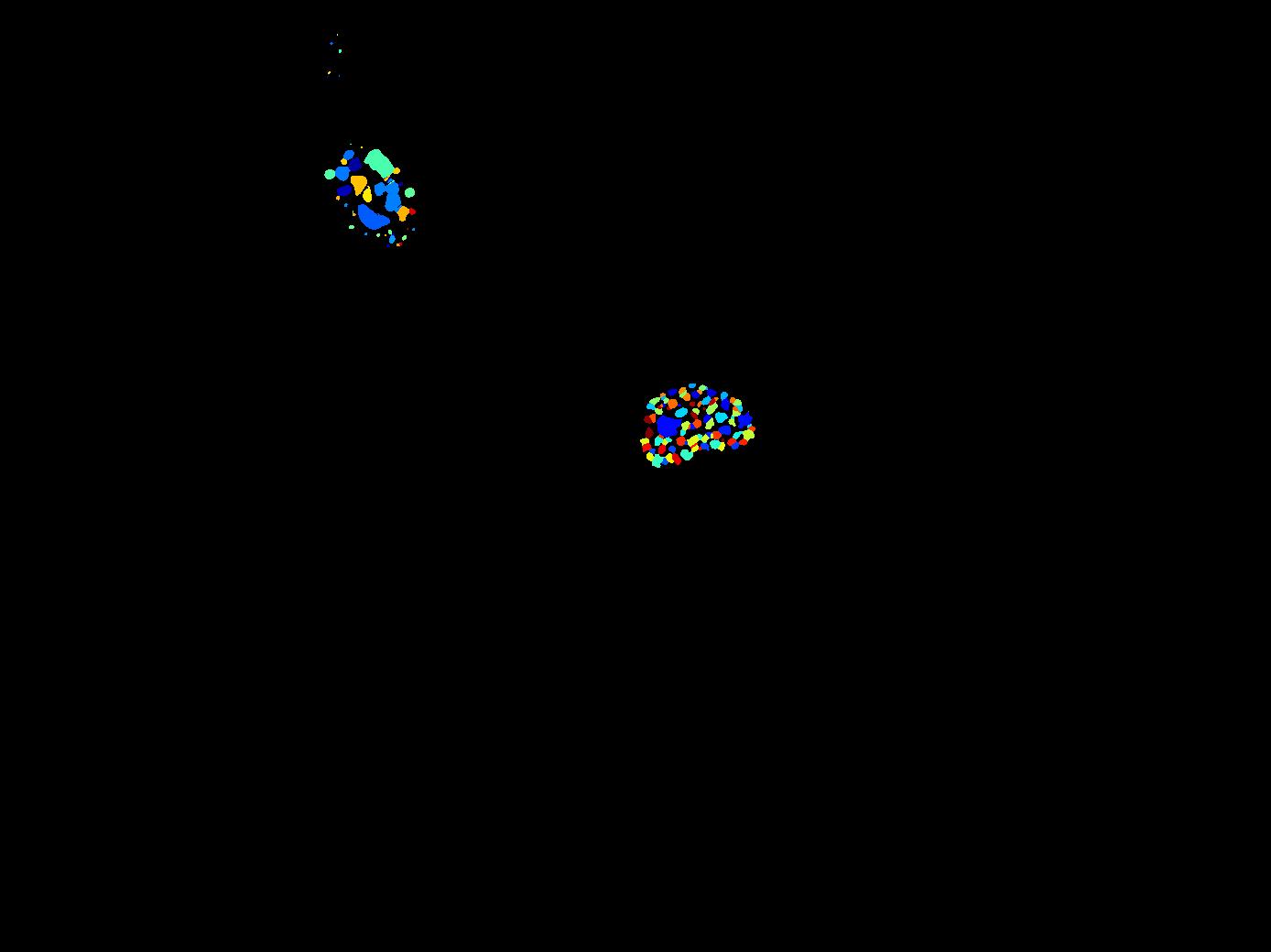

Supplement: Supplementary file 7 — Source Data [file 41467_2022_28822_MOESM7_ESM.zip › Figure 5E data/Masks/K136Q_I_05K136Q_48h 2_05_.jpeg]

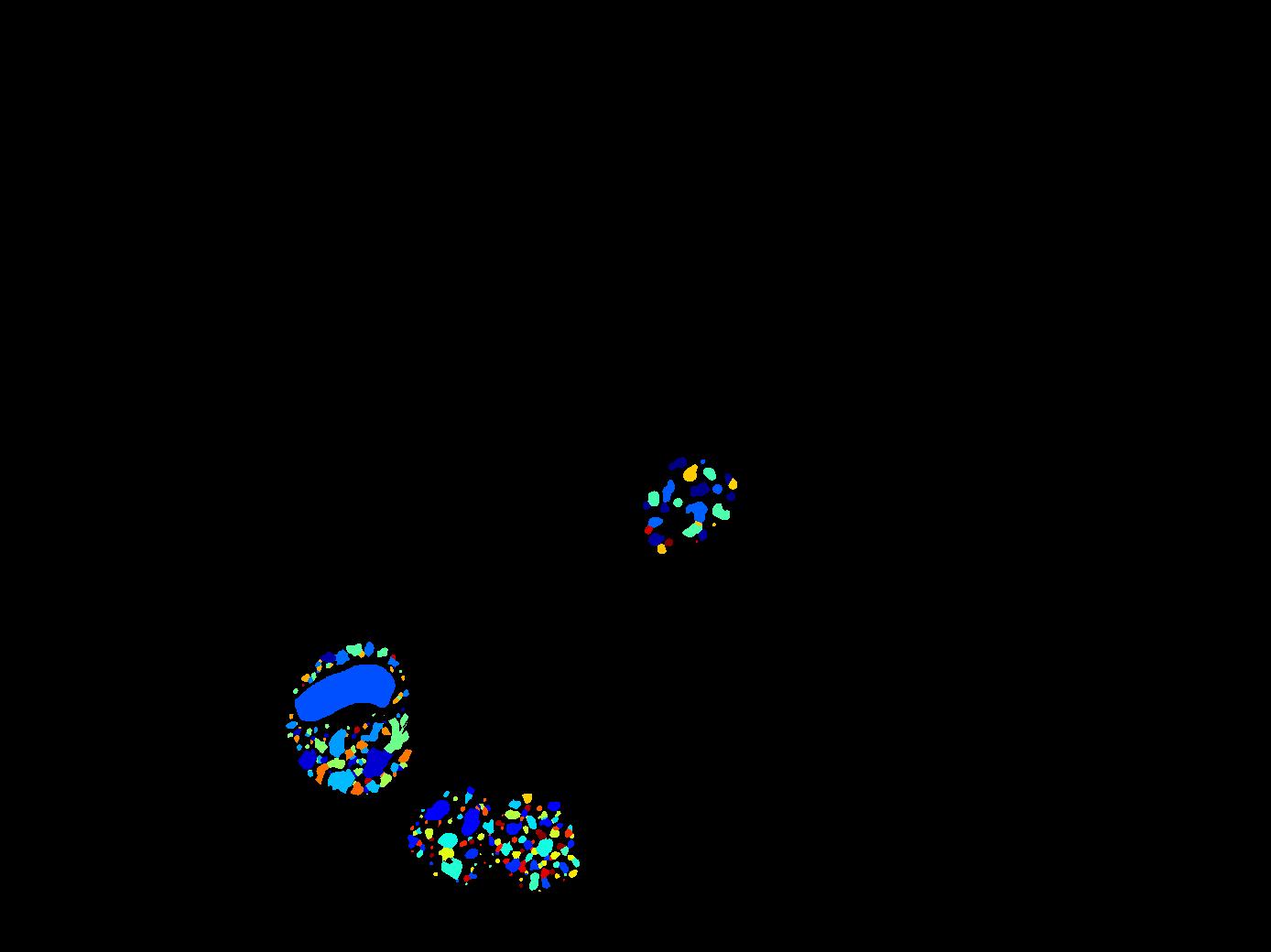

Supplement: Supplementary file 7 — Source Data [file 41467_2022_28822_MOESM7_ESM.zip › Figure 5E data/Masks/K136Q_I_05K136Q_48h_05_.jpeg]

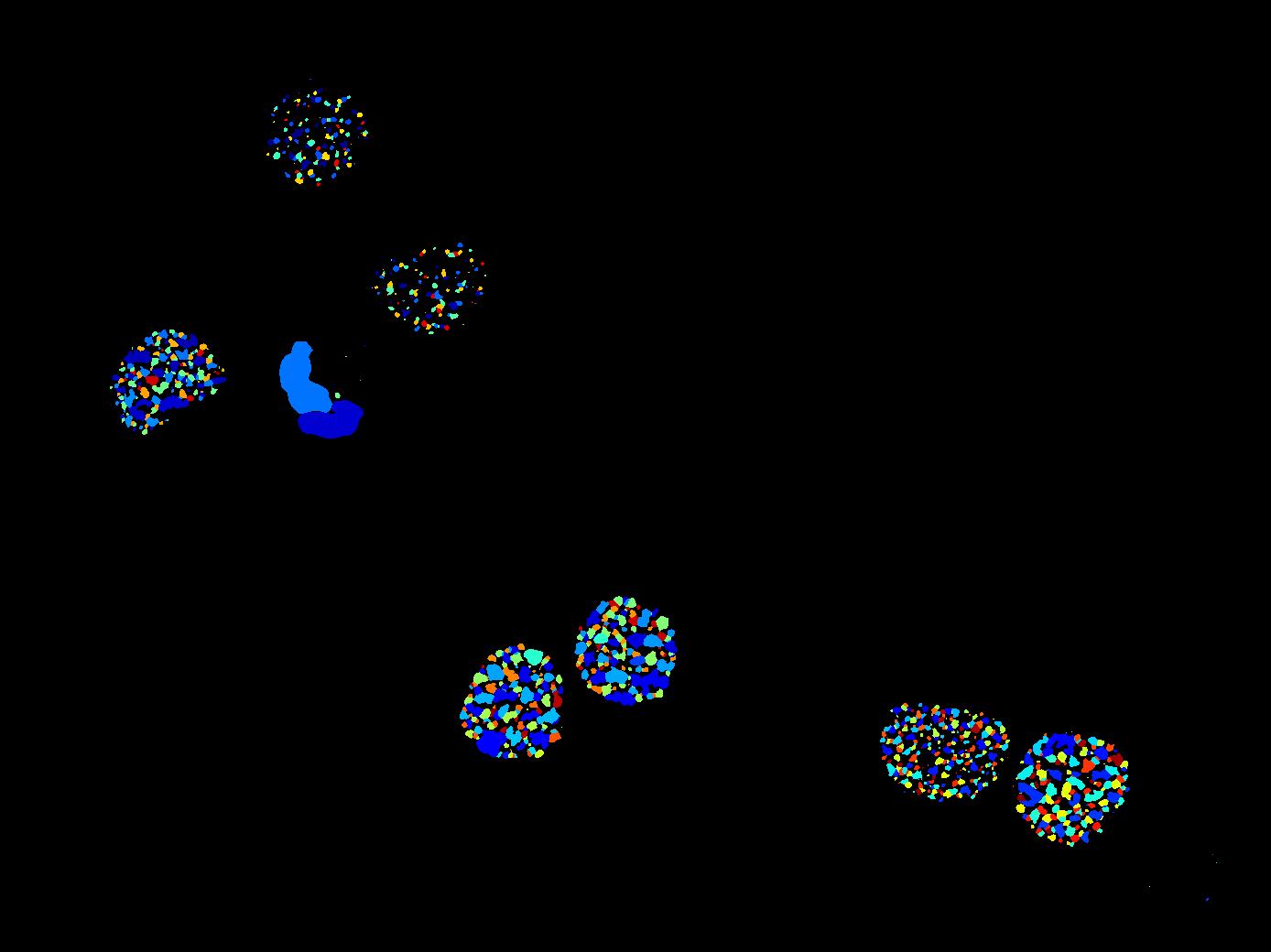

Supplement: Supplementary file 7 — Source Data [file 41467_2022_28822_MOESM7_ESM.zip › Figure 5E data/Masks/K136Q_I_05K136Q_72h 2_05_.jpeg]

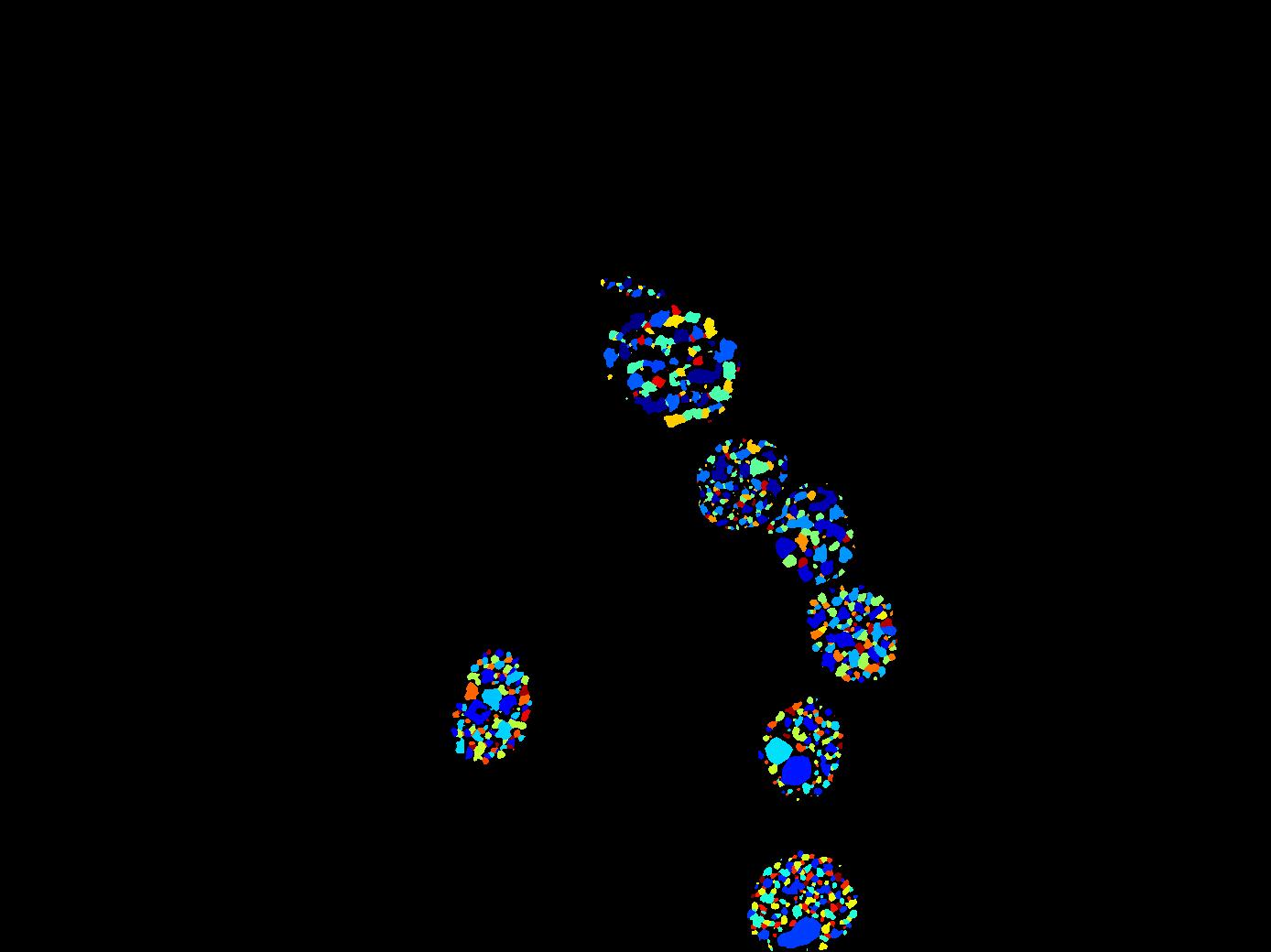

Supplement: Supplementary file 7 — Source Data [file 41467_2022_28822_MOESM7_ESM.zip › Figure 5E data/Masks/K136Q_I_05K136Q_72h_05_.jpeg]

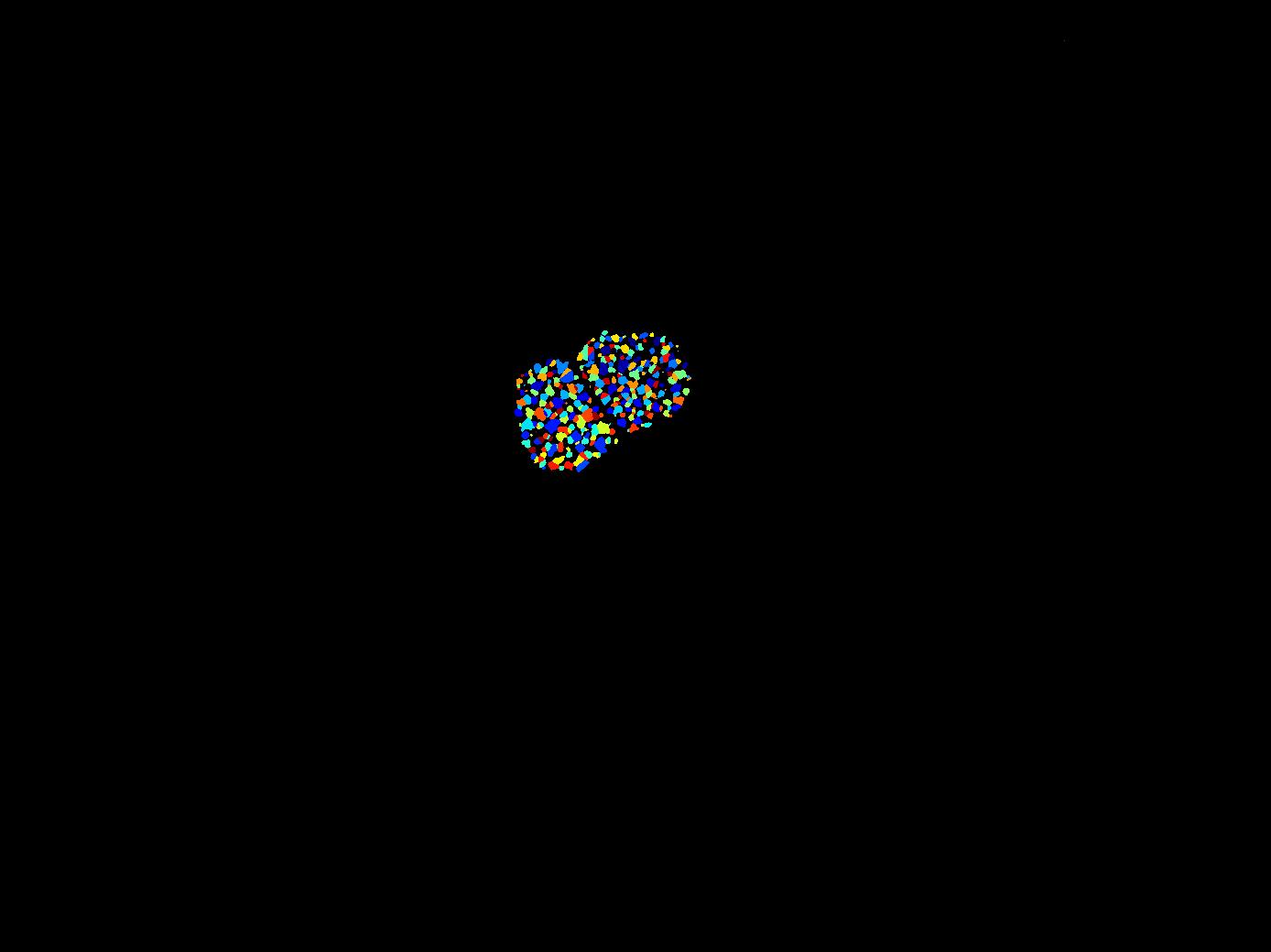

Supplement: Supplementary file 7 — Source Data [file 41467_2022_28822_MOESM7_ESM.zip › Figure 5E data/Masks/K136Q_I_06K136Q_24h 2_06_.jpeg]

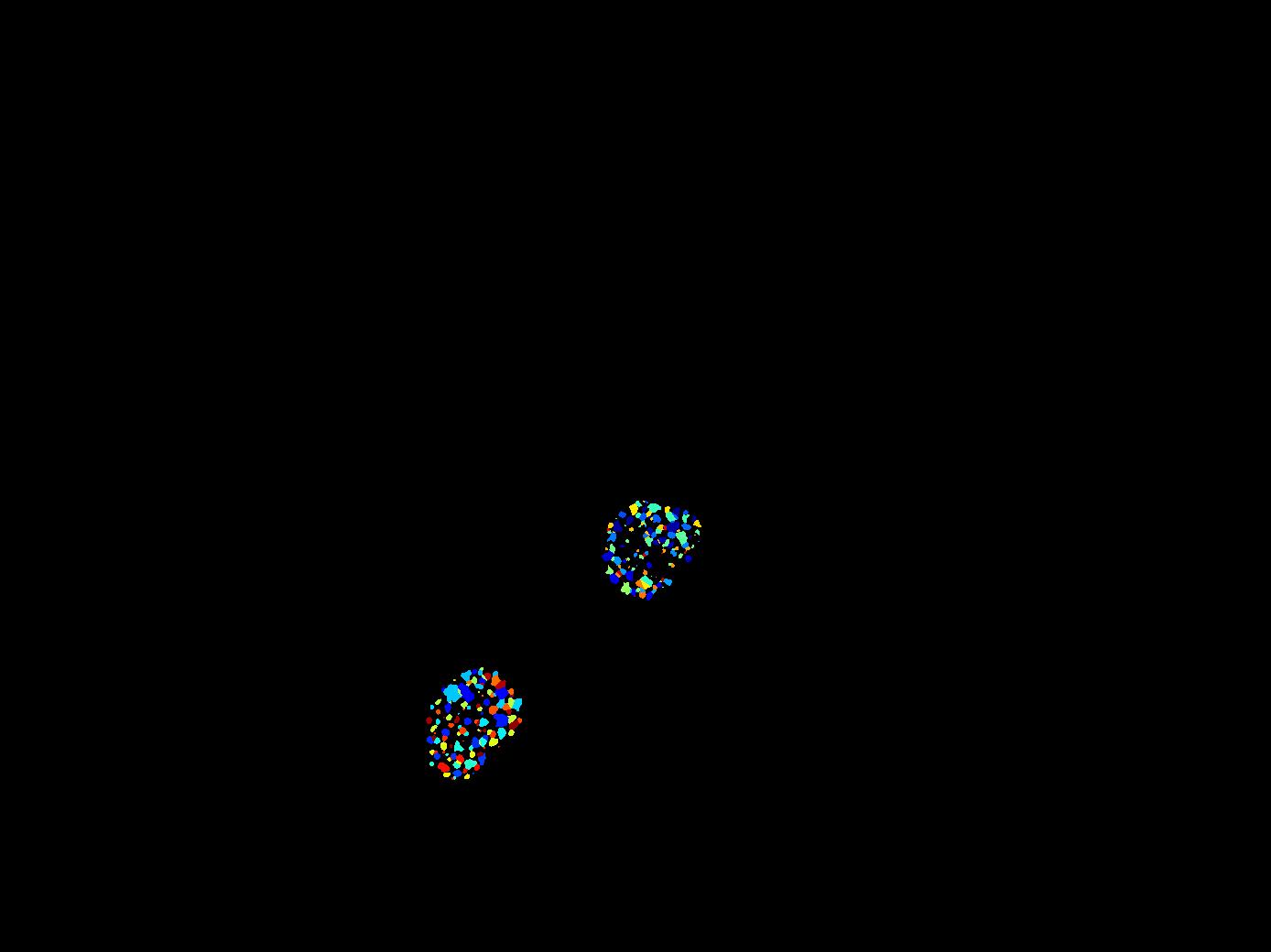

Supplement: Supplementary file 7 — Source Data [file 41467_2022_28822_MOESM7_ESM.zip › Figure 5E data/Masks/K136Q_I_06K136Q_24h_06_.jpeg]

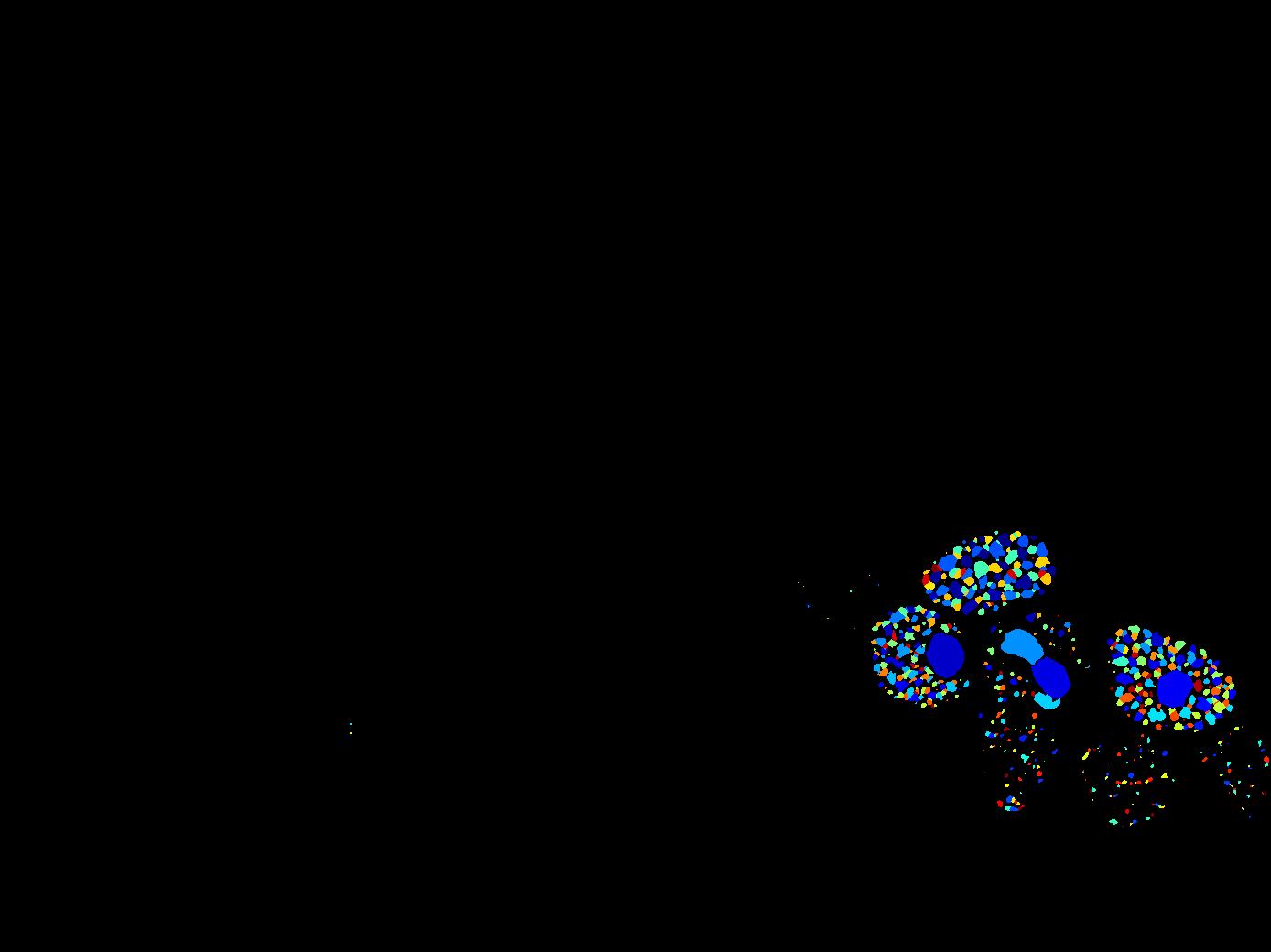

Supplement: Supplementary file 7 — Source Data [file 41467_2022_28822_MOESM7_ESM.zip › Figure 5E data/Masks/K136Q_I_06K136Q_48h 2_06_.jpeg]

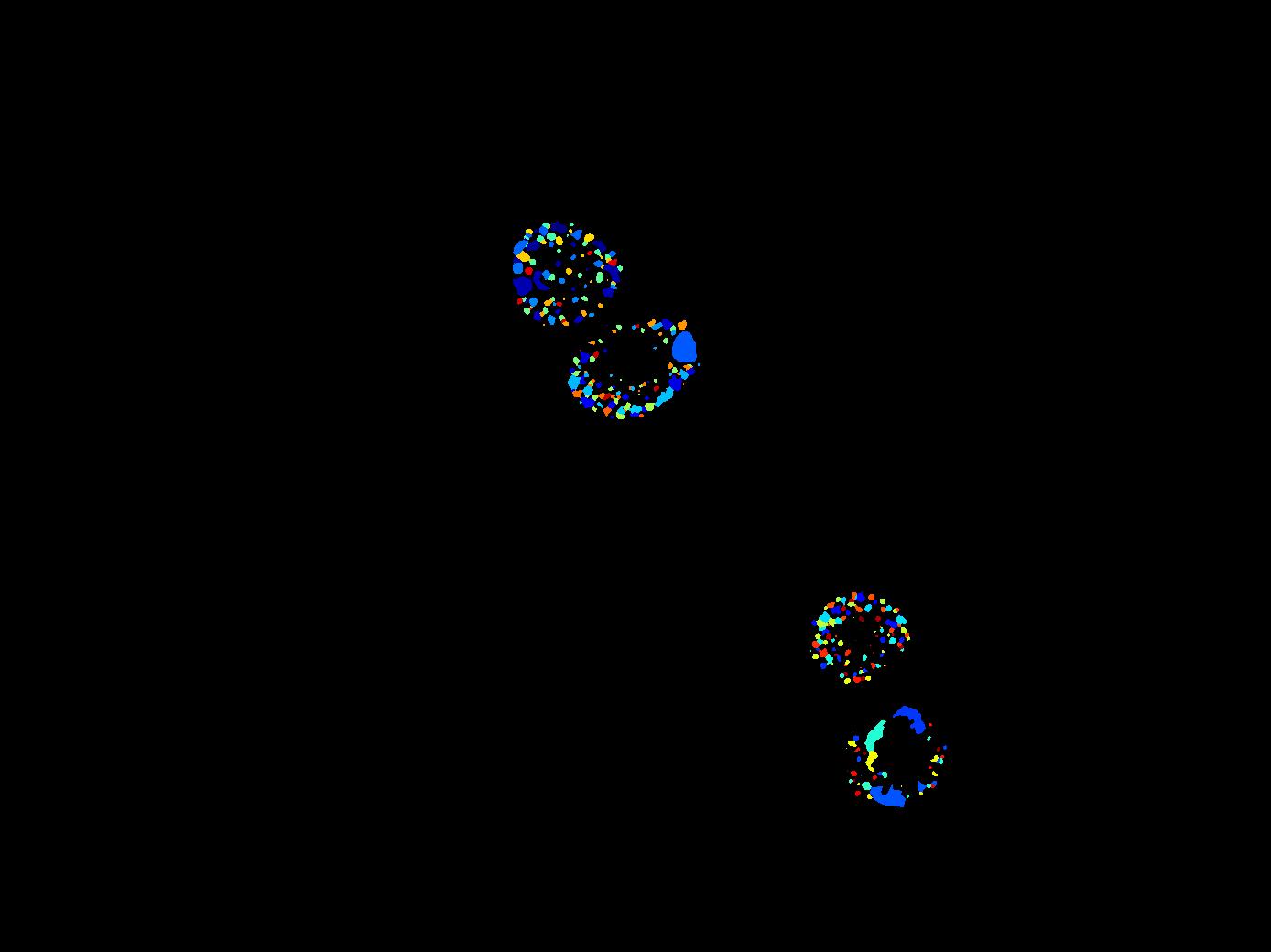

Supplement: Supplementary file 7 — Source Data [file 41467_2022_28822_MOESM7_ESM.zip › Figure 5E data/Masks/K136Q_I_06K136Q_48h_06_.jpeg]

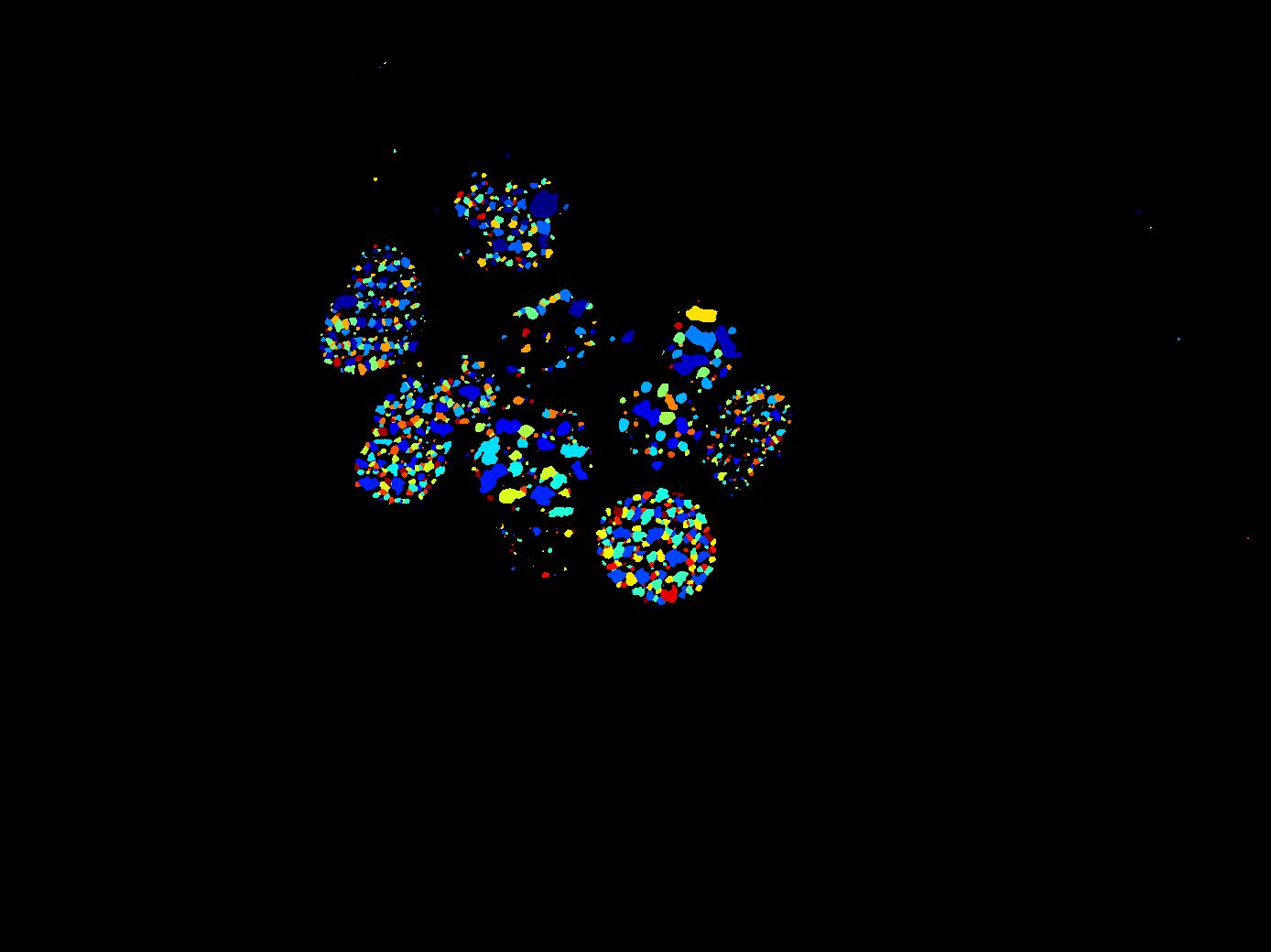

Supplement: Supplementary file 7 — Source Data [file 41467_2022_28822_MOESM7_ESM.zip › Figure 5E data/Masks/K136Q_I_06K136Q_72h 2_06_.jpeg]

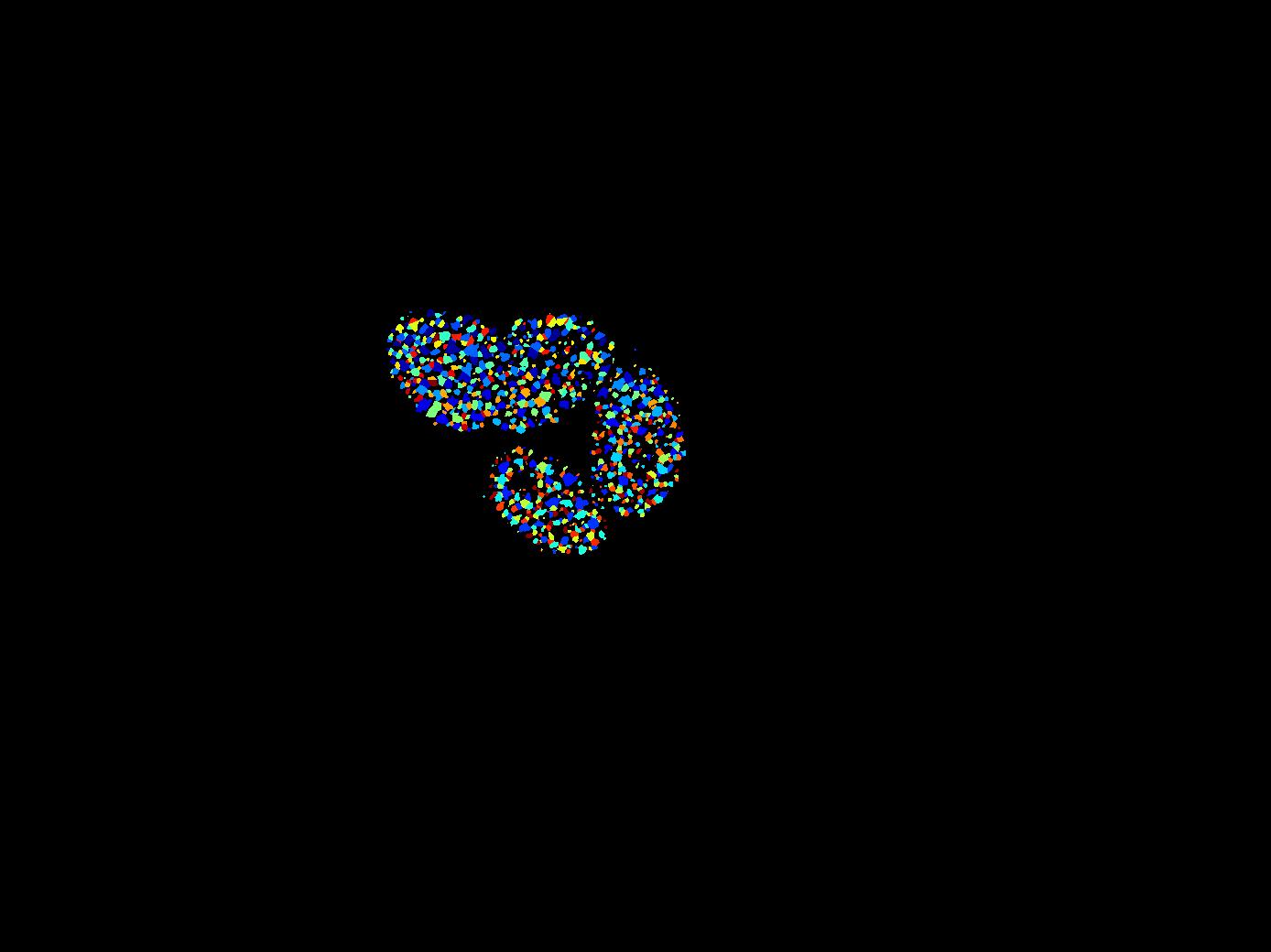

Supplement: Supplementary file 7 — Source Data [file 41467_2022_28822_MOESM7_ESM.zip › Figure 5E data/Masks/K136Q_I_07K136Q_24h 2_07_.jpeg]

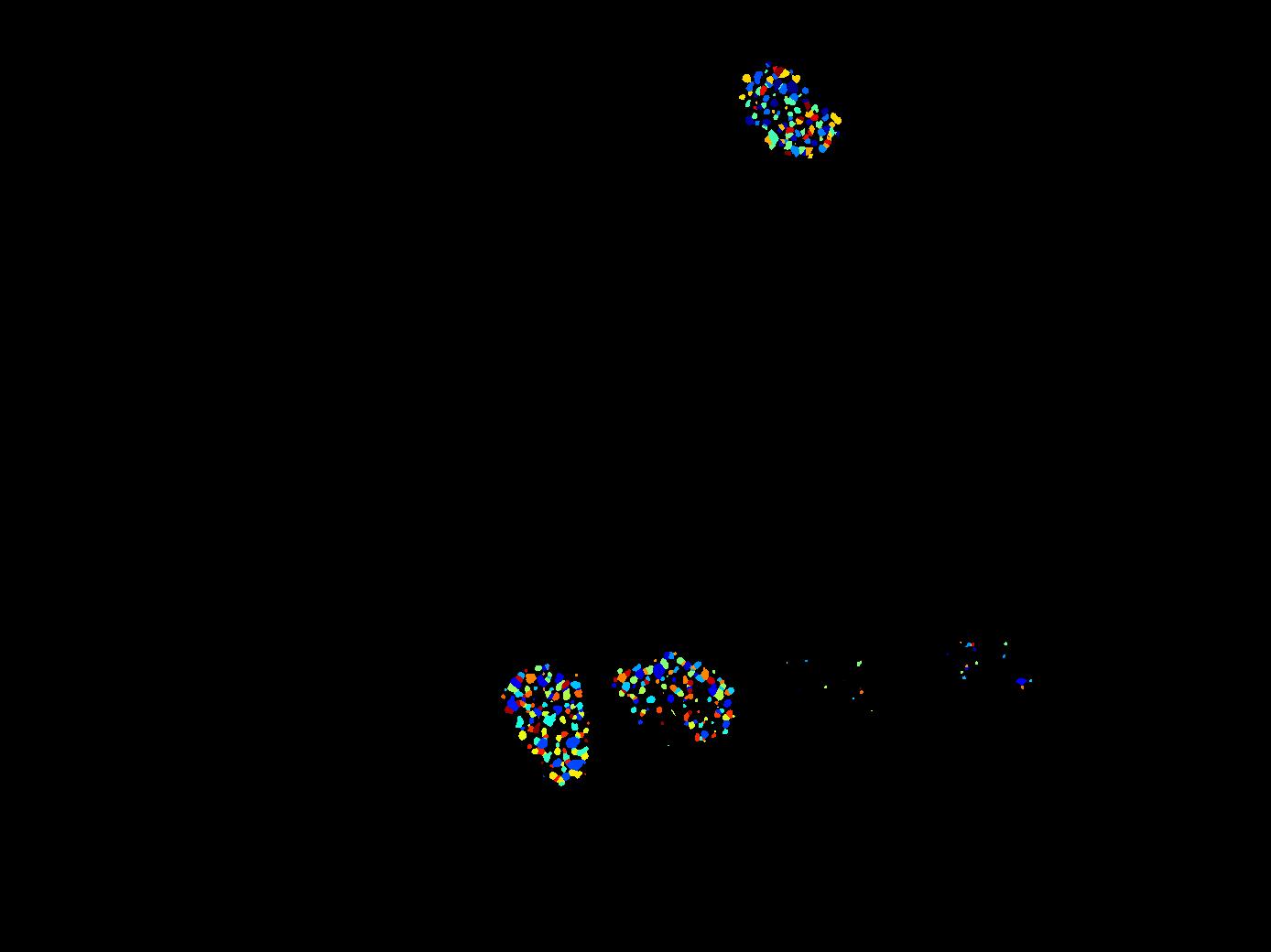

Supplement: Supplementary file 7 — Source Data [file 41467_2022_28822_MOESM7_ESM.zip › Figure 5E data/Masks/K136Q_I_07K136Q_24h_07_.jpeg]

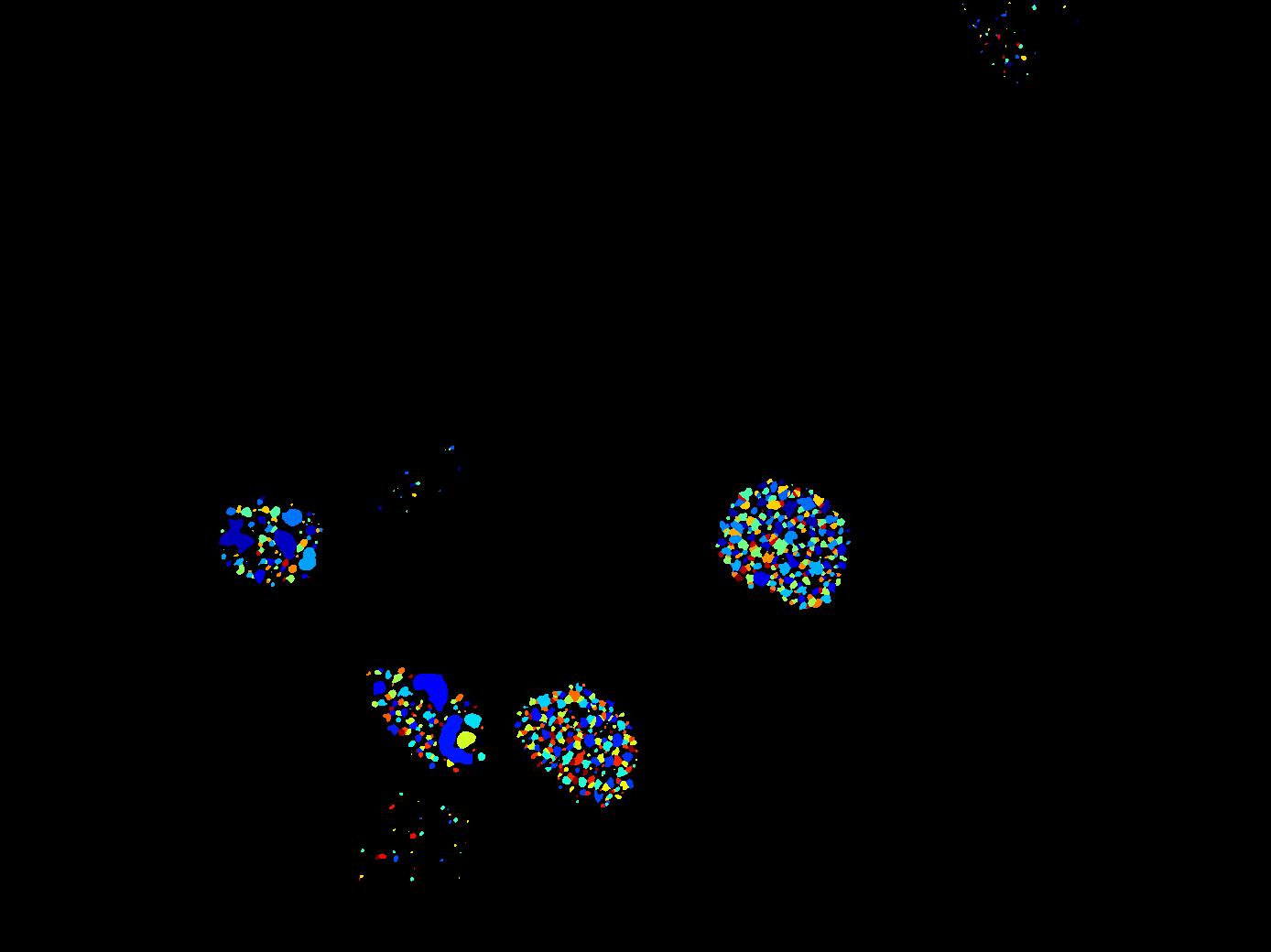

Supplement: Supplementary file 7 — Source Data [file 41467_2022_28822_MOESM7_ESM.zip › Figure 5E data/Masks/K136Q_I_07K136Q_48h 2_07_.jpeg]

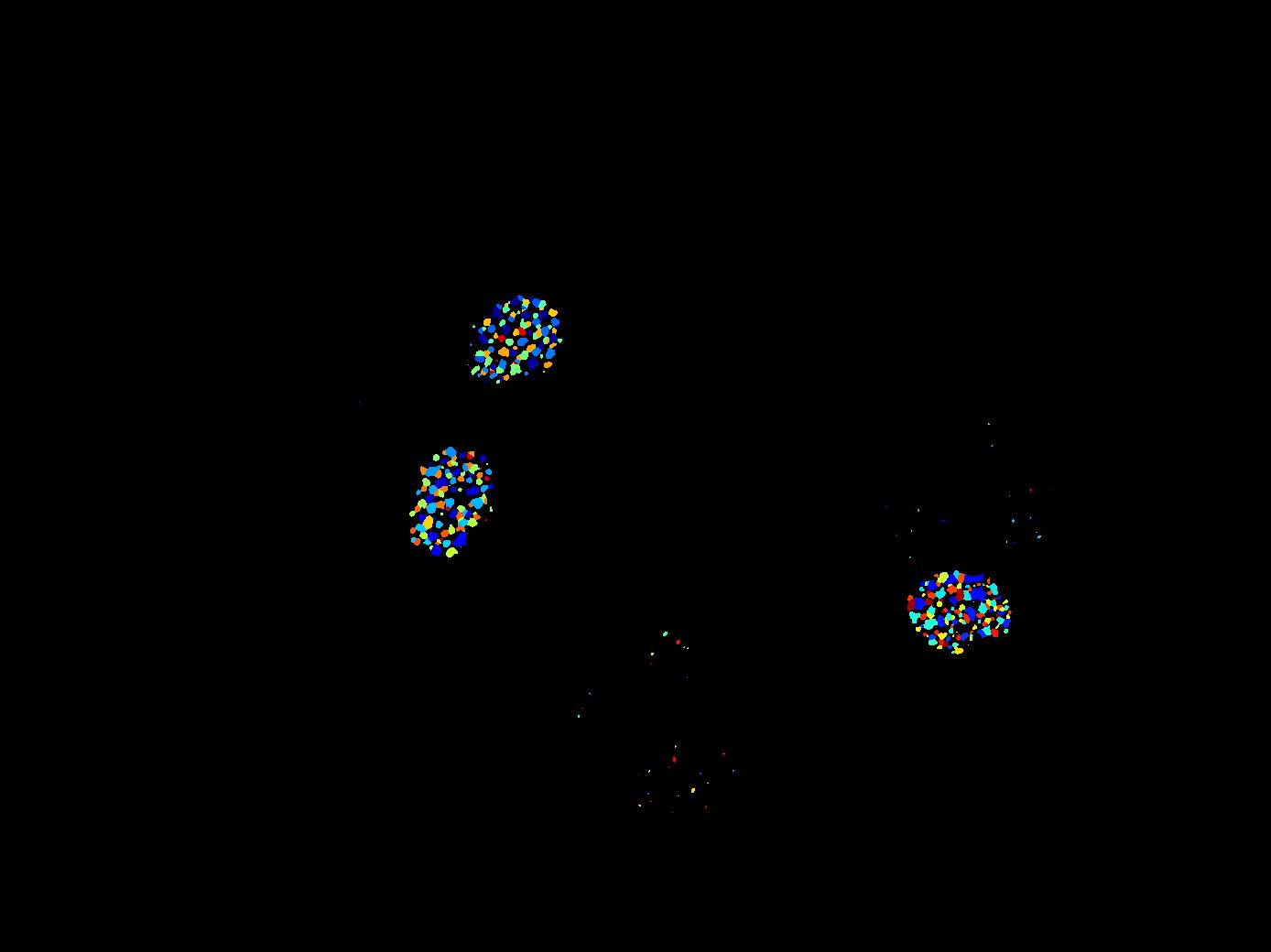

Supplement: Supplementary file 7 — Source Data [file 41467_2022_28822_MOESM7_ESM.zip › Figure 5E data/Masks/K136Q_I_07K136Q_48h_07_.jpeg]

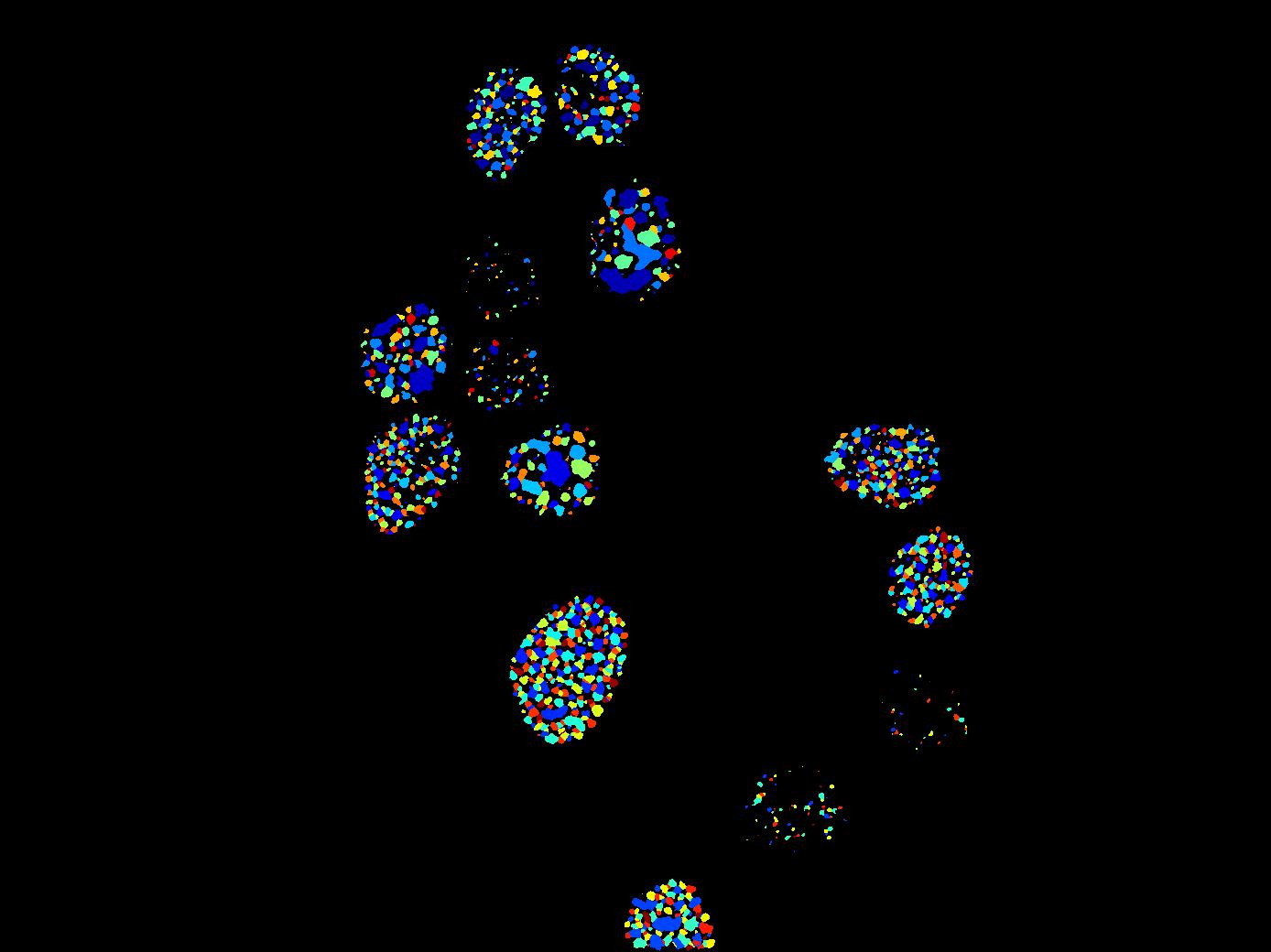

Supplement: Supplementary file 7 — Source Data [file 41467_2022_28822_MOESM7_ESM.zip › Figure 5E data/Masks/K136Q_I_07K136Q_72h 2_07_.jpeg]

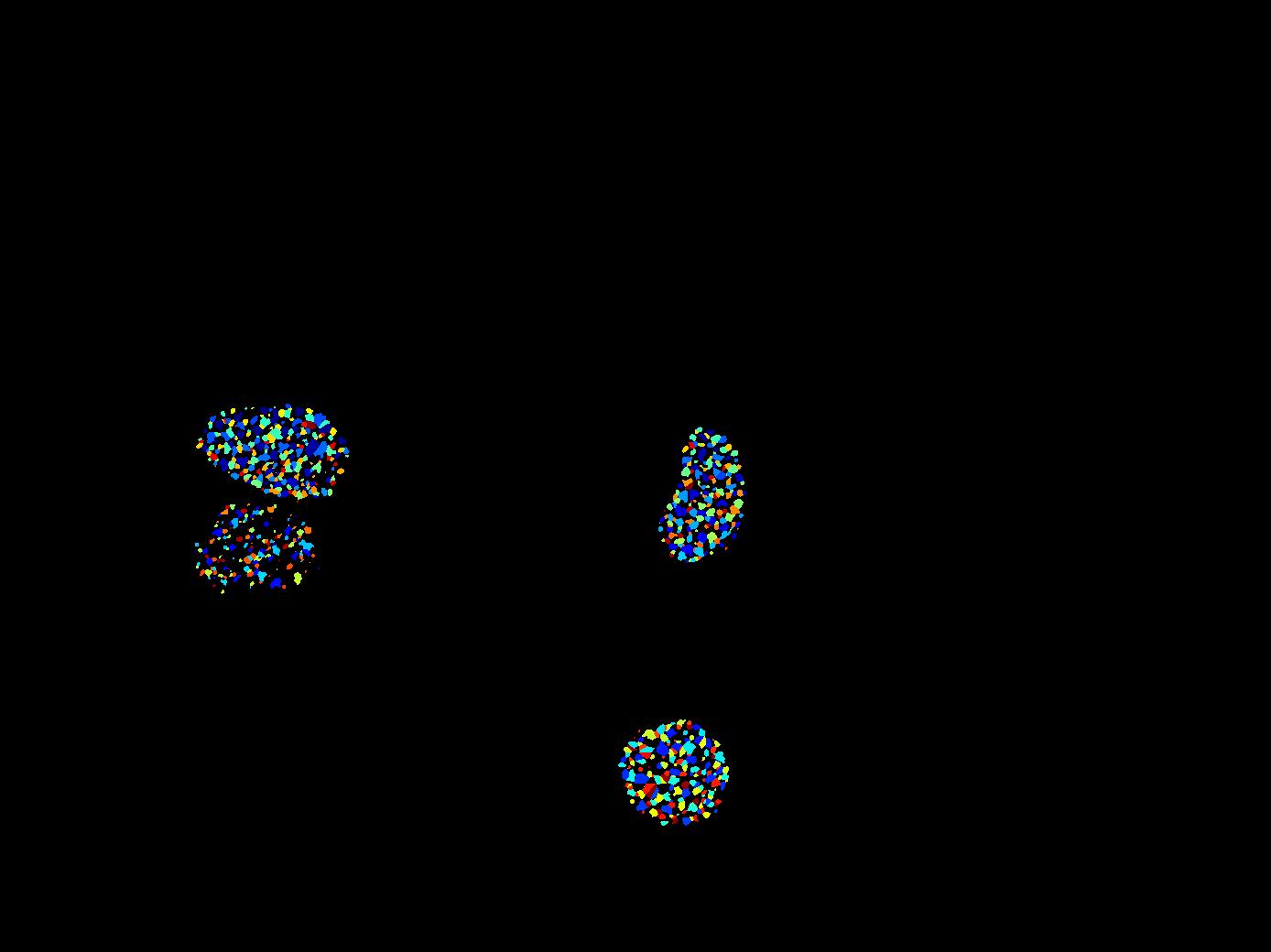

Supplement: Supplementary file 7 — Source Data [file 41467_2022_28822_MOESM7_ESM.zip › Figure 5E data/Masks/K136Q_I_08K136Q_24h 2_08_.jpeg]

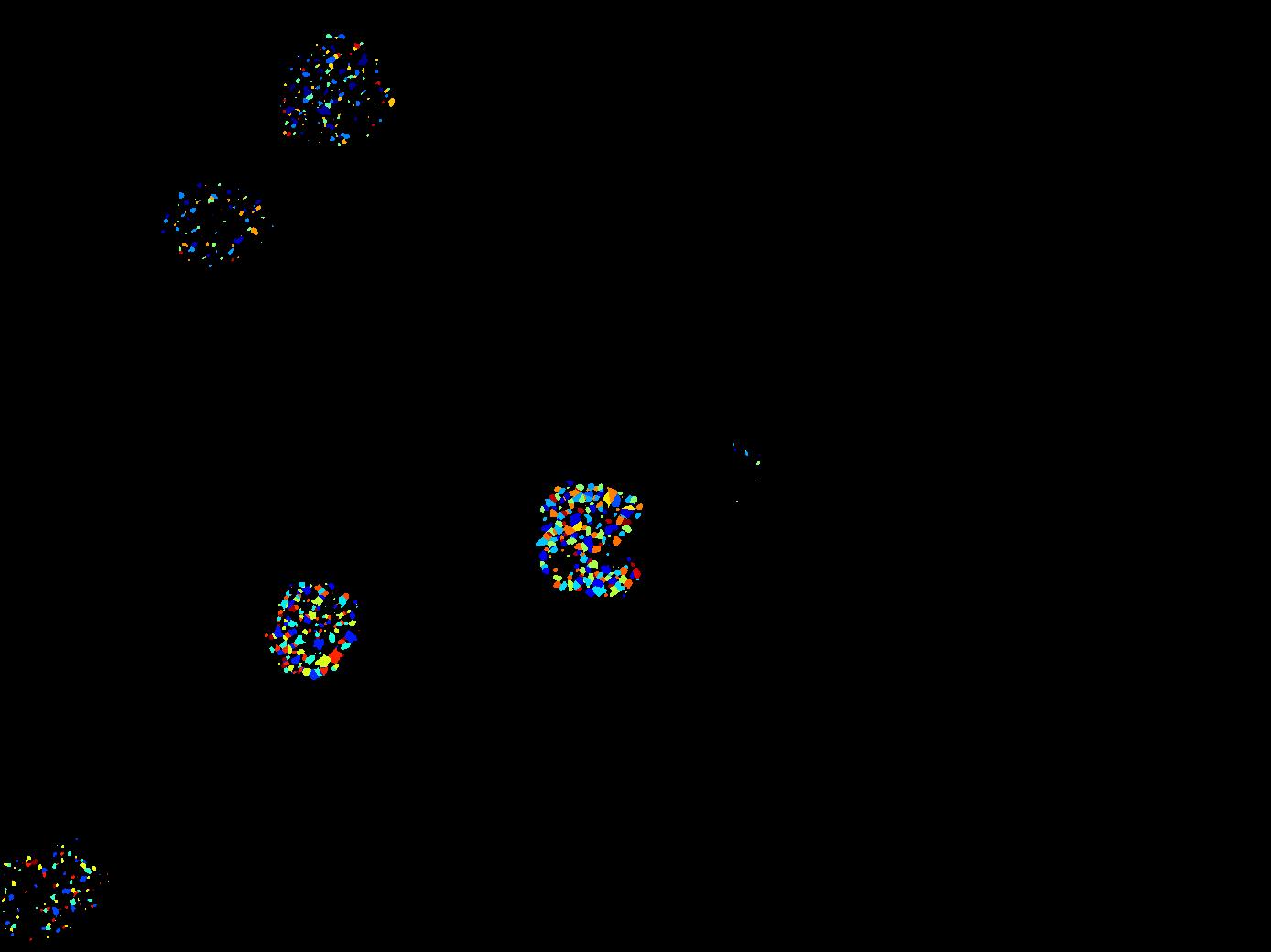

Supplement: Supplementary file 7 — Source Data [file 41467_2022_28822_MOESM7_ESM.zip › Figure 5E data/Masks/K136Q_I_08K136Q_24h_08_.jpeg]

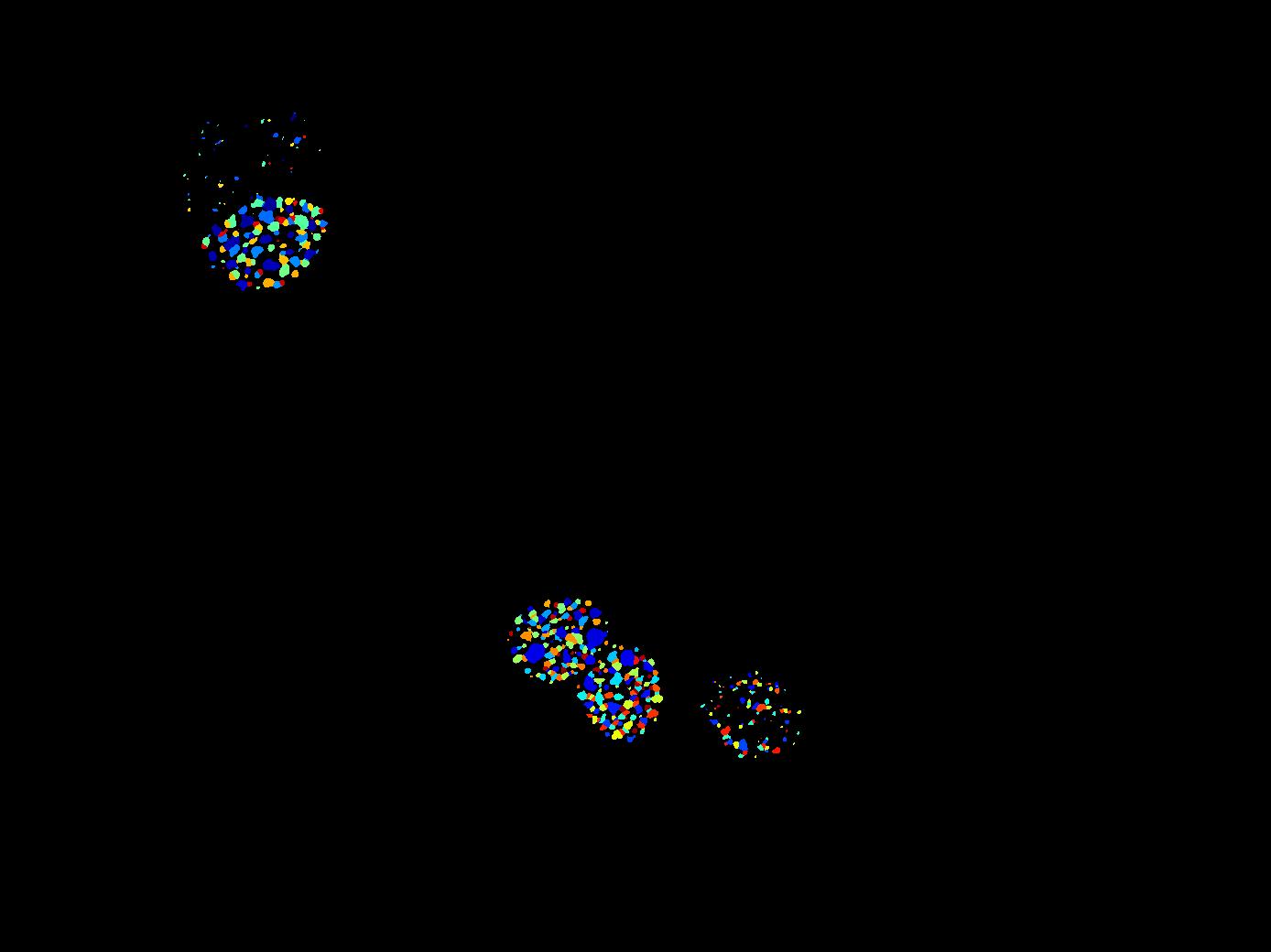

Supplement: Supplementary file 7 — Source Data [file 41467_2022_28822_MOESM7_ESM.zip › Figure 5E data/Masks/K136Q_I_08K136Q_48h 2_08_.jpeg]

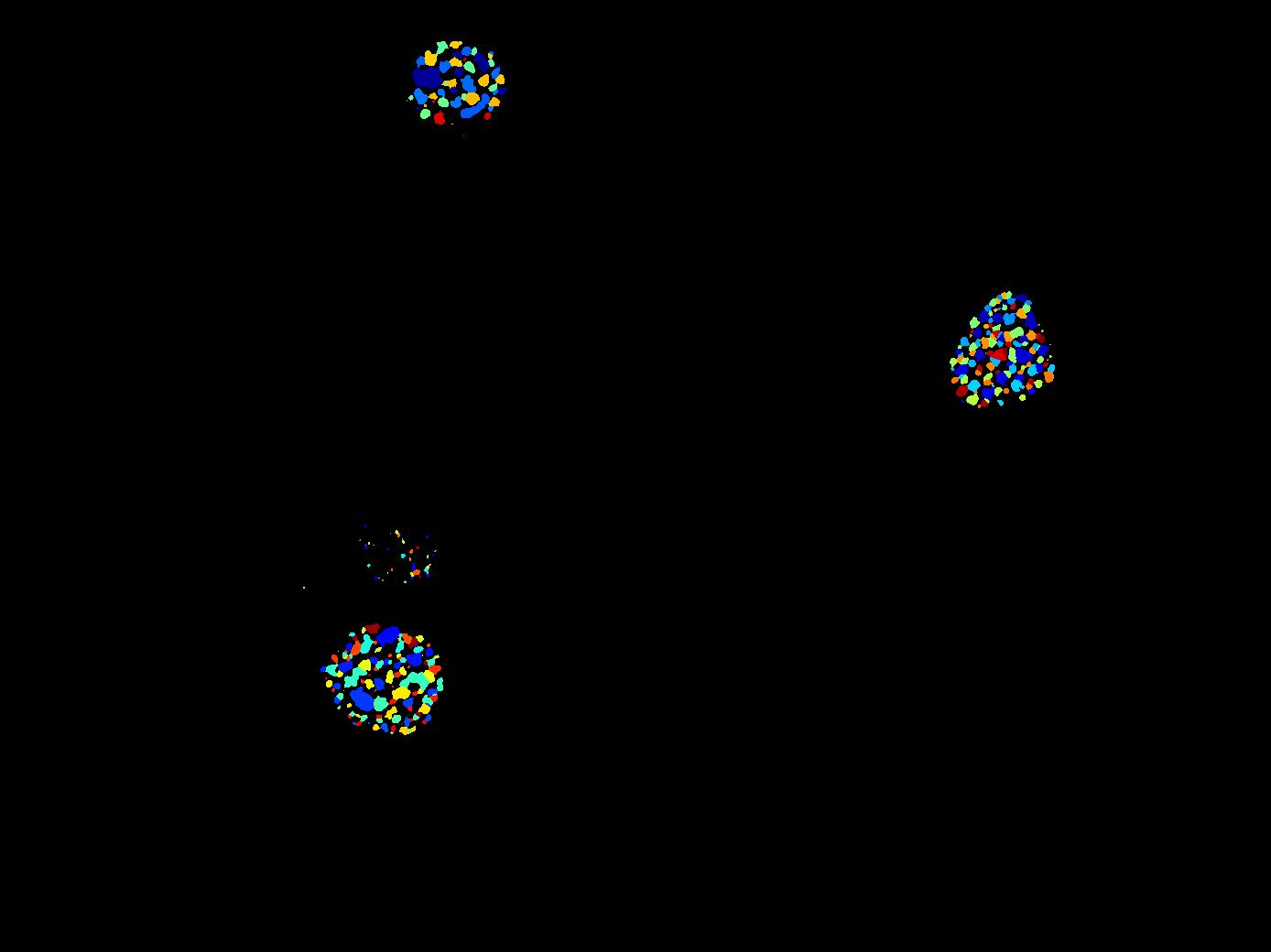

Supplement: Supplementary file 7 — Source Data [file 41467_2022_28822_MOESM7_ESM.zip › Figure 5E data/Masks/K136Q_I_08K136Q_48h_08_.jpeg]

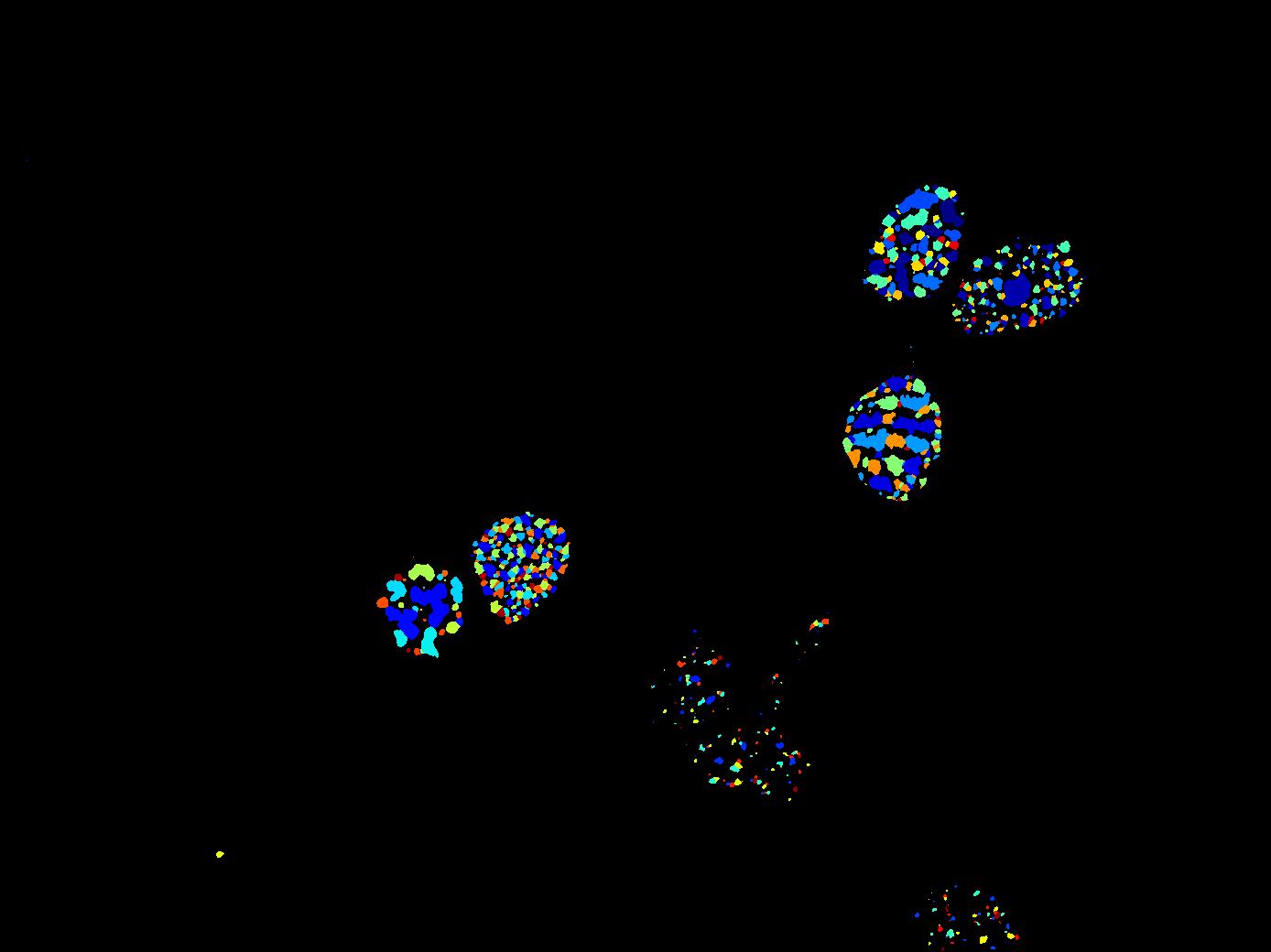

Supplement: Supplementary file 7 — Source Data [file 41467_2022_28822_MOESM7_ESM.zip › Figure 5E data/Masks/K136Q_I_08K136Q_72h 2_08_.jpeg]

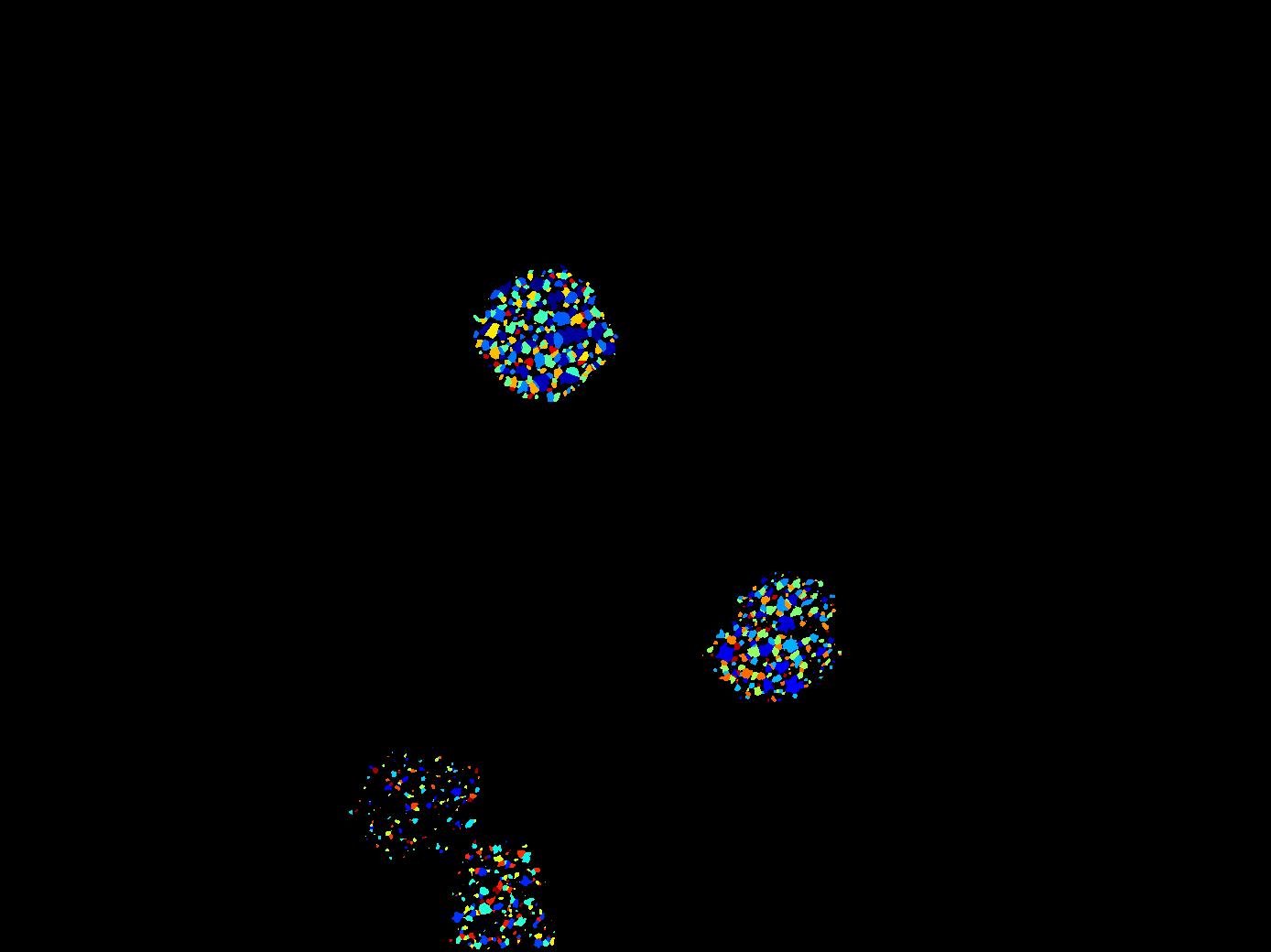

Supplement: Supplementary file 7 — Source Data [file 41467_2022_28822_MOESM7_ESM.zip › Figure 5E data/Masks/K136Q_I_09K136Q_24h 2_09_.jpeg]

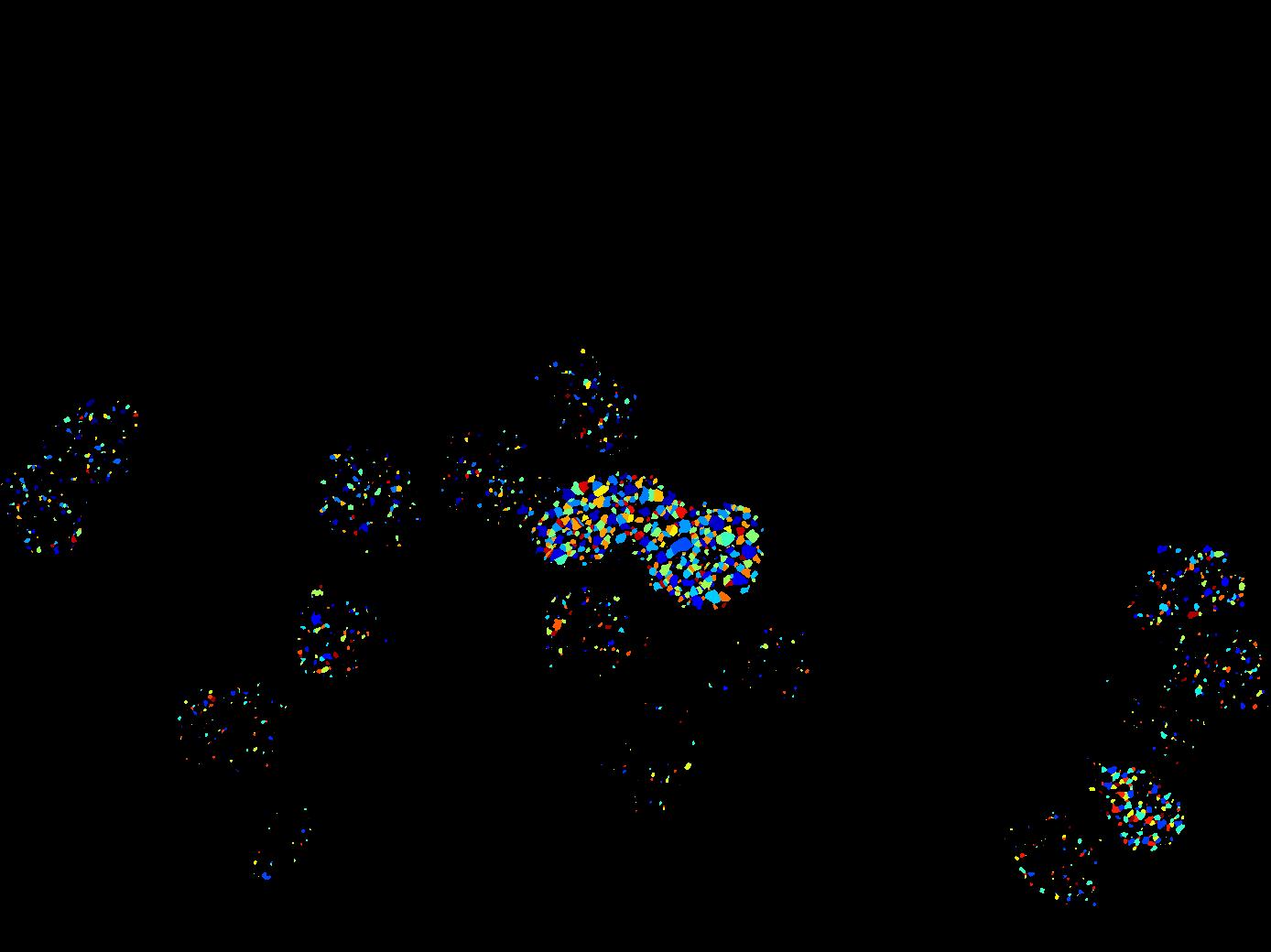

Supplement: Supplementary file 7 — Source Data [file 41467_2022_28822_MOESM7_ESM.zip › Figure 5E data/Masks/K136Q_I_09K136Q_24h_09_.jpeg]

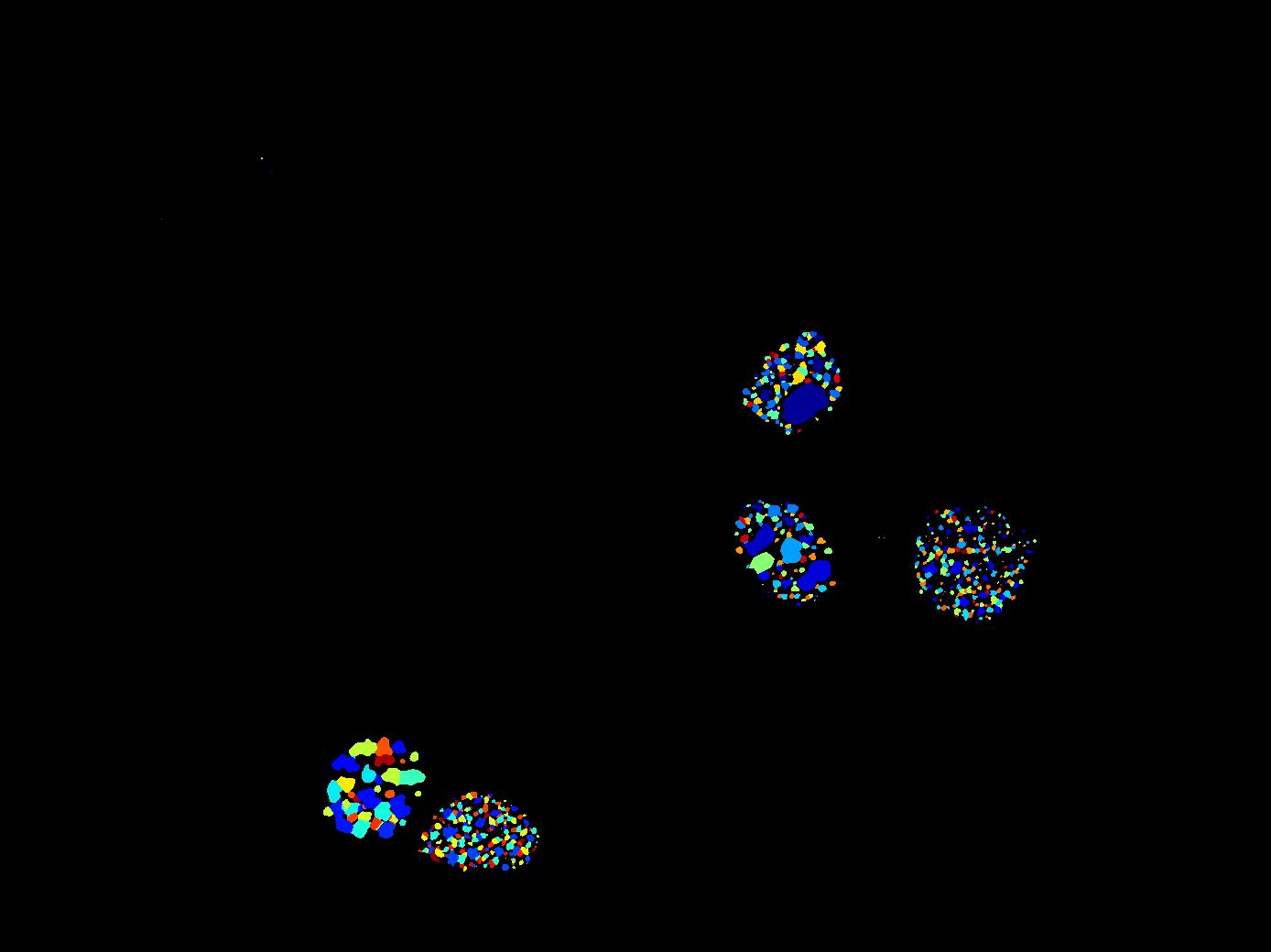

Supplement: Supplementary file 7 — Source Data [file 41467_2022_28822_MOESM7_ESM.zip › Figure 5E data/Masks/K136Q_I_09K136Q_48h 2_09_.jpeg]

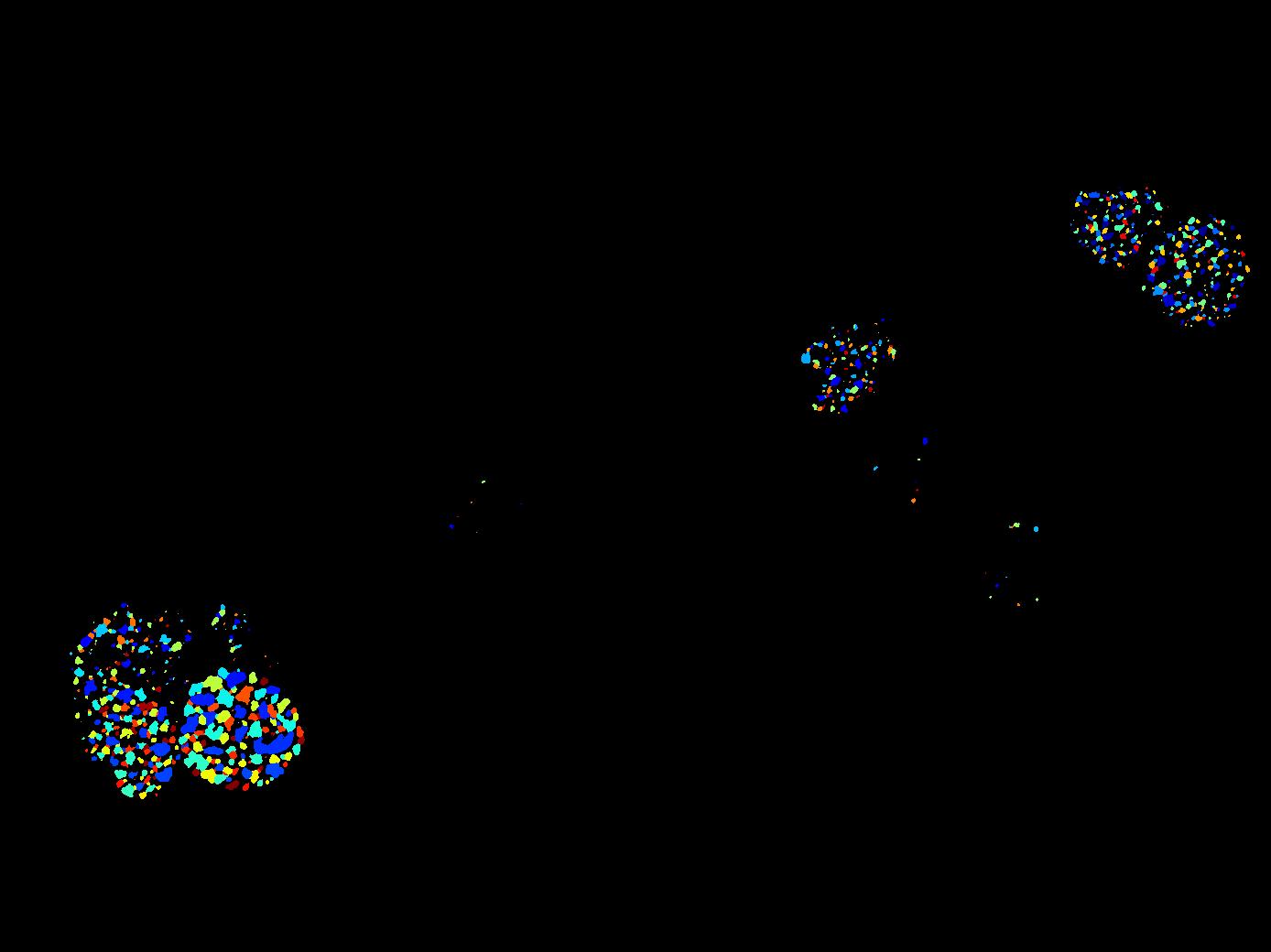

Supplement: Supplementary file 7 — Source Data [file 41467_2022_28822_MOESM7_ESM.zip › Figure 5E data/Masks/K136Q_I_09K136Q_48h_09_.jpeg]

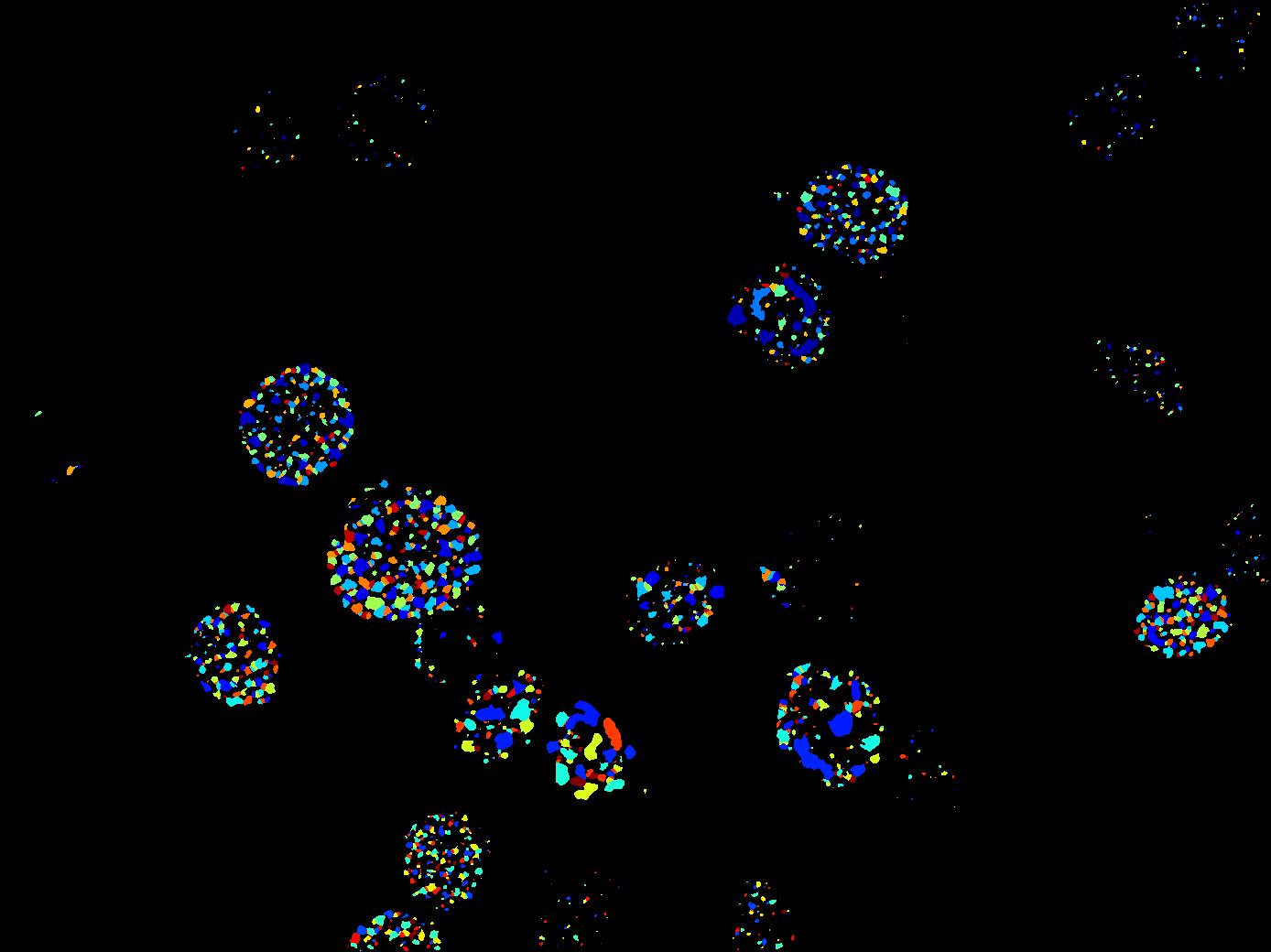

Supplement: Supplementary file 7 — Source Data [file 41467_2022_28822_MOESM7_ESM.zip › Figure 5E data/Masks/K136Q_I_09K136Q_72h 2_09_.jpeg]

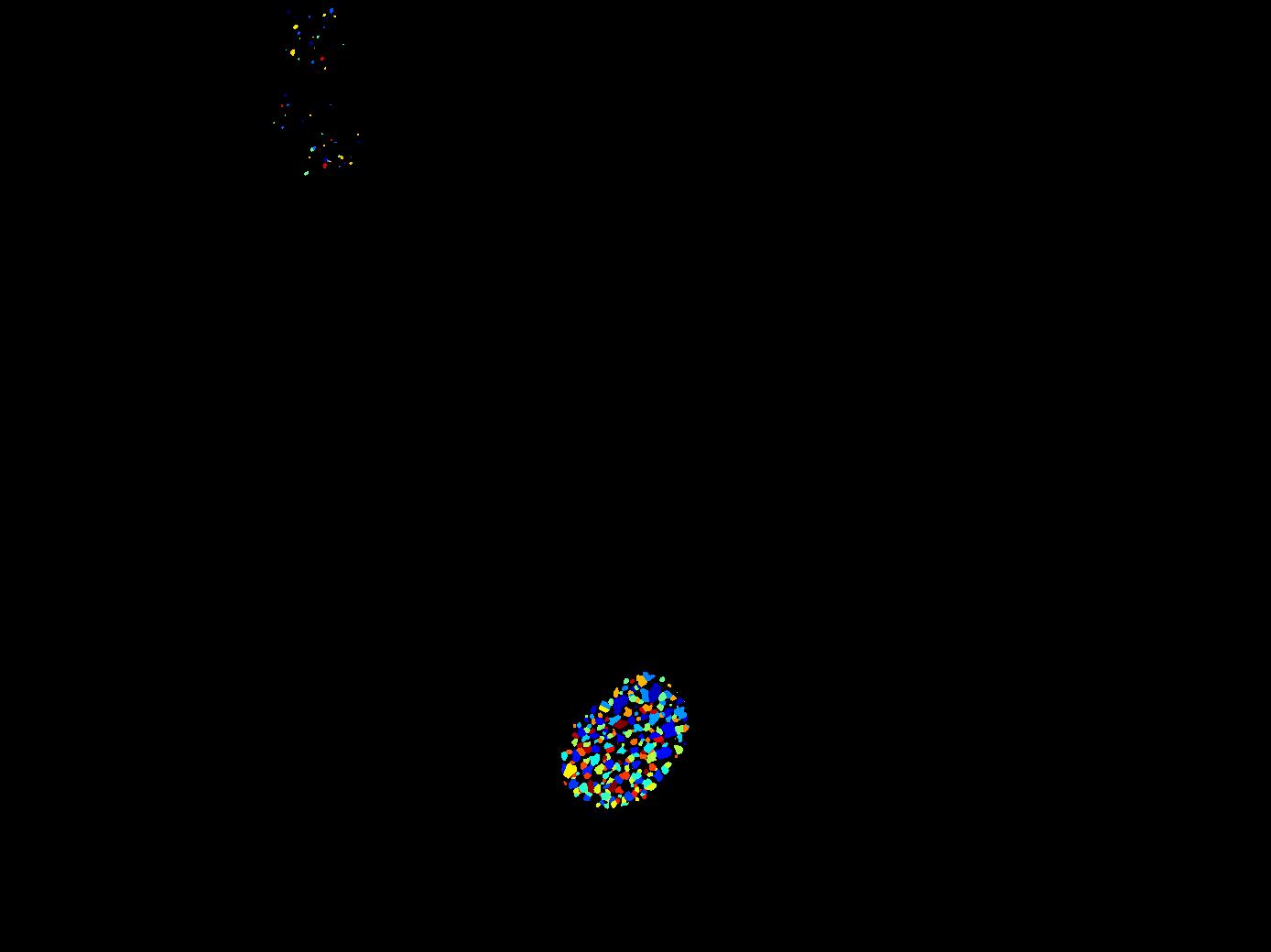

Supplement: Supplementary file 7 — Source Data [file 41467_2022_28822_MOESM7_ESM.zip › Figure 5E data/Masks/K136Q_I_10K136Q_24h 2_10_.jpeg]

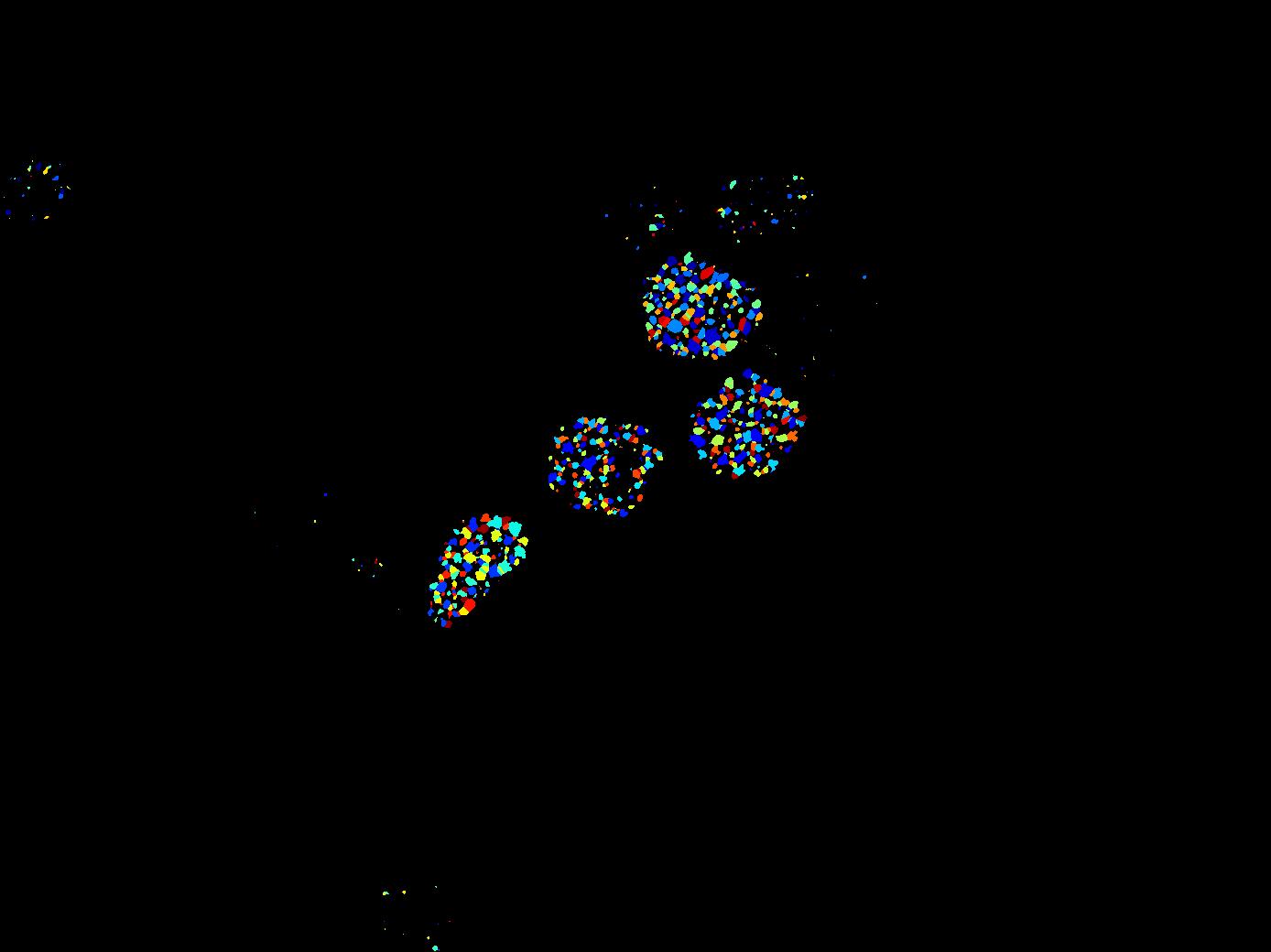

Supplement: Supplementary file 7 — Source Data [file 41467_2022_28822_MOESM7_ESM.zip › Figure 5E data/Masks/K136Q_I_10K136Q_24h_10_.jpeg]

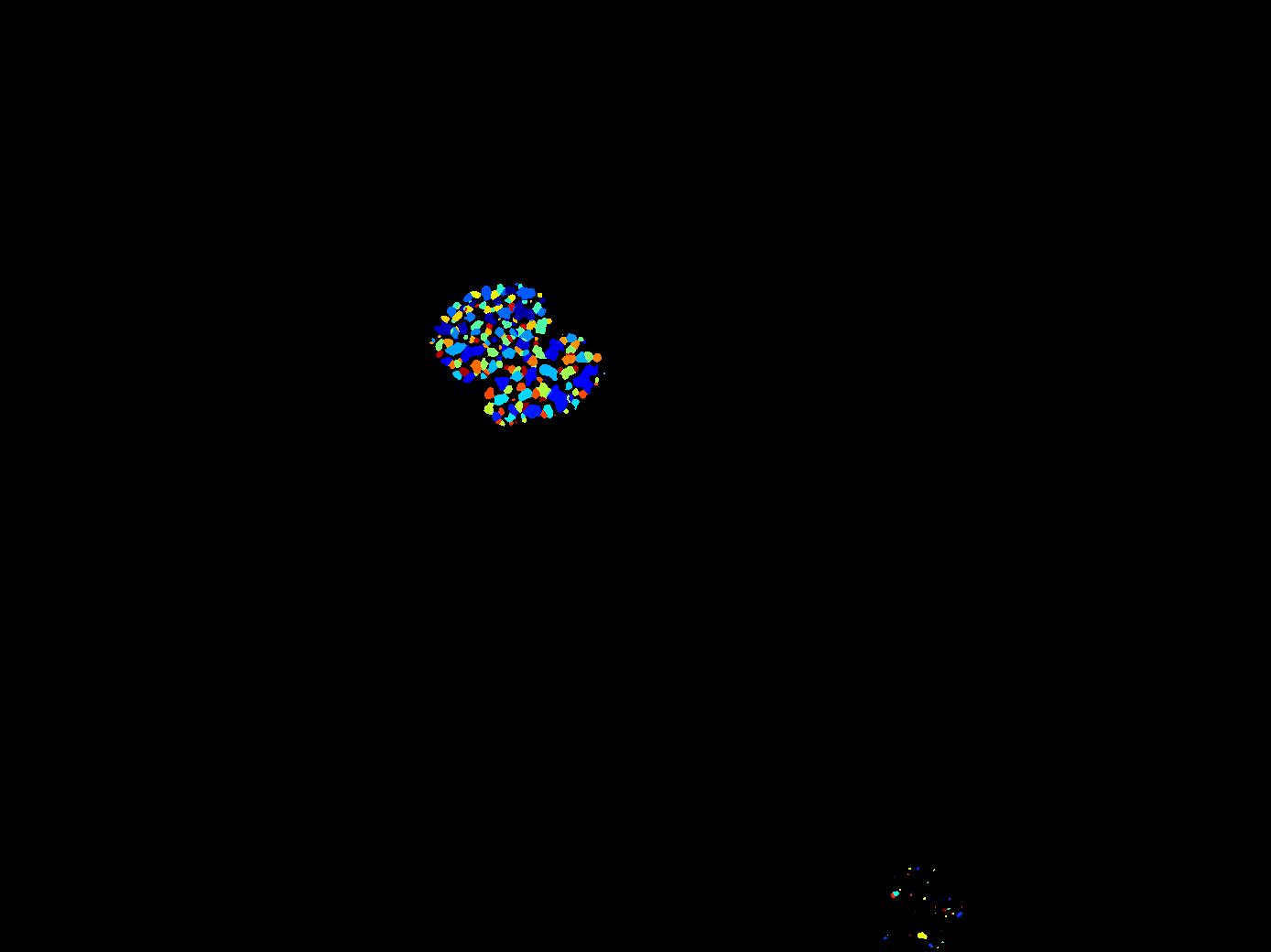

Supplement: Supplementary file 7 — Source Data [file 41467_2022_28822_MOESM7_ESM.zip › Figure 5E data/Masks/K136Q_I_10K136Q_48h 2_10_.jpeg]

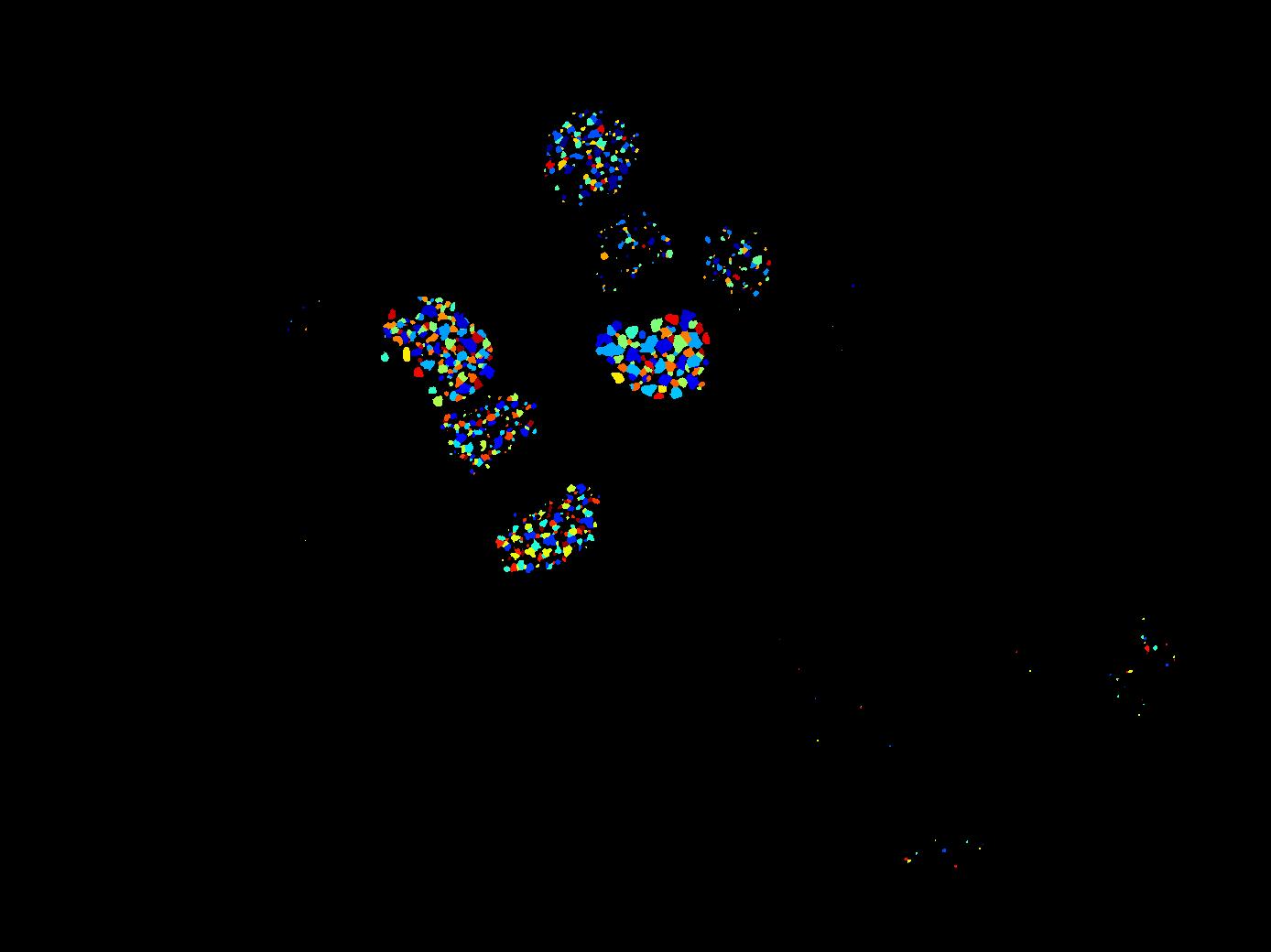

Supplement: Supplementary file 7 — Source Data [file 41467_2022_28822_MOESM7_ESM.zip › Figure 5E data/Masks/K136Q_I_10K136Q_48h_10_.jpeg]

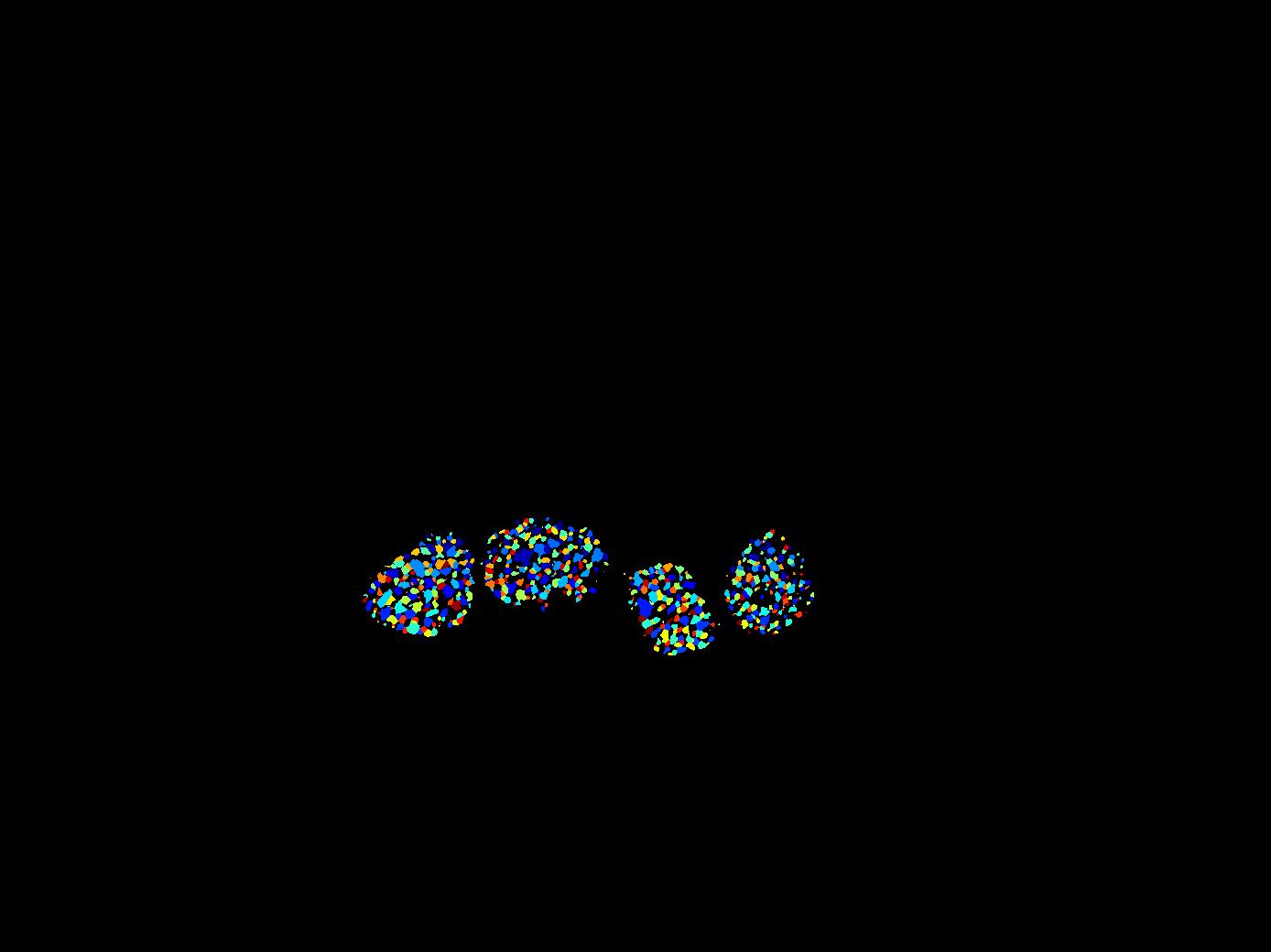

Supplement: Supplementary file 7 — Source Data [file 41467_2022_28822_MOESM7_ESM.zip › Figure 5E data/Masks/K136Q_I_11K136Q_24h 2_11_.jpeg]

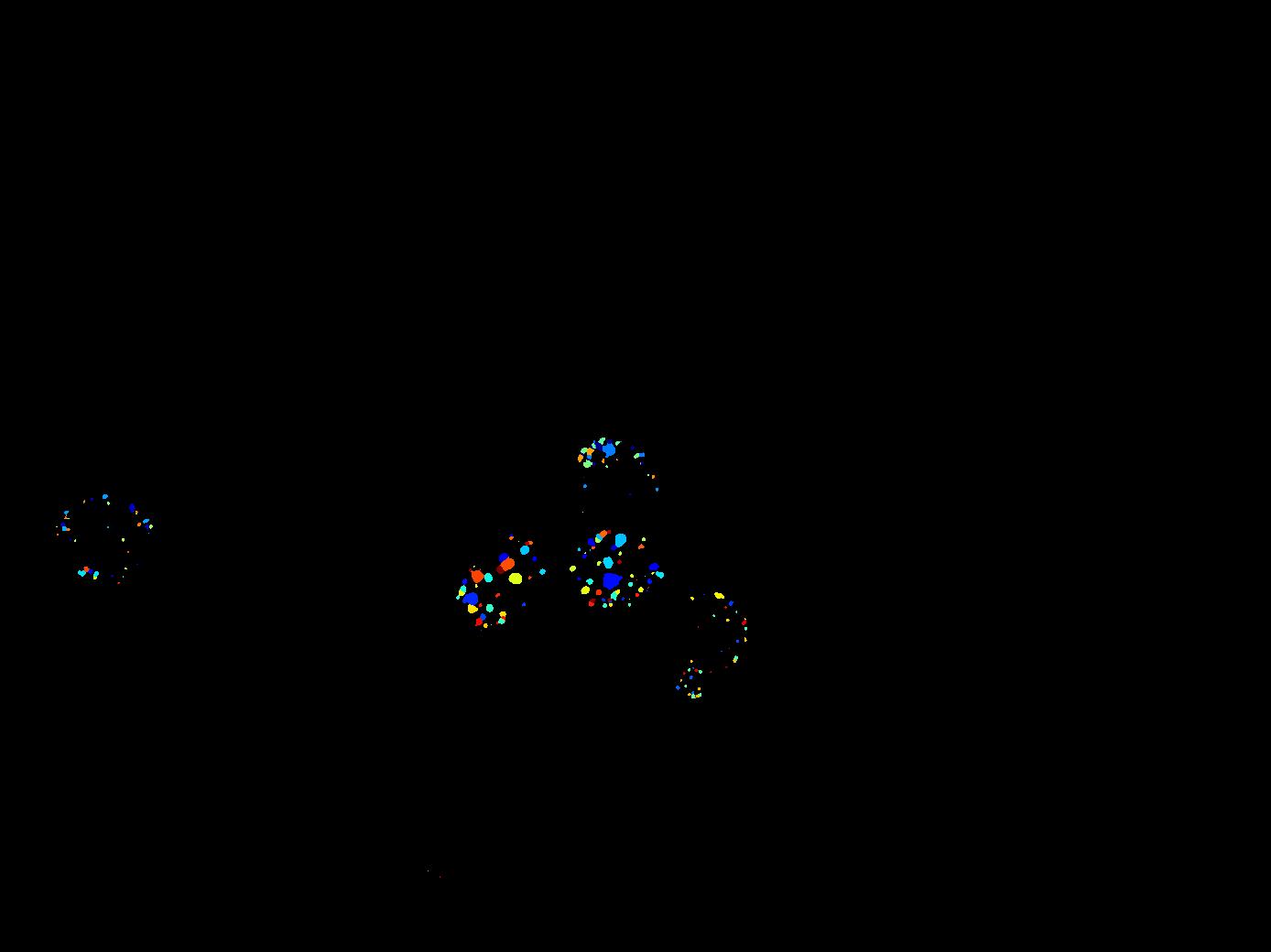

Supplement: Supplementary file 7 — Source Data [file 41467_2022_28822_MOESM7_ESM.zip › Figure 5E data/Masks/K136Q_I_11K136Q_24h_11_.jpeg]

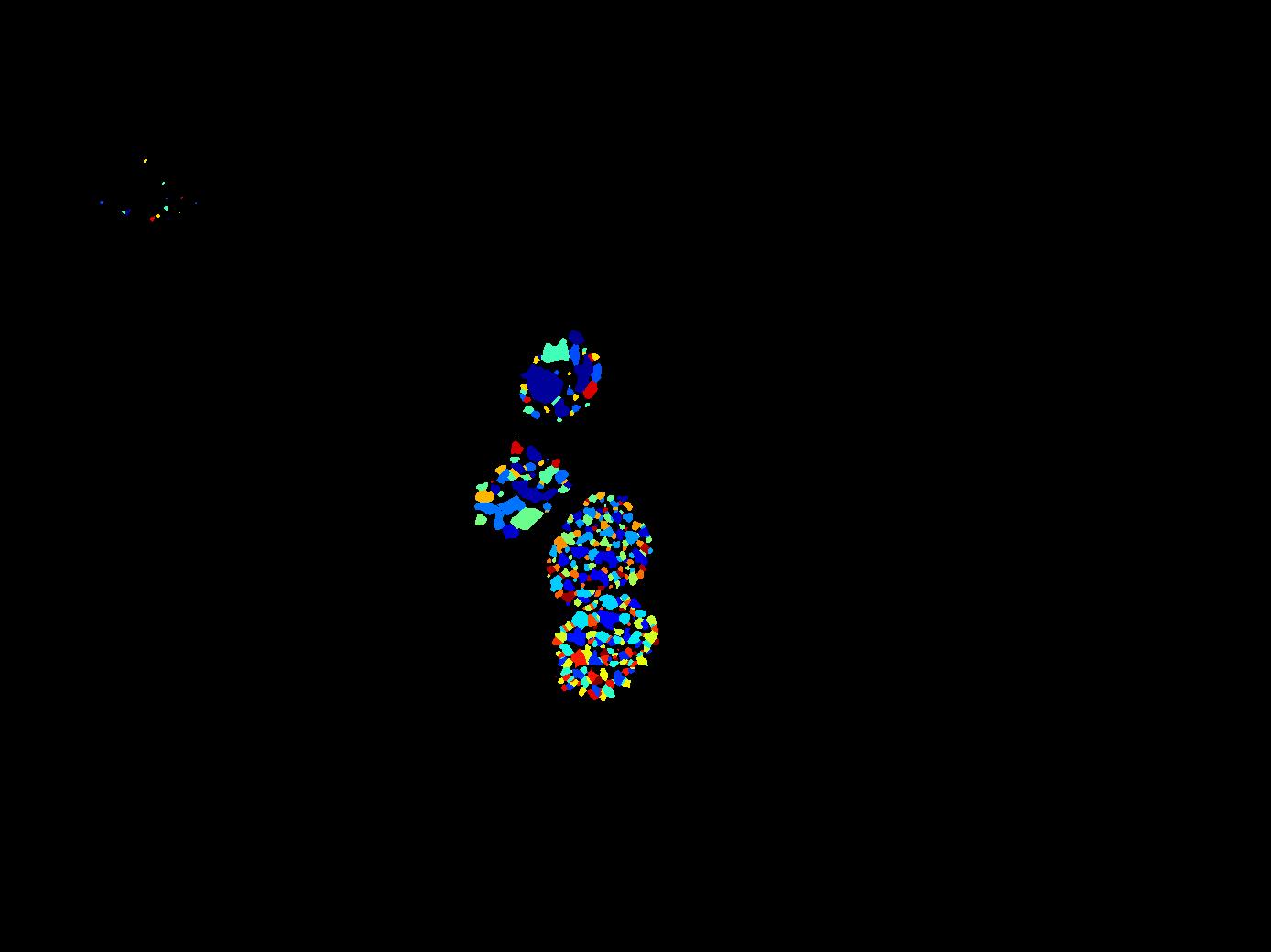

Supplement: Supplementary file 7 — Source Data [file 41467_2022_28822_MOESM7_ESM.zip › Figure 5E data/Masks/K136Q_I_11K136Q_48h 2_11_.jpeg]

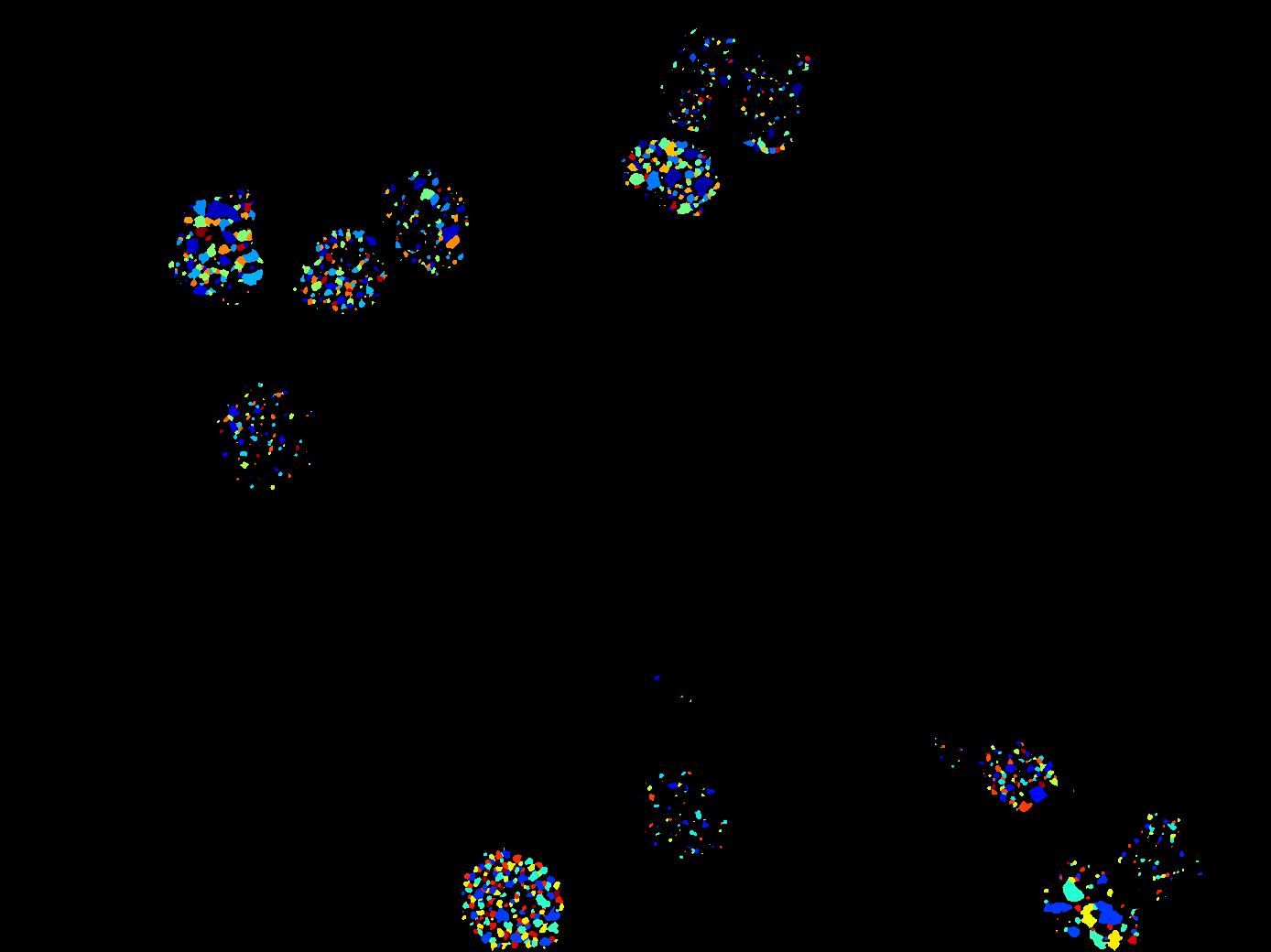

Supplement: Supplementary file 7 — Source Data [file 41467_2022_28822_MOESM7_ESM.zip › Figure 5E data/Masks/K136Q_I_11K136Q_48h_11_.jpeg]

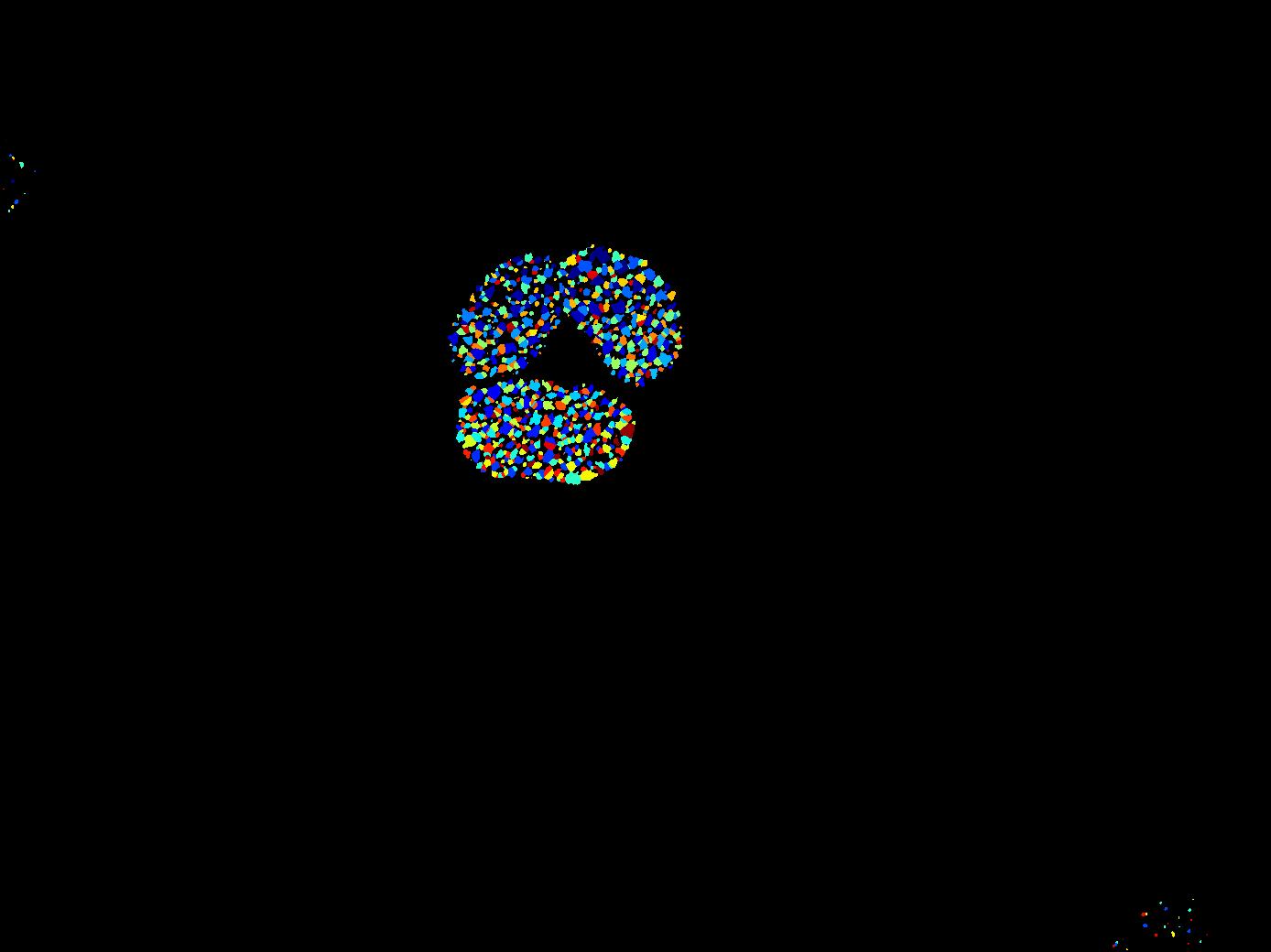

Supplement: Supplementary file 7 — Source Data [file 41467_2022_28822_MOESM7_ESM.zip › Figure 5E data/Masks/K136Q_I_12K136Q_24h 2_12_.jpeg]

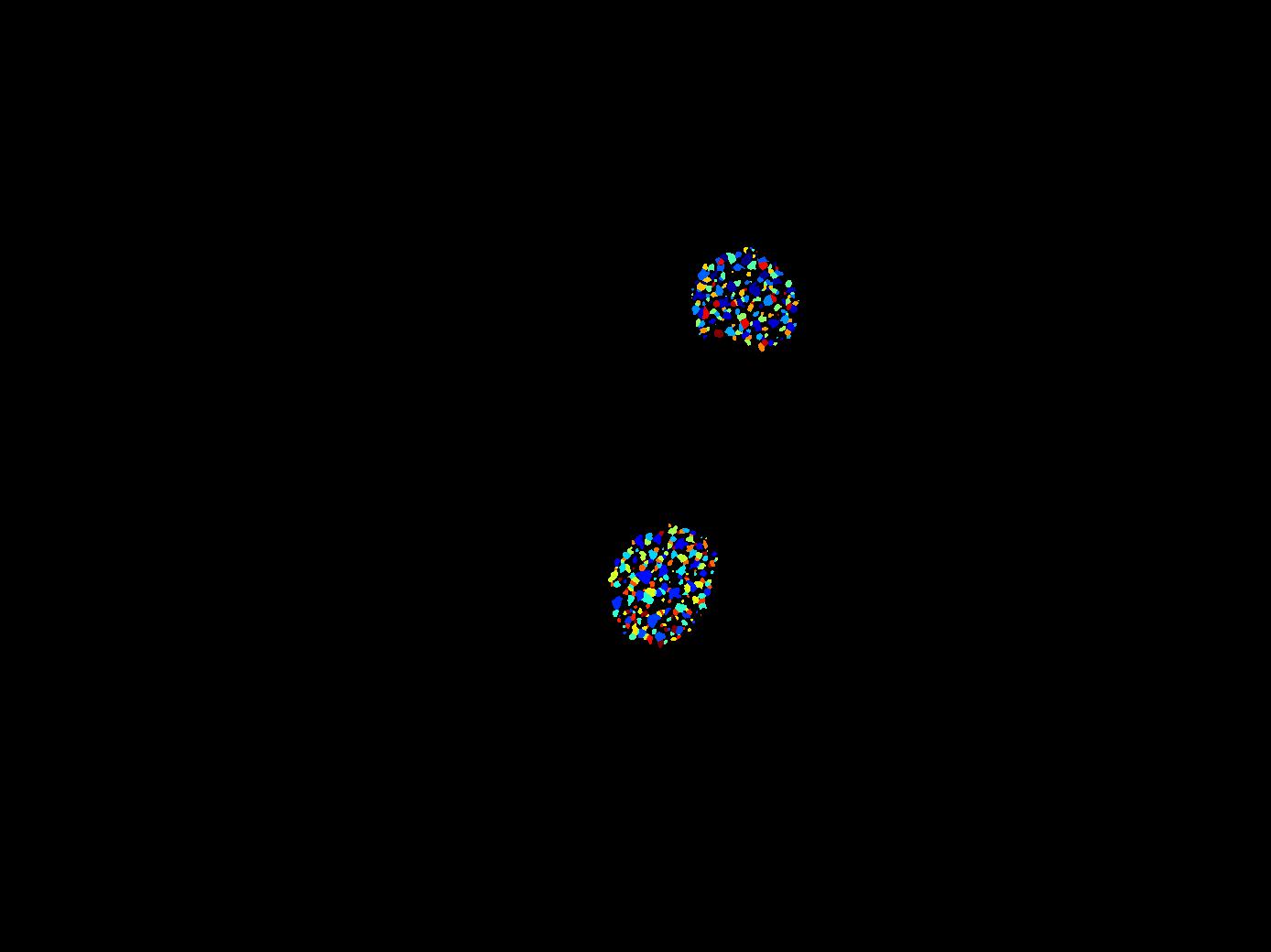

Supplement: Supplementary file 7 — Source Data [file 41467_2022_28822_MOESM7_ESM.zip › Figure 5E data/Masks/K136Q_I_12K136Q_24h_12_.jpeg]

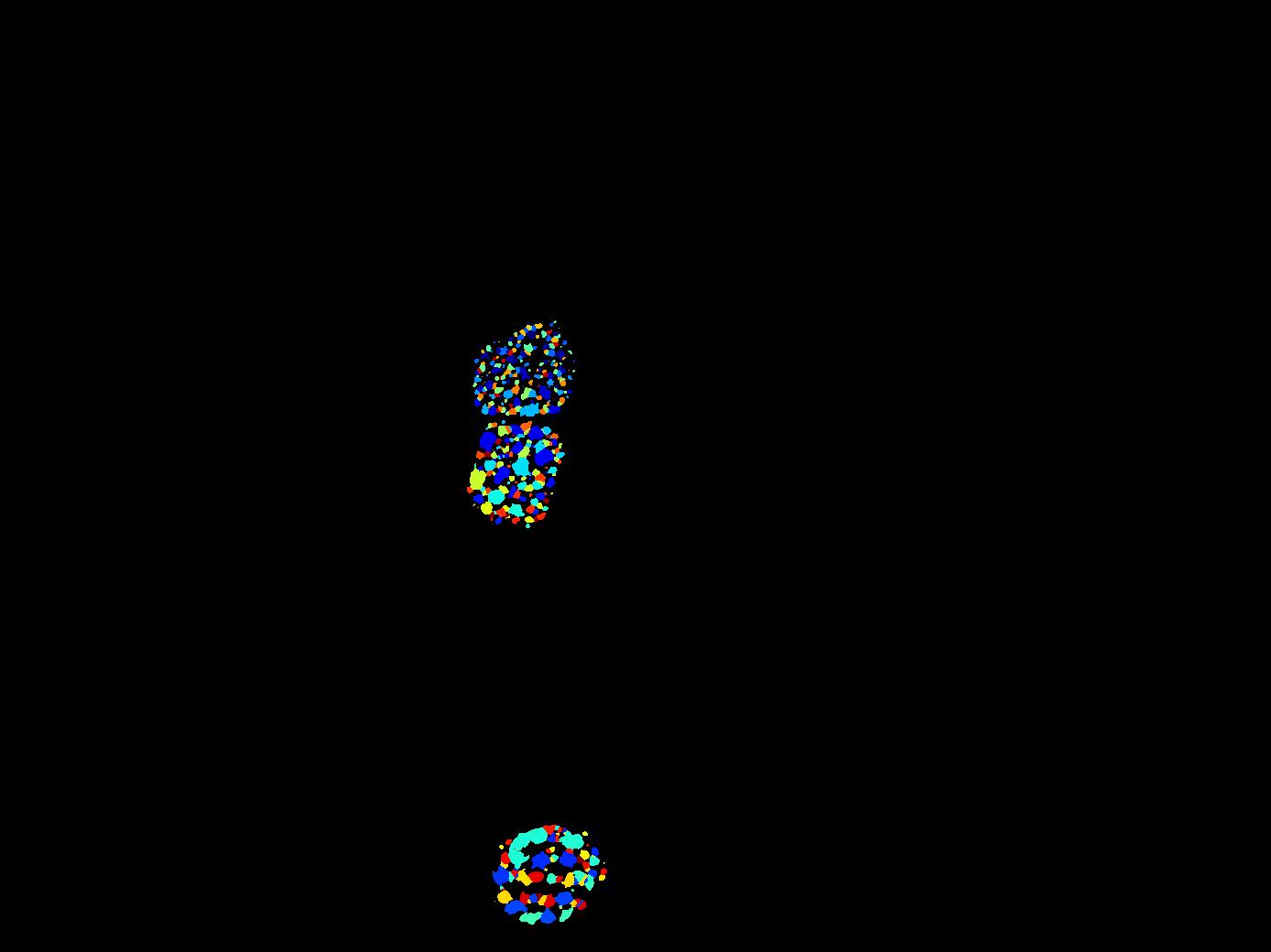

Supplement: Supplementary file 7 — Source Data [file 41467_2022_28822_MOESM7_ESM.zip › Figure 5E data/Masks/K136Q_I_12K136Q_48h 2_12_.jpeg]

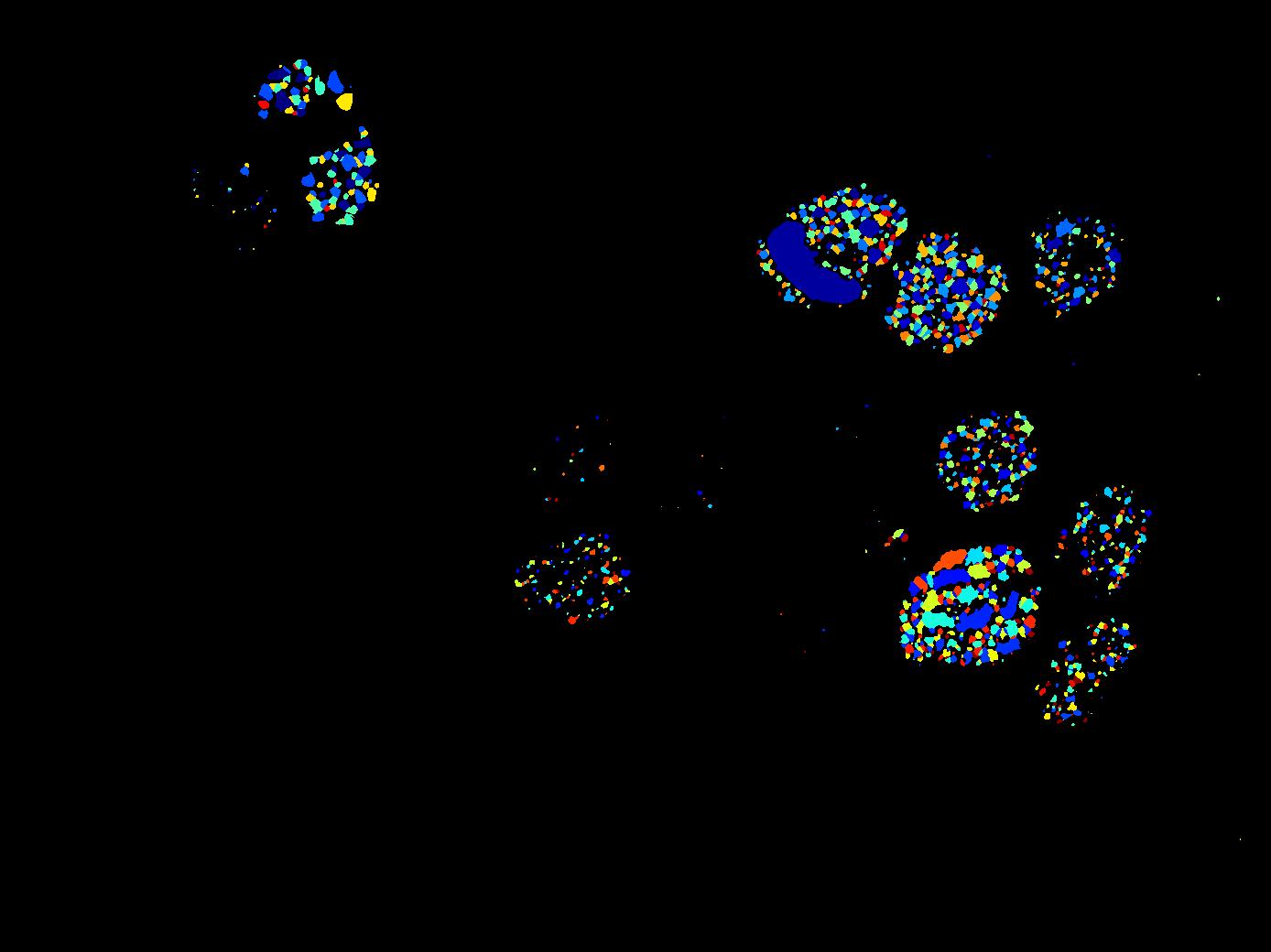

Supplement: Supplementary file 7 — Source Data [file 41467_2022_28822_MOESM7_ESM.zip › Figure 5E data/Masks/K136Q_I_12K136Q_48h_12_.jpeg]

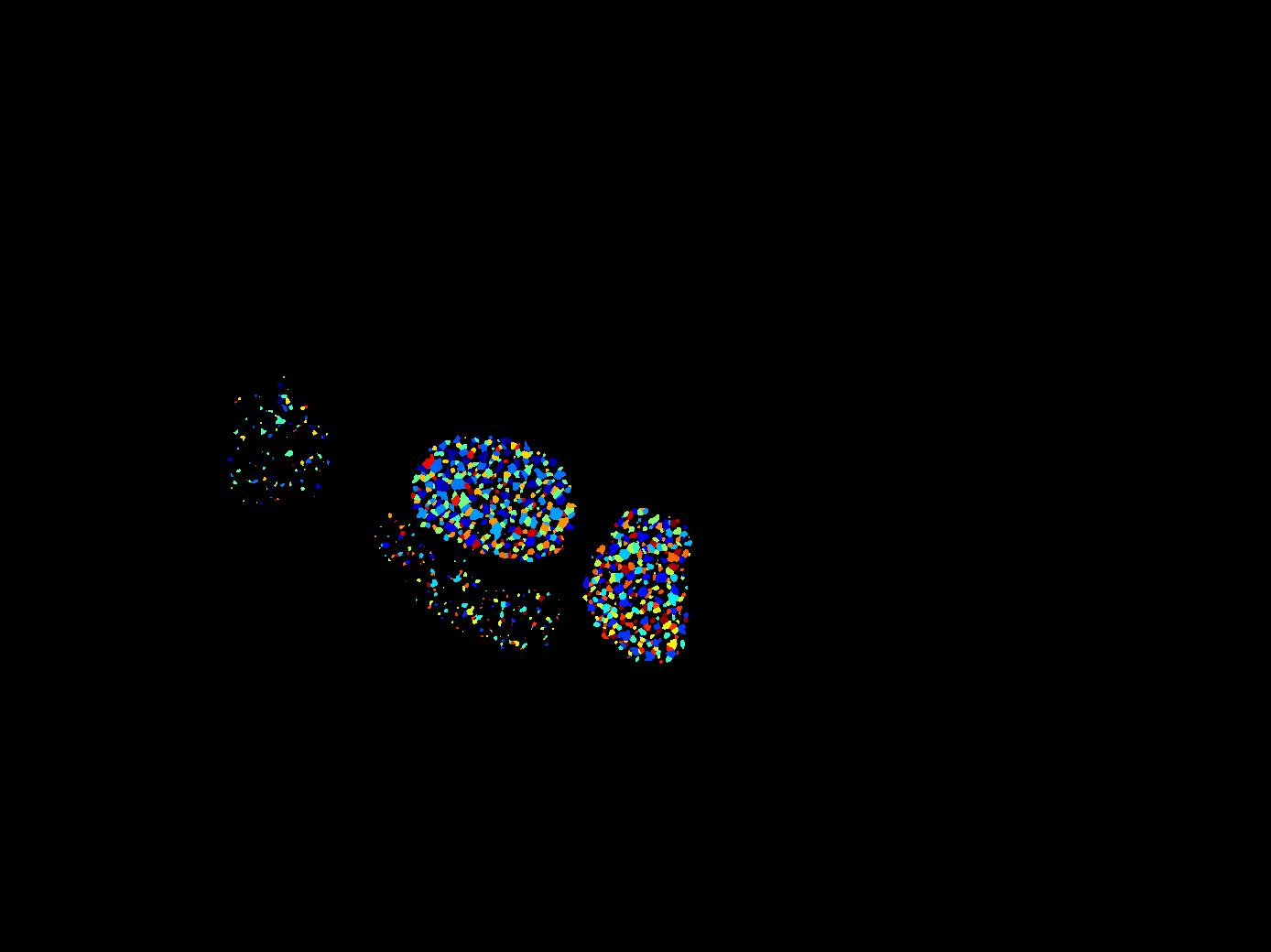

Supplement: Supplementary file 7 — Source Data [file 41467_2022_28822_MOESM7_ESM.zip › Figure 5E data/Masks/K136Q_I_13K136Q_24h 2_13_.jpeg]

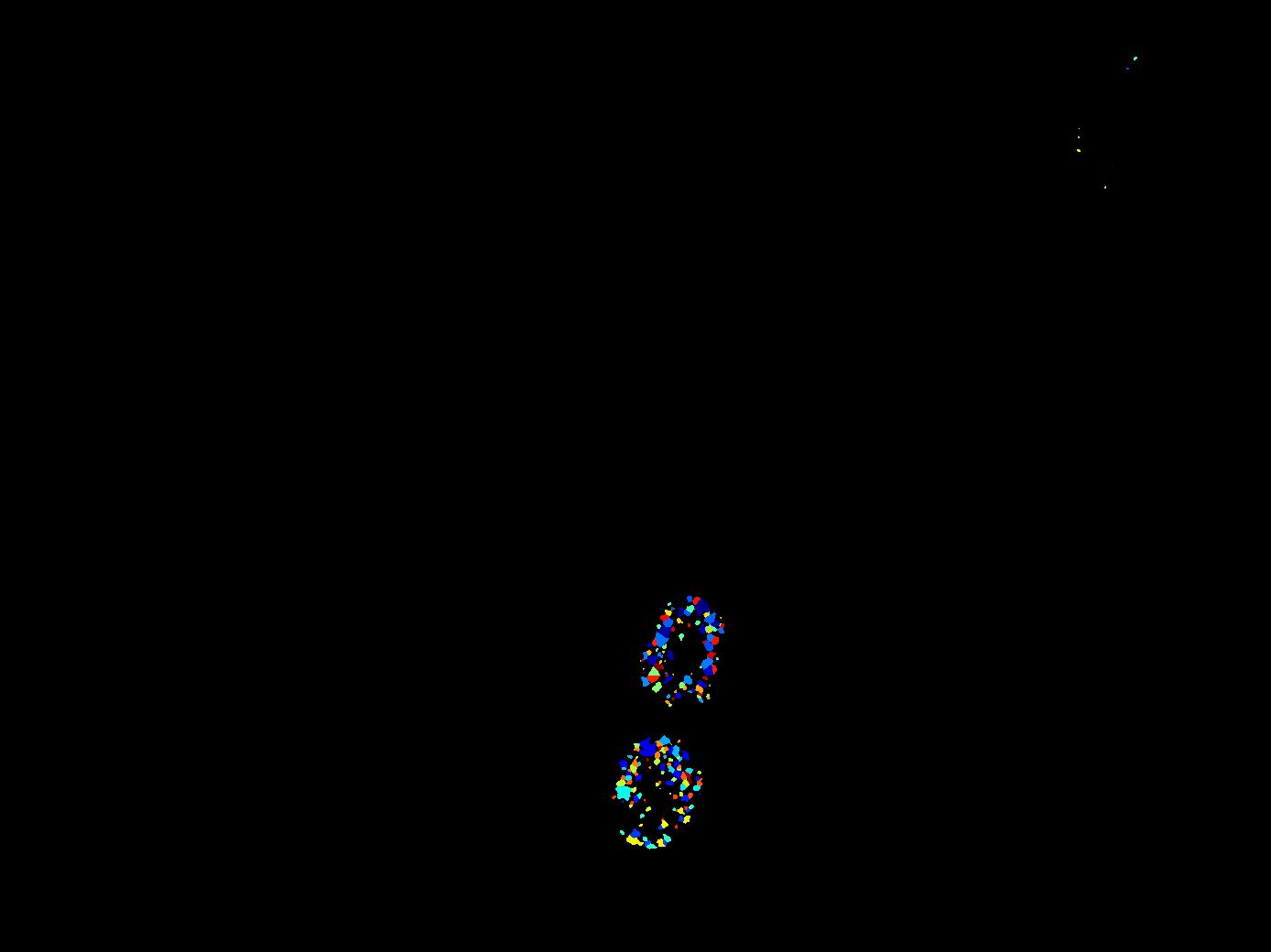

Supplement: Supplementary file 7 — Source Data [file 41467_2022_28822_MOESM7_ESM.zip › Figure 5E data/Masks/K136Q_I_13K136Q_24h_13_.jpeg]

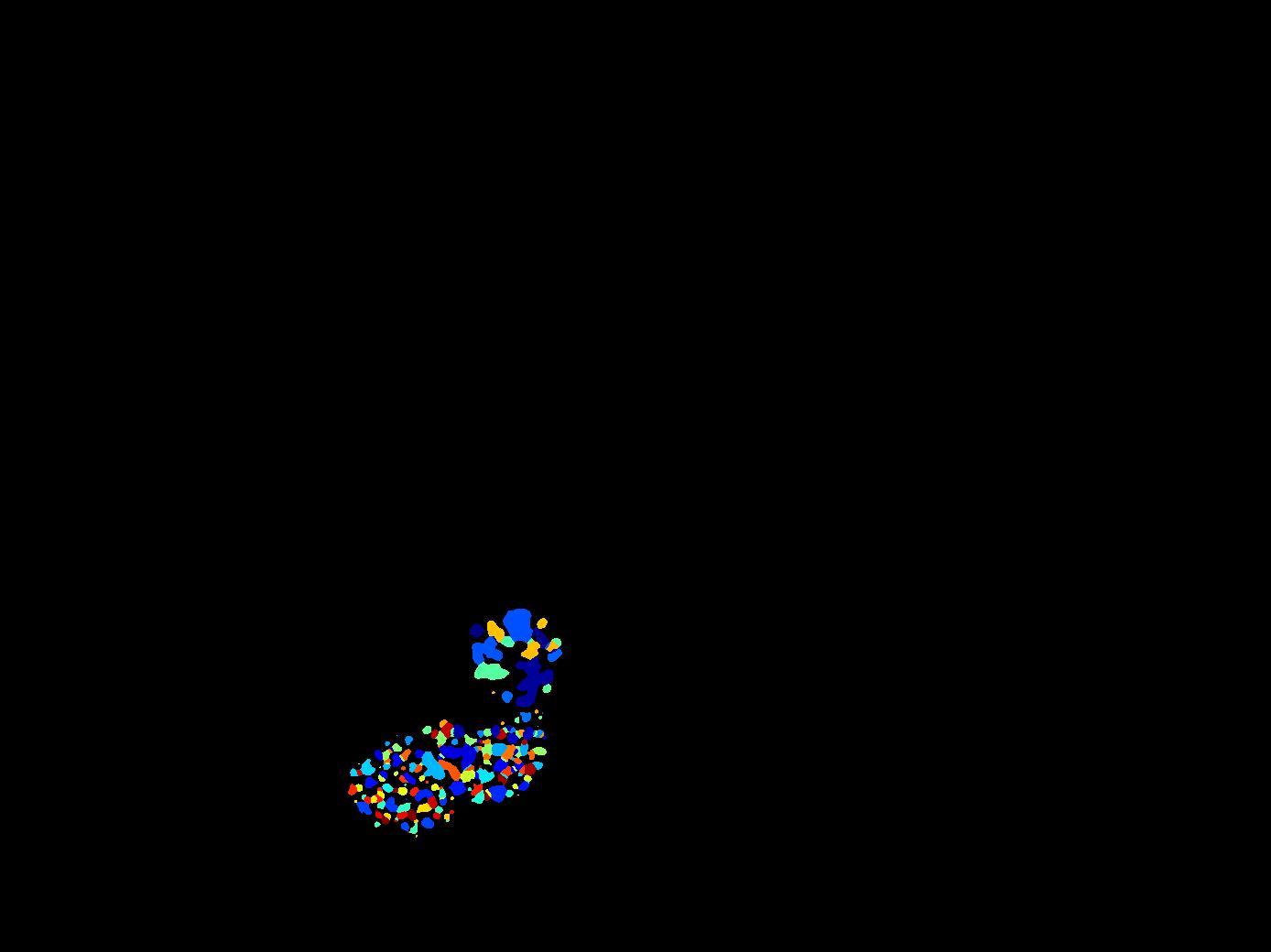

Supplement: Supplementary file 7 — Source Data [file 41467_2022_28822_MOESM7_ESM.zip › Figure 5E data/Masks/K136Q_I_13K136Q_48h 2_13_.jpeg]

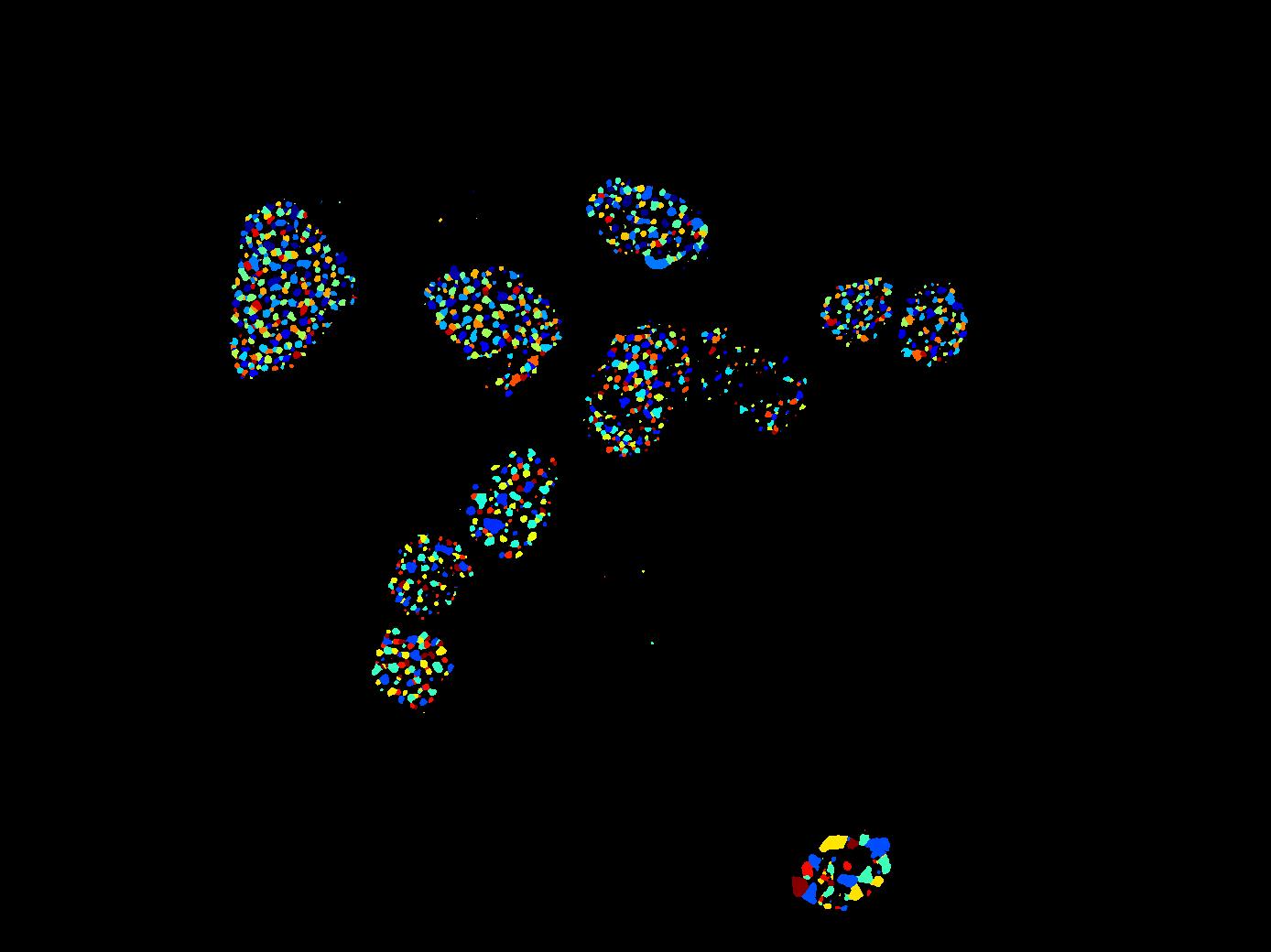

Supplement: Supplementary file 7 — Source Data [file 41467_2022_28822_MOESM7_ESM.zip › Figure 5E data/Masks/K136Q_I_13K136Q_48h_13_.jpeg]

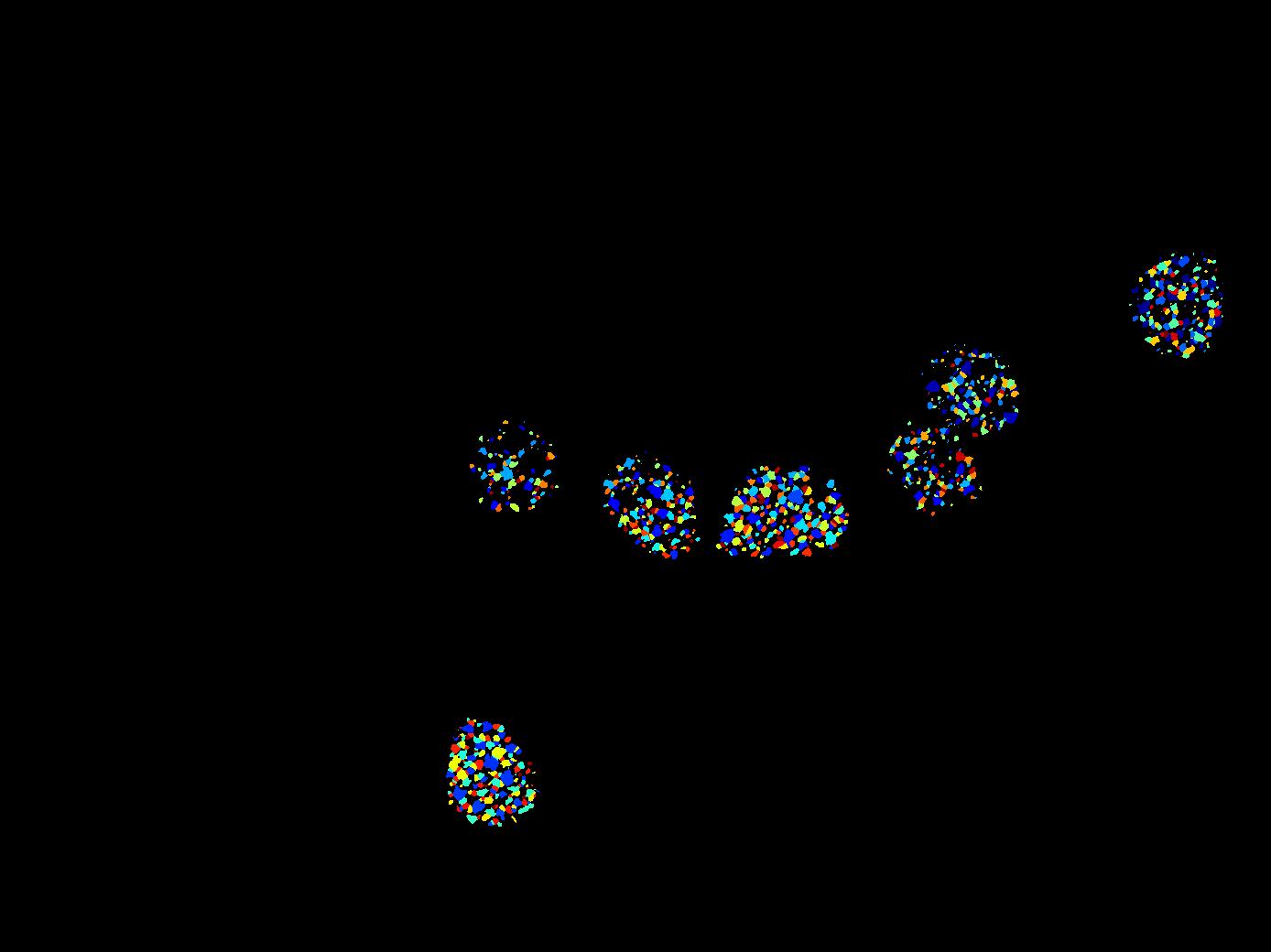

Supplement: Supplementary file 7 — Source Data [file 41467_2022_28822_MOESM7_ESM.zip › Figure 5E data/Masks/K136Q_I_14K136Q_24h 2_14_.jpeg]

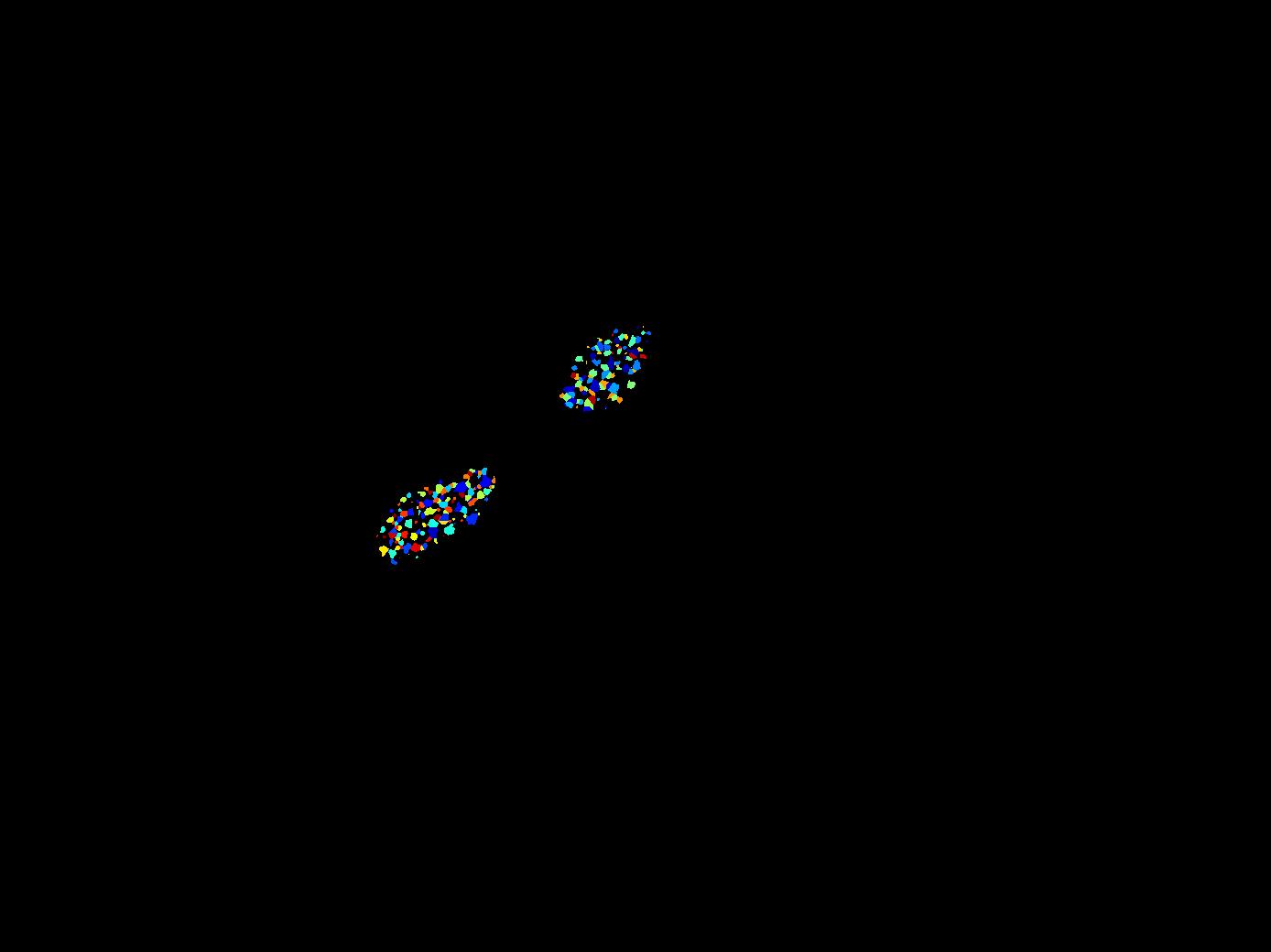

Supplement: Supplementary file 7 — Source Data [file 41467_2022_28822_MOESM7_ESM.zip › Figure 5E data/Masks/K136Q_I_14K136Q_24h_14_.jpeg]

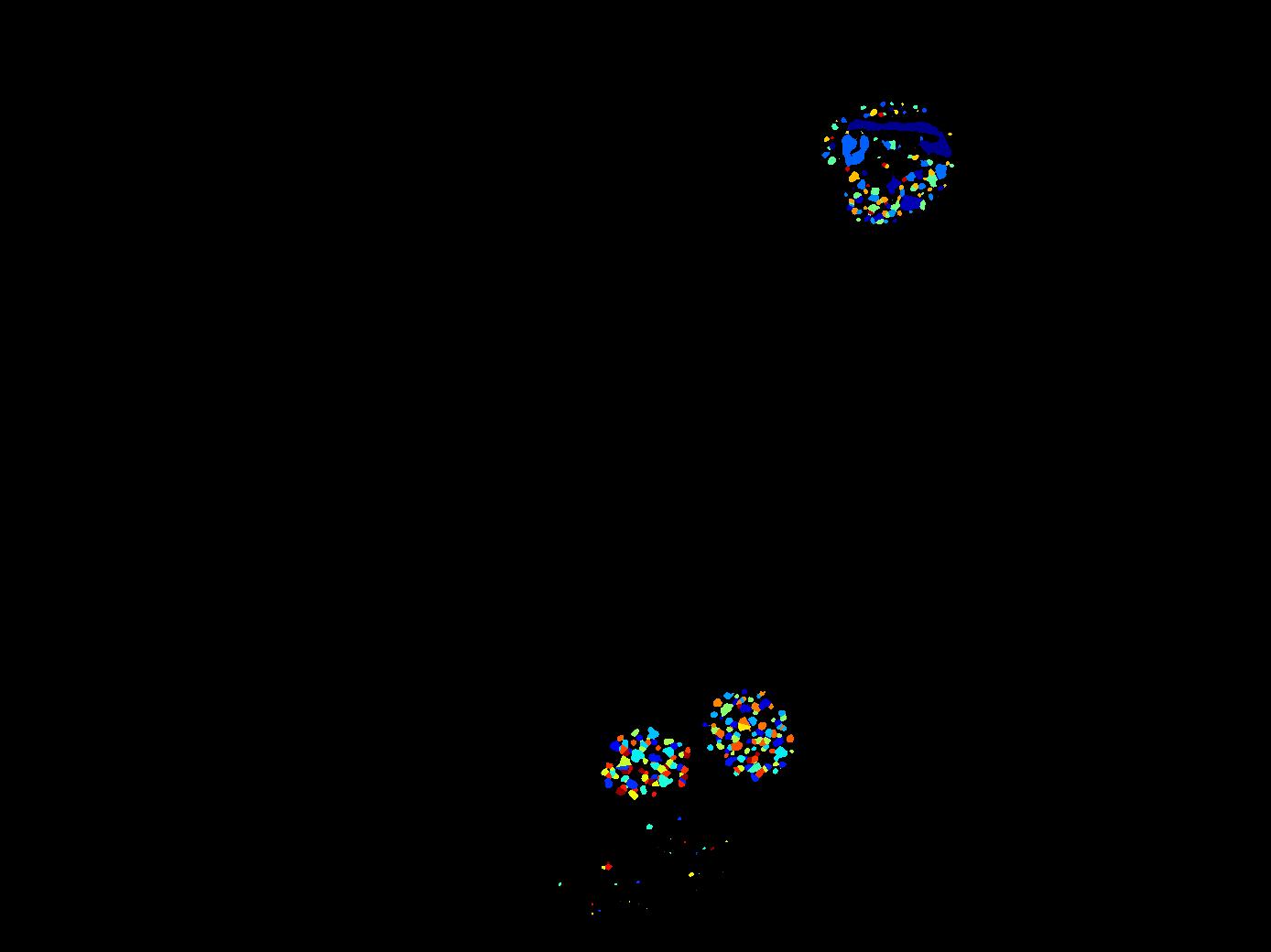

Supplement: Supplementary file 7 — Source Data [file 41467_2022_28822_MOESM7_ESM.zip › Figure 5E data/Masks/K136Q_I_14K136Q_48h 2_14_.jpeg]

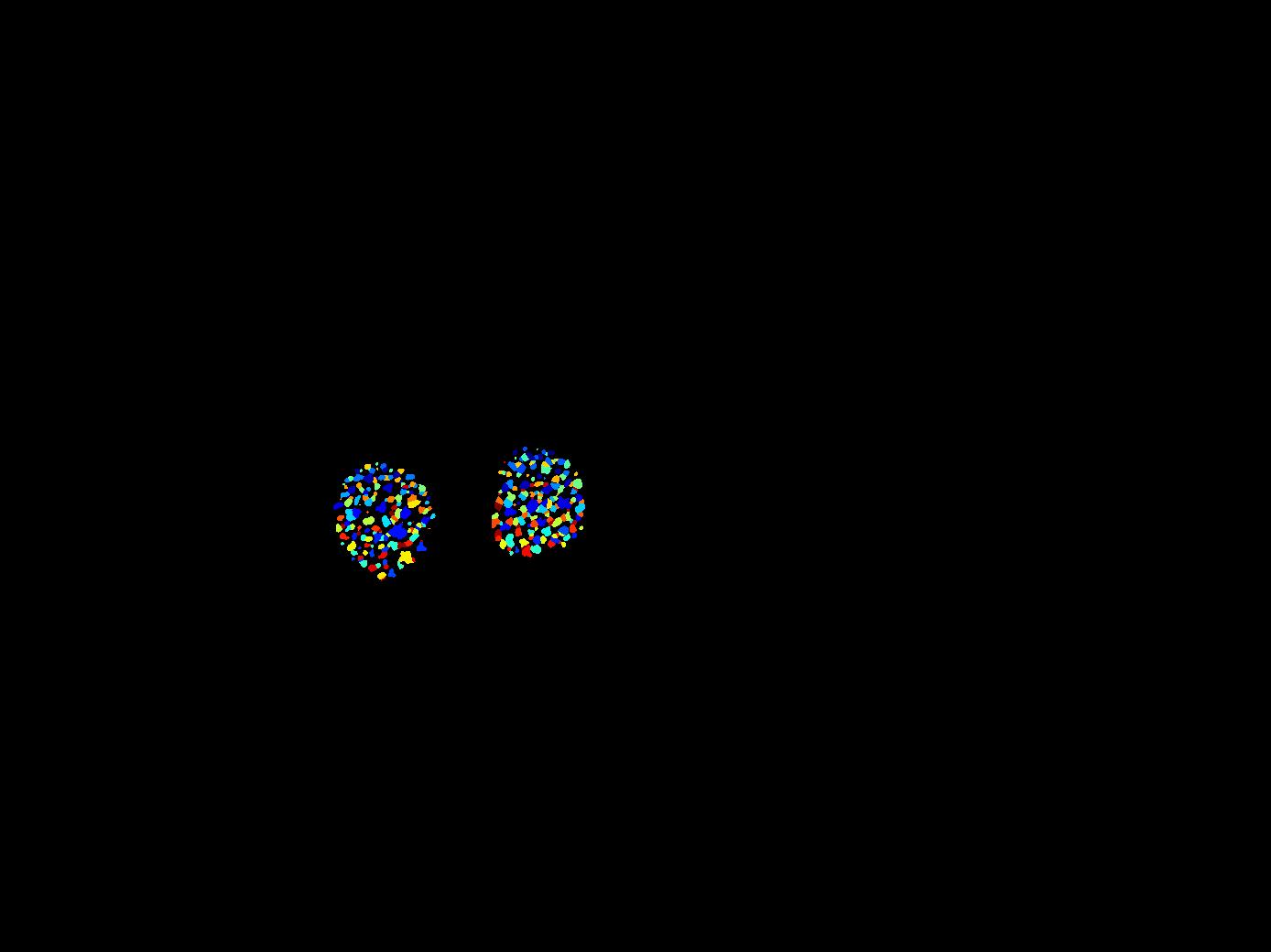

Supplement: Supplementary file 7 — Source Data [file 41467_2022_28822_MOESM7_ESM.zip › Figure 5E data/Masks/K136Q_I_15K136Q_24h 2_15_.jpeg]

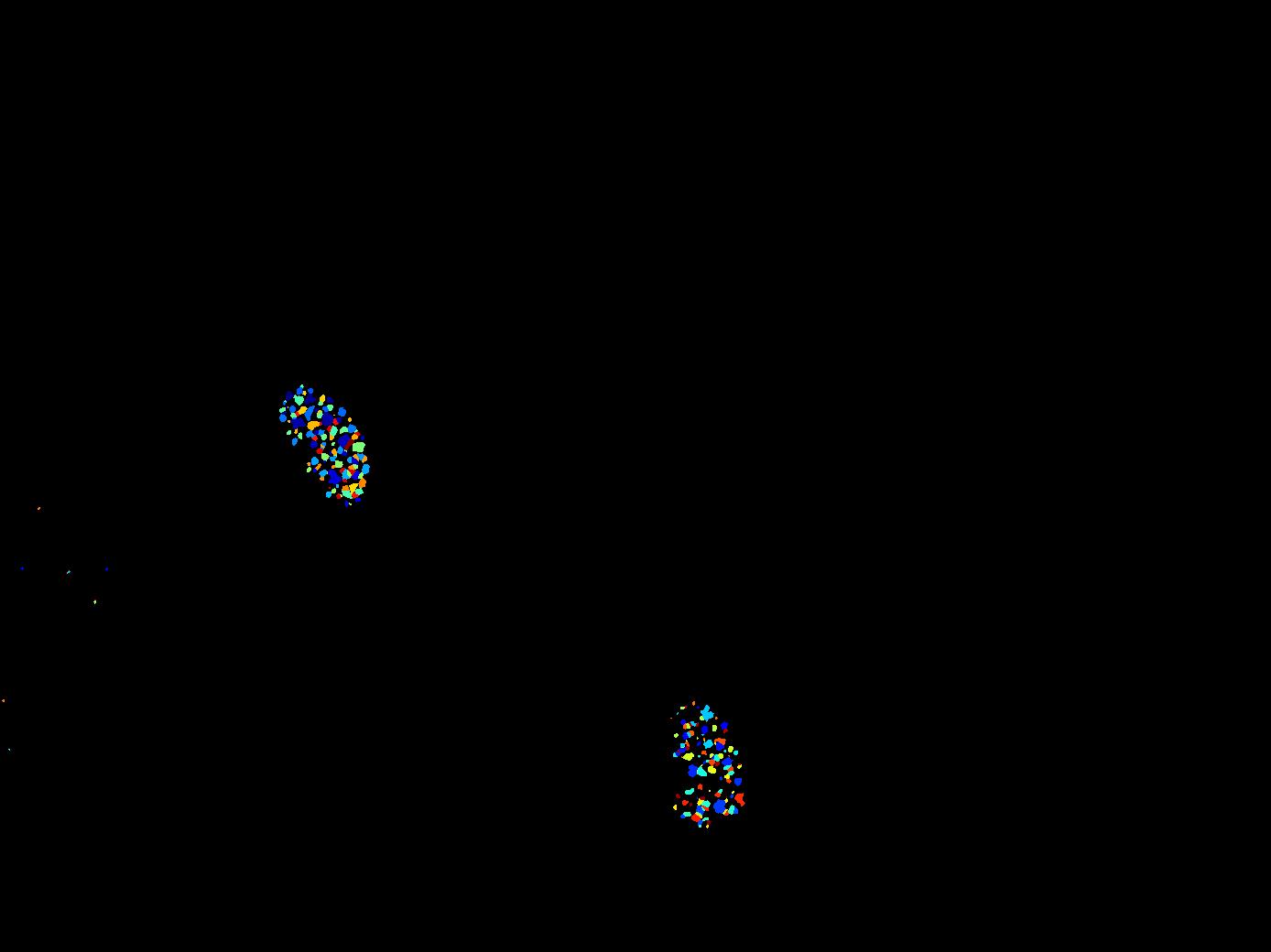

Supplement: Supplementary file 7 — Source Data [file 41467_2022_28822_MOESM7_ESM.zip › Figure 5E data/Masks/K136Q_I_15K136Q_24h_15_.jpeg]

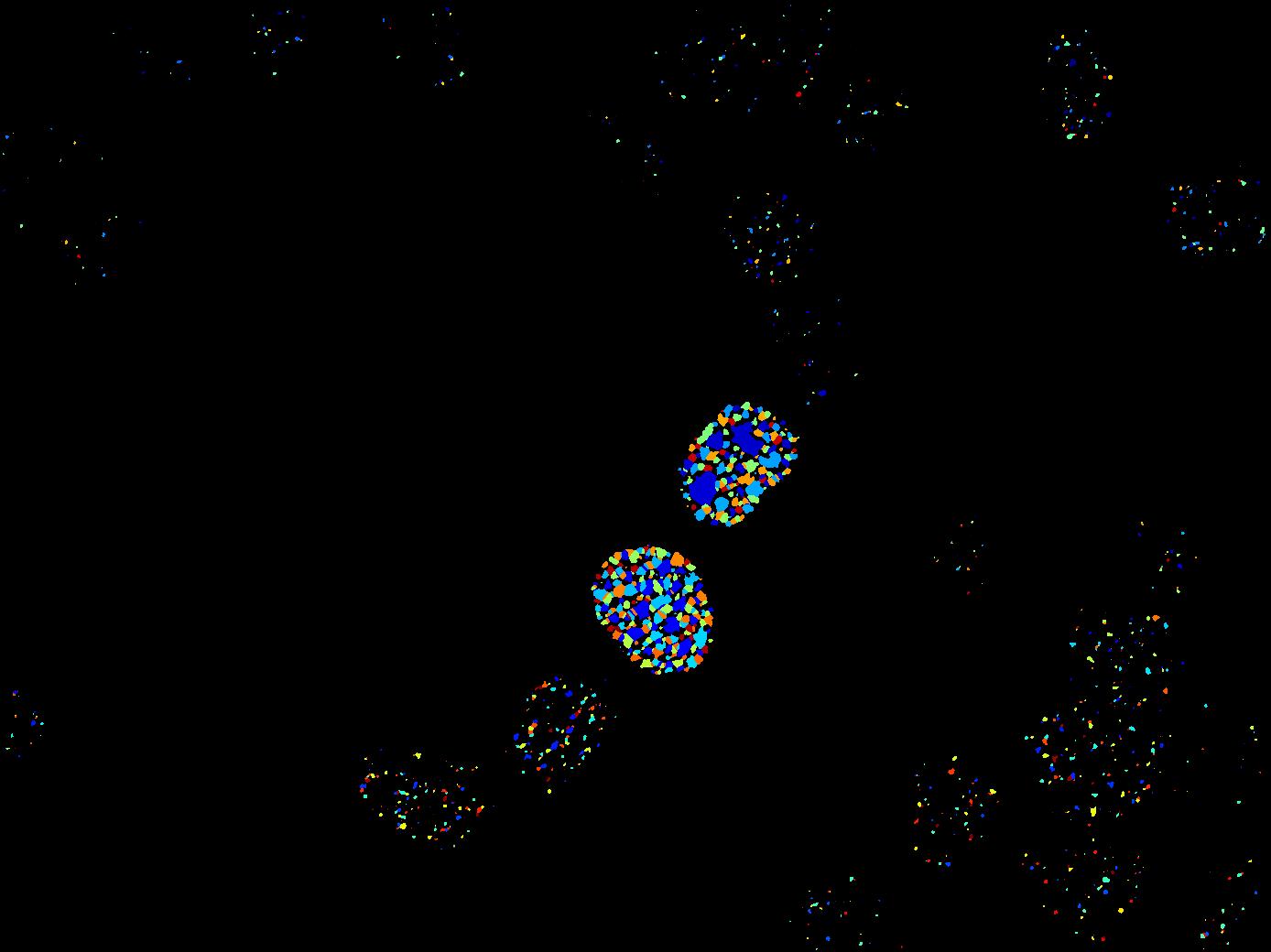

Supplement: Supplementary file 7 — Source Data [file 41467_2022_28822_MOESM7_ESM.zip › Figure 5E data/Masks/K136Q_I_16K136Q_24h 2_16_.jpeg]

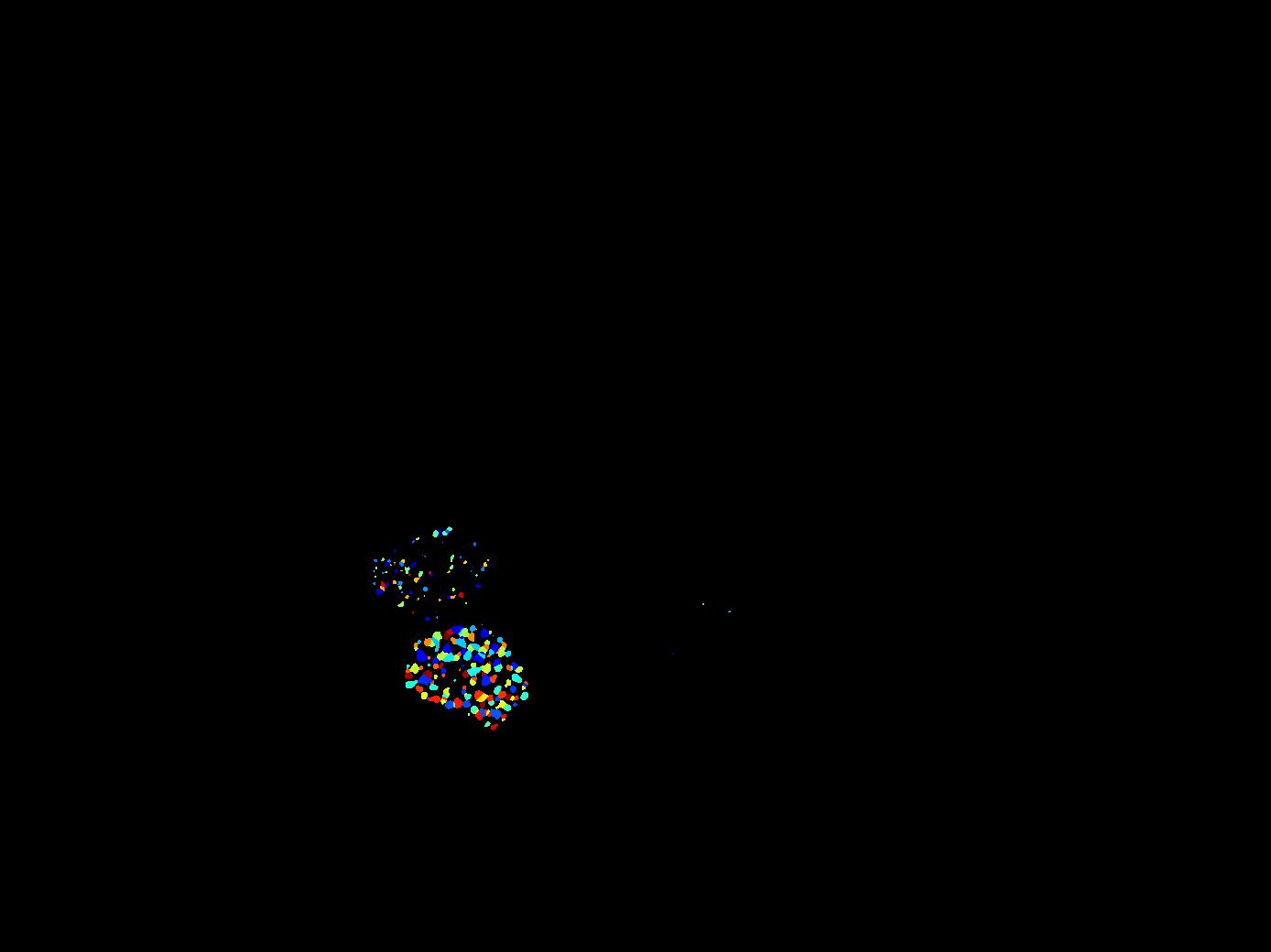

Supplement: Supplementary file 7 — Source Data [file 41467_2022_28822_MOESM7_ESM.zip › Figure 5E data/Masks/K136Q_I_16K136Q_24h_16_.jpeg]

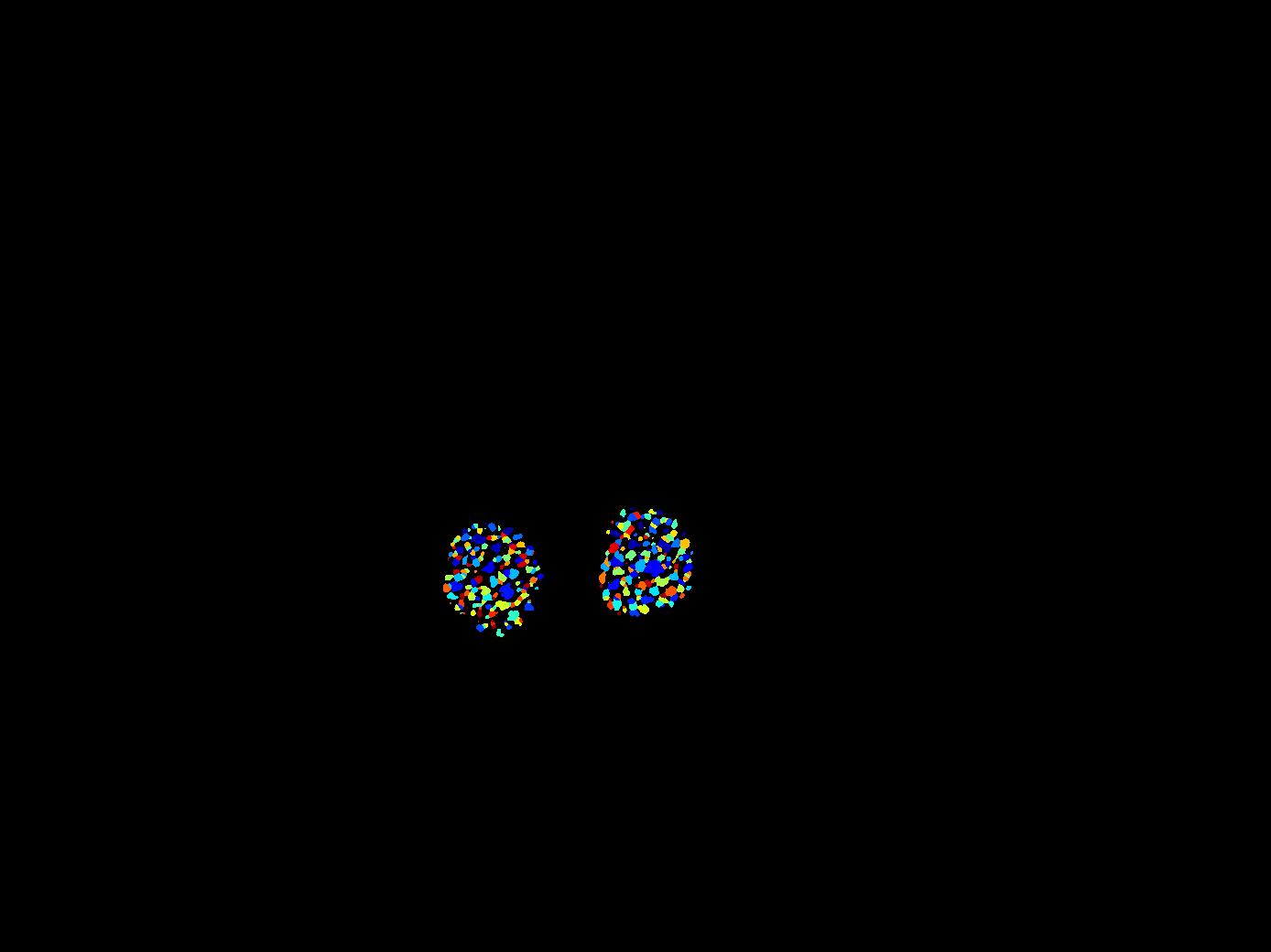

Supplement: Supplementary file 7 — Source Data [file 41467_2022_28822_MOESM7_ESM.zip › Figure 5E data/Masks/K136Q_I_17K136Q_24h 2_17_.jpeg]

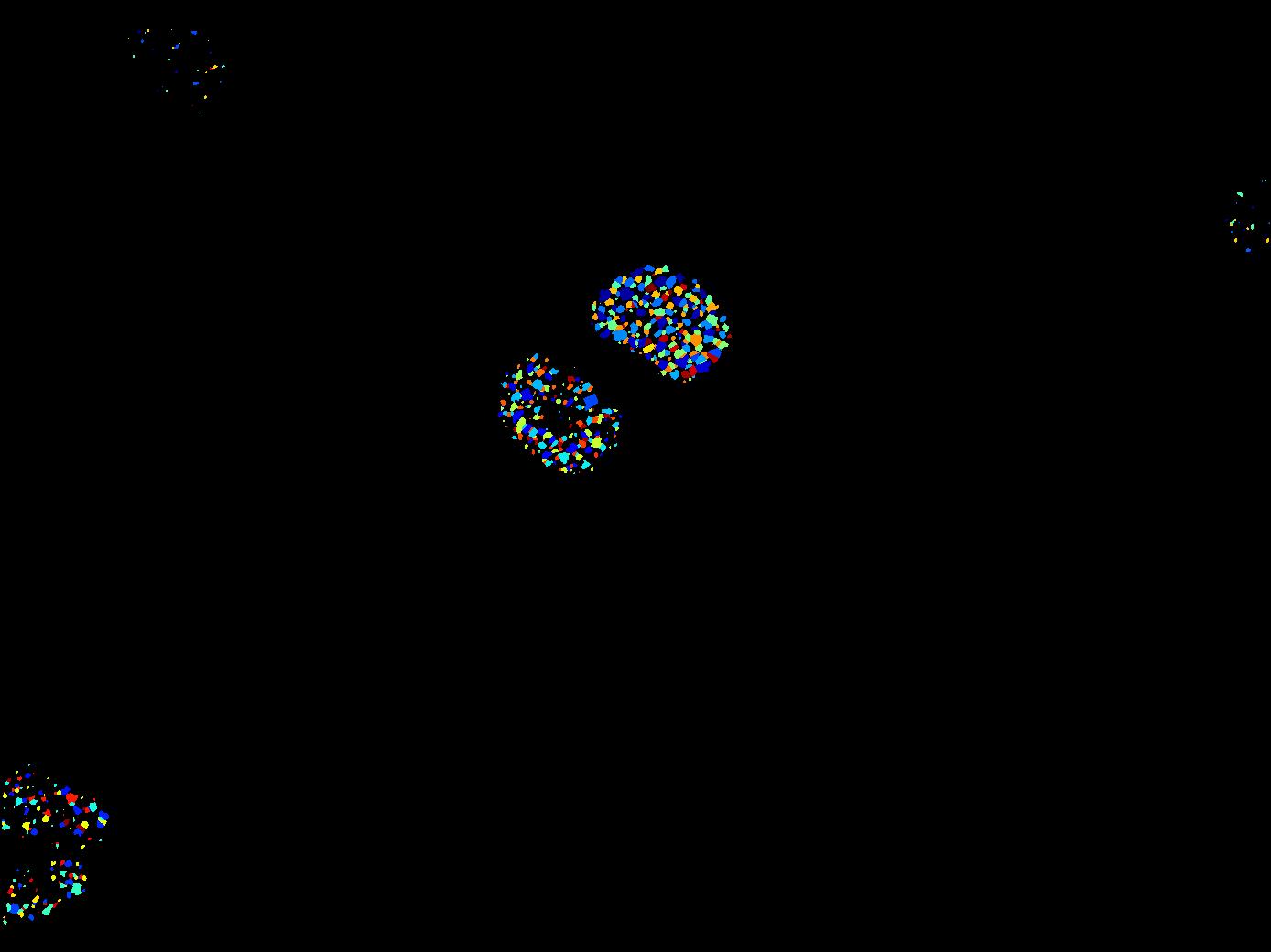

Supplement: Supplementary file 7 — Source Data [file 41467_2022_28822_MOESM7_ESM.zip › Figure 5E data/Masks/K136Q_I_17K136Q_24h_17_.jpeg]

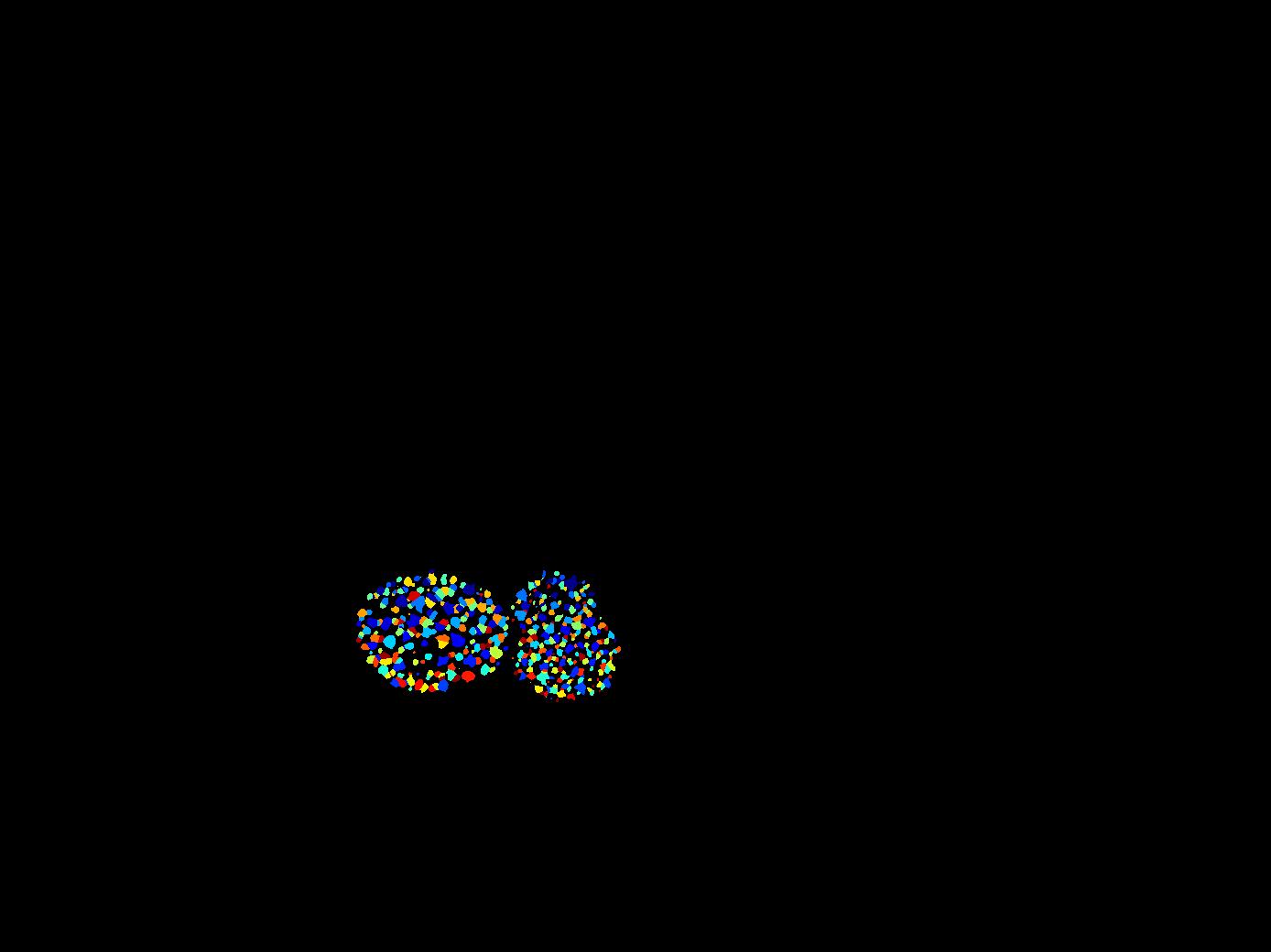

Supplement: Supplementary file 7 — Source Data [file 41467_2022_28822_MOESM7_ESM.zip › Figure 5E data/Masks/K136Q_I_18K136Q_24h 2_18_.jpeg]

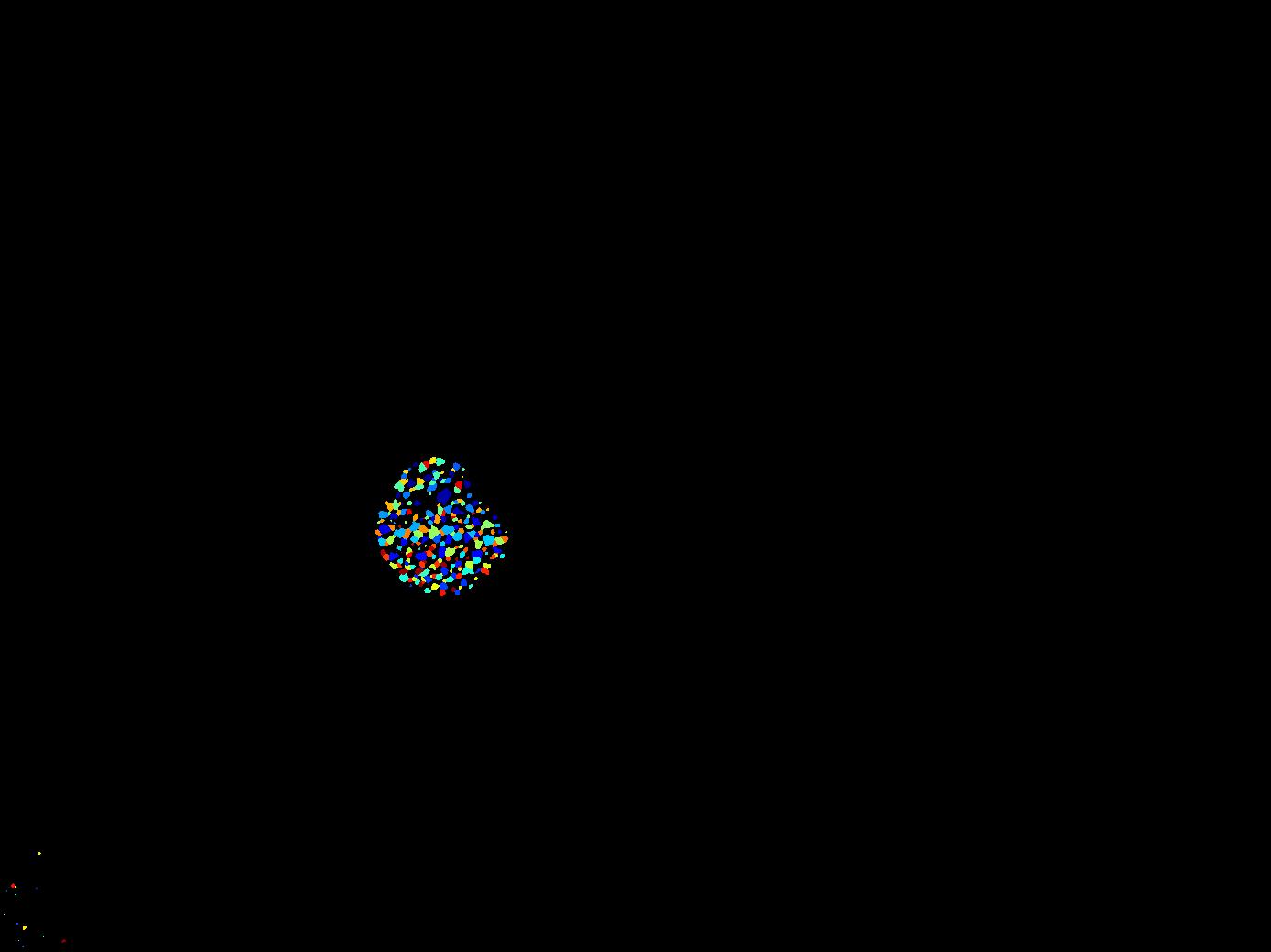

Supplement: Supplementary file 7 — Source Data [file 41467_2022_28822_MOESM7_ESM.zip › Figure 5E data/Masks/K136Q_I_19K136Q_24h 2_19_.jpeg]

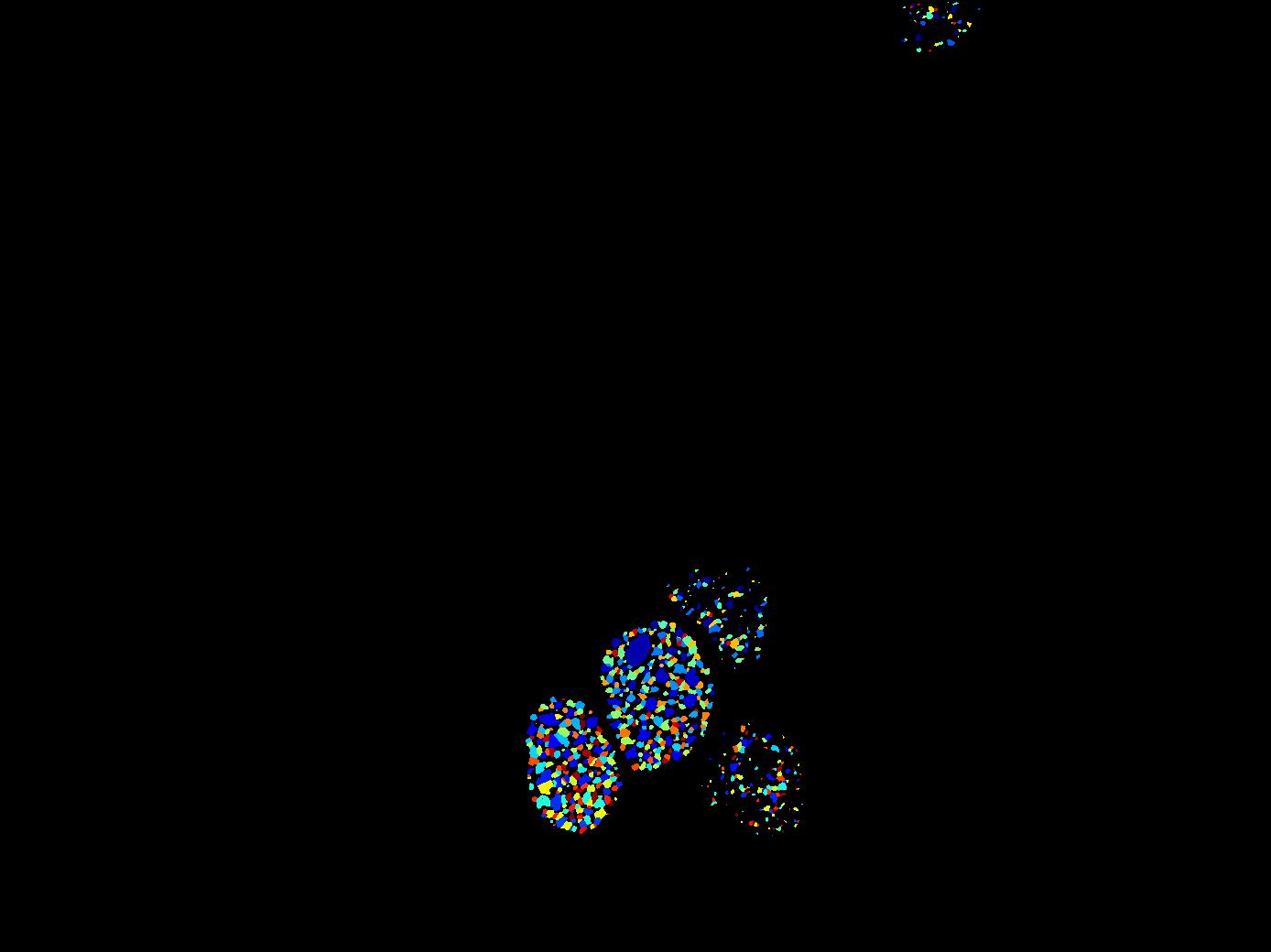

Supplement: Supplementary file 7 — Source Data [file 41467_2022_28822_MOESM7_ESM.zip › Figure 5E data/Masks/K136Q_I_20K136Q_24h 2_20_.jpeg]

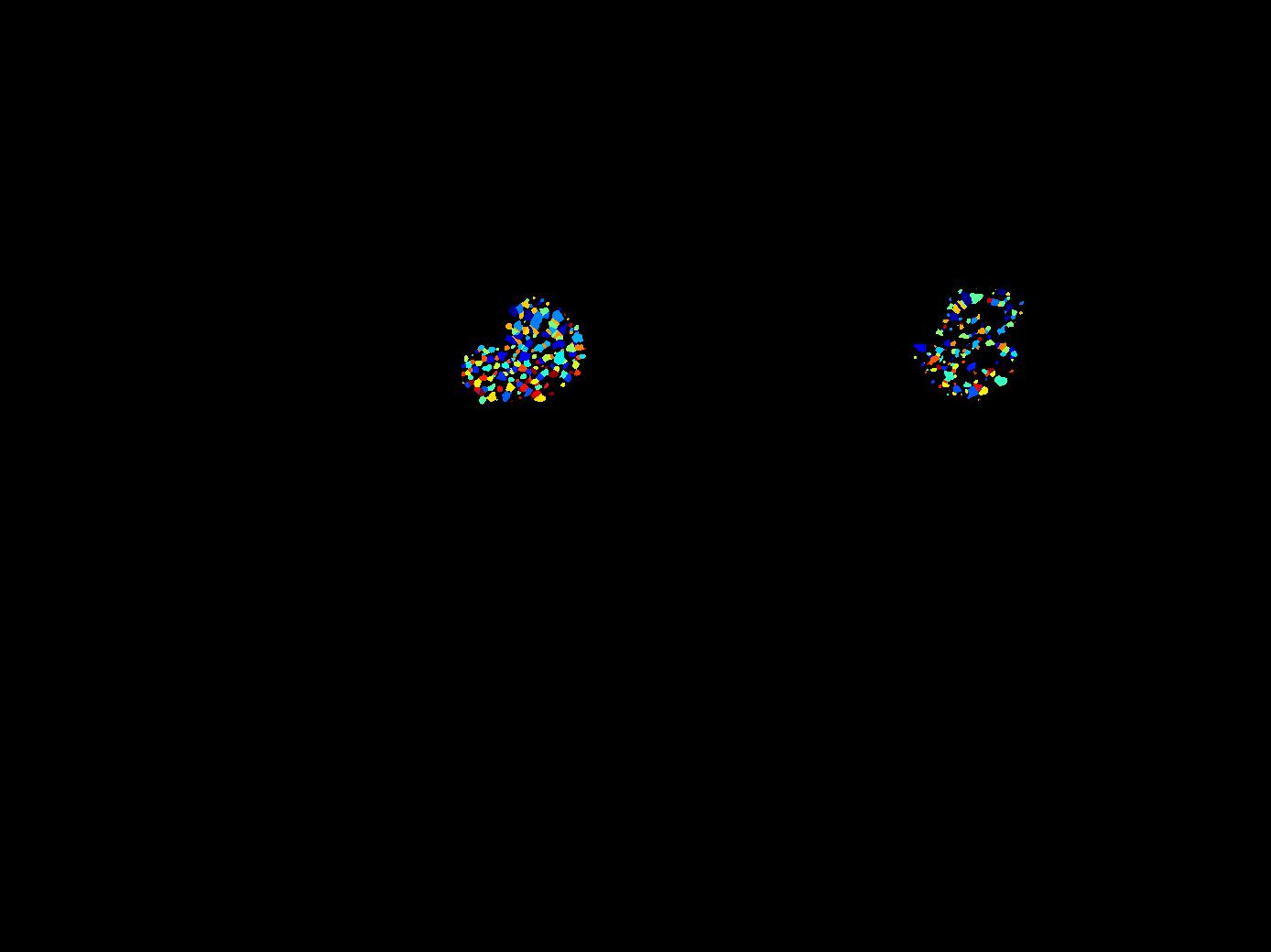

Supplement: Supplementary file 7 — Source Data [file 41467_2022_28822_MOESM7_ESM.zip › Figure 5E data/Masks/K136Q_I_21K136Q_24h 2_21_.jpeg]

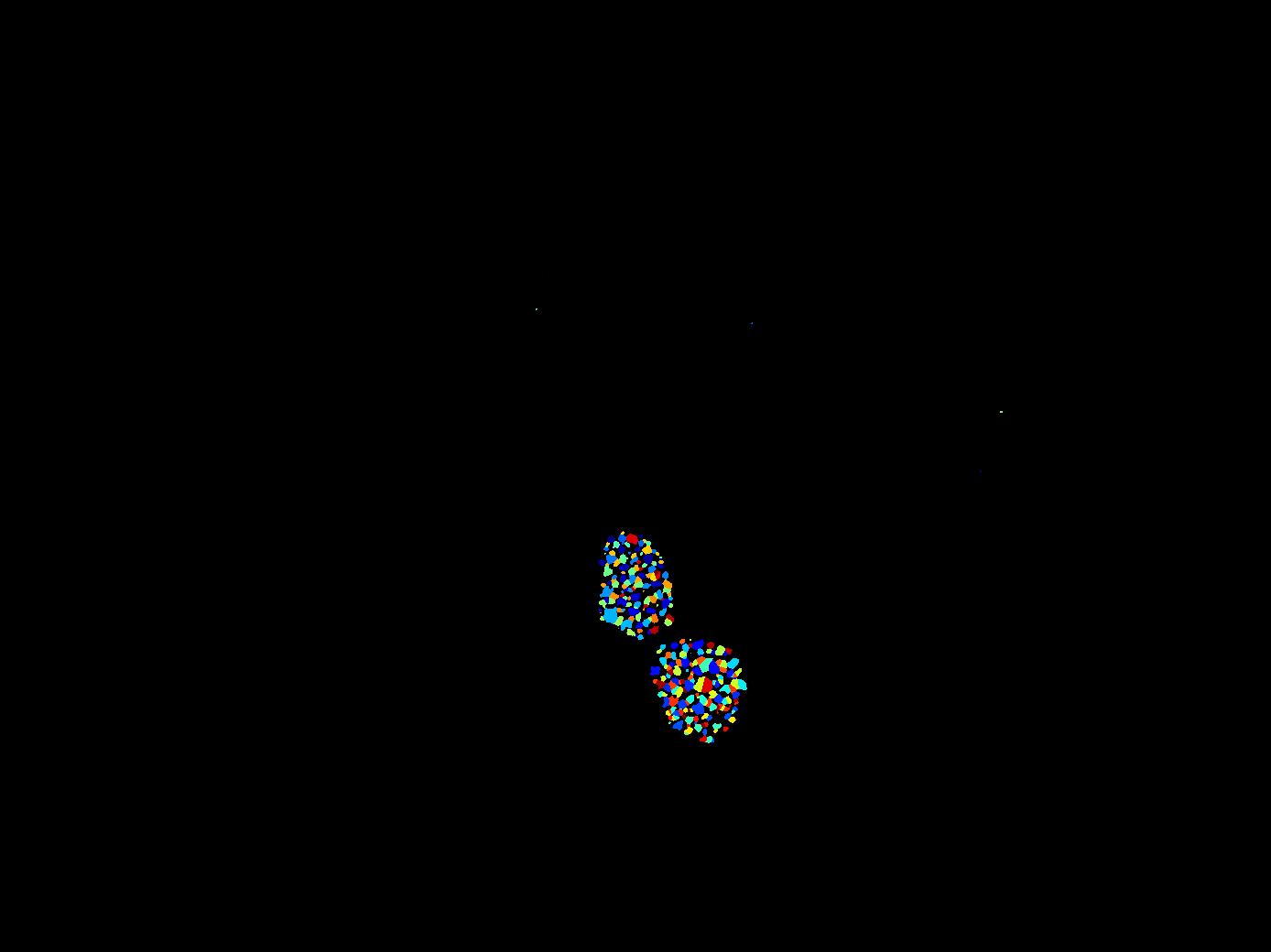

Supplement: Supplementary file 7 — Source Data [file 41467_2022_28822_MOESM7_ESM.zip › Figure 5E data/Masks/K136Q_I_22K136Q_24h 2_22_.jpeg]

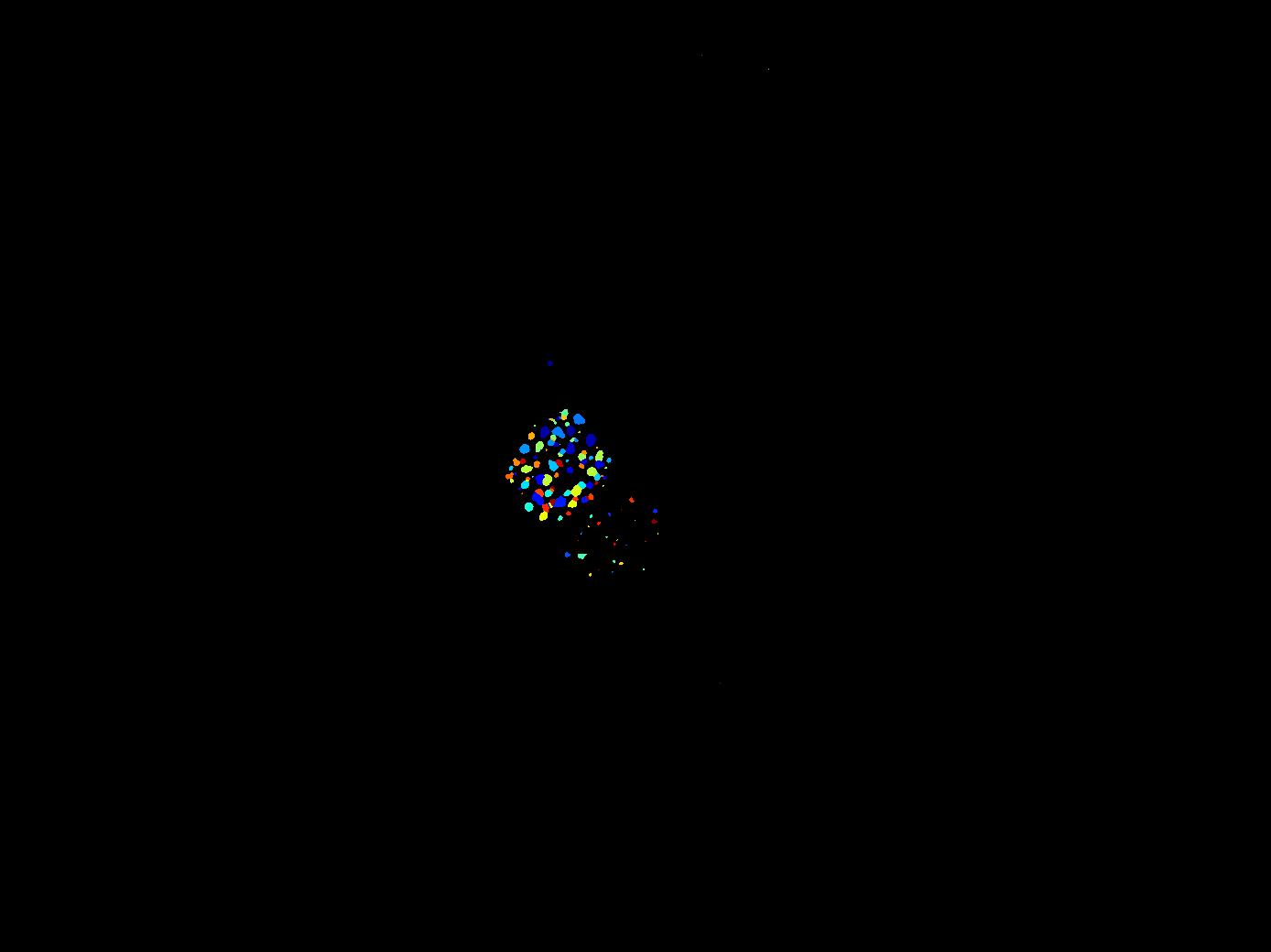

Supplement: Supplementary file 7 — Source Data [file 41467_2022_28822_MOESM7_ESM.zip › Figure 5E data/Masks/K136Q_I_23K136Q_24h 2_23_.jpeg]

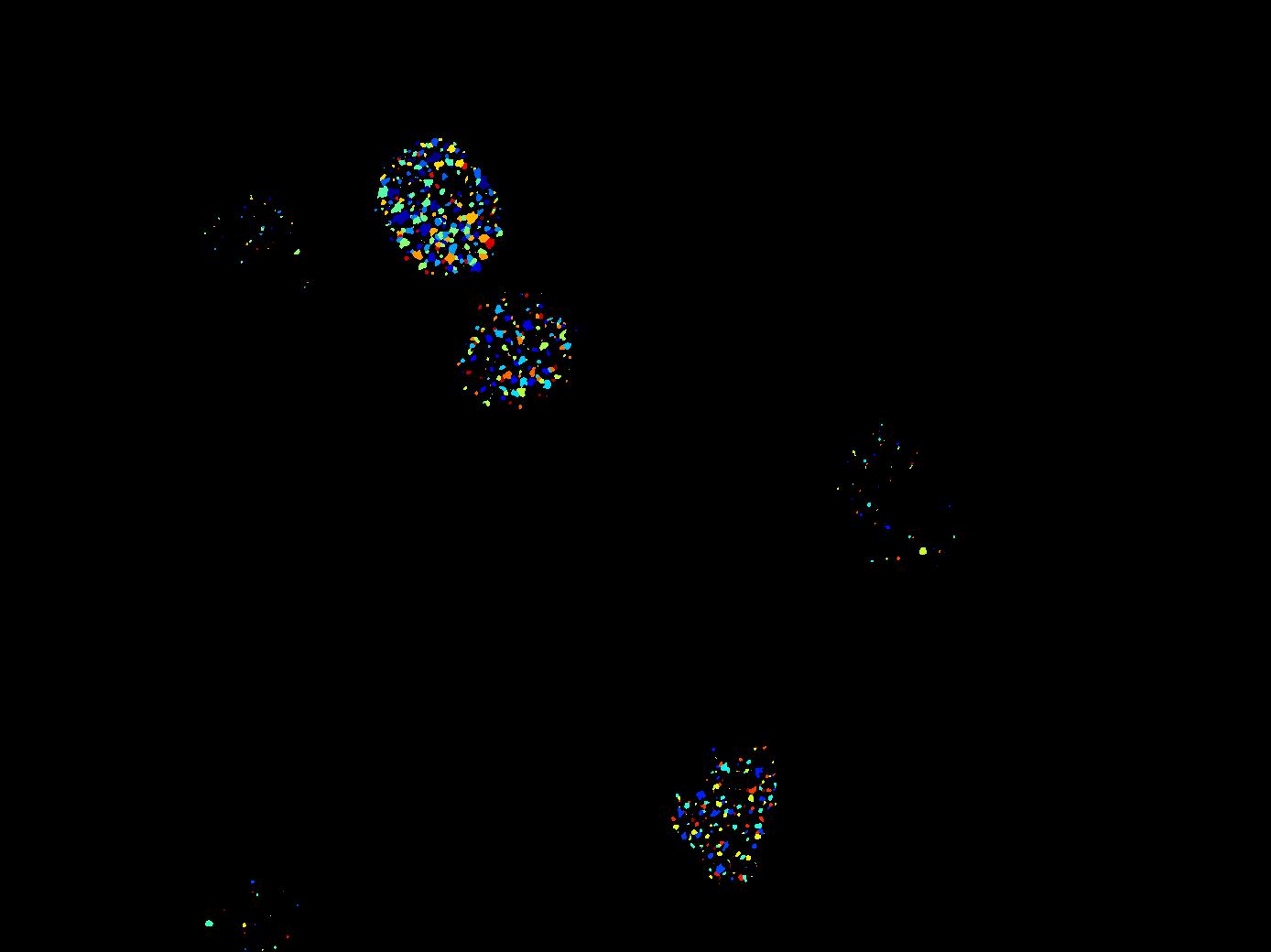

Supplement: Supplementary file 7 — Source Data [file 41467_2022_28822_MOESM7_ESM.zip › Figure 5E data/Masks/K136Q_I_24K136Q_24h 2_24_.jpeg]
